# Stable generation of serum- and feeder-free embryonic stem cell-derived mice with full germline-competency by using a GSK3 specific inhibitor

**DOI:** 10.1002/dvg.20514

**Published:** 2009-04-23

**Authors:** Hiromu Sato, Keiko Amagai, Rie Shimizukawa, Yoshitaka Tamai

**Affiliations:** Tsukuba Research Institute, Banyu Pharmaceutical Co., Ltd.Tsukuba, Japan

**Keywords:** feeder free, serum free, embryonic stem cell, BIO, C57BL/6

## Abstract

C57BL/6 (B6)-derived embryonic stem (ES) cells are not widely used to generate knockout mice despite the advantage of a well-defined genetic background because of poor developmental potential. We newly established serum- and feeder-free B6 ES cells with full developmental potential by using leukemia inhibitory factor (LIF) and 6-bromoindirubin-3′-oxime (BIO), a glycogen synthase kinase-3 (GSK3) inhibitor. BIO treatment significantly increased the expression levels of 364 genes including pluripotency markers such as Nanog and Klf family. Unexpectedly, by aggregating or microinjecting those ES cells to each eight-cell-stage diploid embryo, we stably generated germline-competent ES-derived mice. Furthermore, founder mice completely derived from female XO, heterozygous, or homozygous mutant B6 ES cells were directly available for intercross breeding and phenotypic analysis. We hereby propose that serum- and feeder-free B6 ES cells stimulated with LIF plus GSK3 inhibitor are valuable for generating mouse models on B6 background. genesis 47:414–422, 2009. © 2009 Wiley-Liss, Inc.

## INTRODUCTION

The technology to generate knockout (KO) mice has been one of the most important advances in understanding the function of genes of interest (Capecchi, 2001; Evans, 2001; Goldstein, 2001; Smithies, 2001). The establishment of KO mice requires time-consuming processes; a particularly laborious step is the acquisition of germline-competent founder generation mice from mutant embryonic stem (ES) cells. Generally, the conventional blastocyst injection method provides founder mice that are only partially derived from mutant ES cells. If mutant ES cells partially differentiate into germ cells, the founder mice can transmit the mutation to the next generation of mice. C57BL/6 (B6)-derived ES cells have a greater tendency than 129-derived ES cells to lose their ability to colonize the germline (Auerbach *et al*., 2000; Shimizukawa *et al*., 2005), although B6 strain is preferred for phenotyping in vivo especially in metabolic disorder, behavior and cognition, and immune system studies. Alternatively, the tetraploid embryo complementation method has been used to generate germline-competent founder mice that are entirely derived from ES cells. Unfortunately, the founder mice derived from inbred ES cells, such as the B6 strain, show poor viabilities or abnormalities due to developmental defects (Eggan *et al*., 2001; Nagy *et al*., 1993). The developmental potential of B6 ES cells seems to be lost during cell culture in vitro (Eggan *et al*., 2001), which is dependent on the serum and feeder cells. One possibility is that the quality of the serum lot and feeder layers is variable in each culture, which may cause decreased developmental potential. Therefore, we speculate that if ES cells are maintained under serum- and feeder-free conditions, germline-competent founder mice should be stably generated even from B6 ES cells. However, ES cell culture methods without both serum and feeder cells have not been thoroughly established.

Leukemia inhibitory factor (LIF)/STAT3 signaling is known to suppress lineage commitment (Cartwright *et al*., 2005; Matsuda *et al*., 1999; Niwa *et al*., 1998), but is insufficient for maintaining self-renewal and pluripotency of ES cells under a serum- and feeder-free condition. ES cells cultured with LIF-containing medium partially and spontaneously differentiate to early primitive ectoderm cells, which shows that they have a low ability to contribute to germ cells (Chambers *et al*., 2007; Toyooka *et al*., 2008). These findings suggest that other factor(s) contained in the serum and/or produced by feeder cells may support the undifferentiation of ES cells.

As an attractive candidate instead of serum and feeder cells, we focused on the inhibition of glycogen synthase kinase-3 (GSK3) in Wnt/β-catenin signaling, which maintains self-renewal and pluripotency in both mouse and human ES cells cultured in the presence of serum (Sato *et al*., 2004; Singla *et al*., 2006). According to a previous report, GSK3α/β double-KO ES cells display hyperactivated Wnt/β-catenin signaling, and severely impair differentiation into three germ layers (endoderm, ectoderm, and mesoderm) (Doble *et al*., 2007). However, it has not been examined whether GSK3 inhibition fulfills the requirement for serum and feeder cells together with LIF/STAT3 signaling.

In this study, we investigated: (1) whether LIF/STAT3 signaling plus GSK3 inhibition maintain self-renewal and pluripotency of ES cells cultured in the absence of serum and feeder layers, and (2) whether germline-competent founder mice are generated from ES cells, which were maintained in such culture conditions. In a previous report, GSK3β deficient embryos were pale and non-viable between E 13.5 and E 14.5 (Hoeflich *et al*., 2000). We have used 6-bromoindirubin-3′-oxime (BIO), a characterized GSK3 inhibitor, for reversible control of GSK3 function.

## RESULTS

### The Treatment of LIF Plus BIO Enhanced Self-Renewal and Pluripotency of Serum- and Feeder-Free B6 ES Cells

**FIG. 1:**
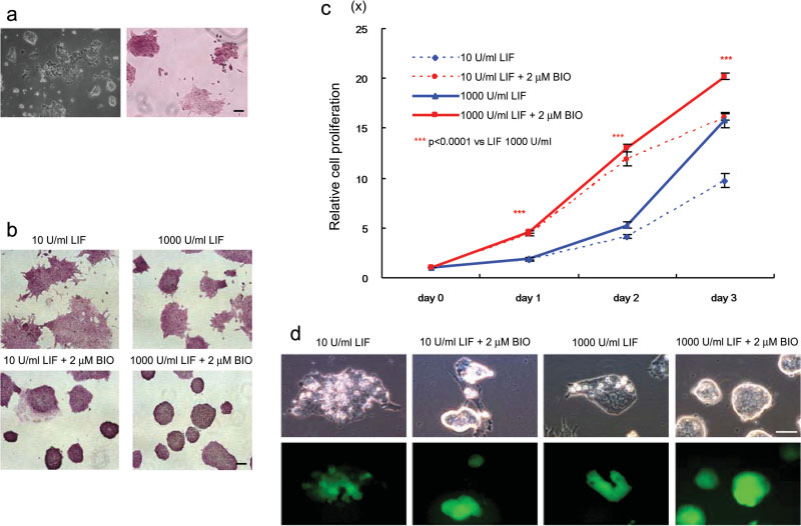
Establishment of serum- and feeder-free B6 ES cells by using LIF and BIO. (a) Morphology (left) and alkaline phosphatase activity (right) of serum- and feeder-free B6 ES cells treated with 1000 U/ml LIF alone. (b) Alkaline phosphatase activity of B6 ES cells cultured for 5 days under the serum- and feeder-free condition. (c) Cell growth assay. Relative cell proliferation was measured as a standard at Day 0. Error bars are standard deviations (*n* = 6). ***P > 0.0001, *t*-test, between 1000 U/ml LIF and 1000 U/ml LIF plus 2 μM BIO. (d) GFP expression under Oct4 promoter in serum- and feeder-free B6 ES cells cultured for 5 days. Scale bar: 100 μm.

C57/BL6J ES cells (TB6-2), which were originally established by culturing the inner cell mass (ICM) of blastocyst-stage embryos, were maintained on a feeder layer in 1000 U/ml LIF plus 15% serum-containing medium as a standard culture method. However, these cells showed very low developmental potency despite normal karyotypes (38 + XY) when aggregated with tetraploid embryos to generate founder mice (2 100% ES-derived coat-color mice/427 embryos transferred, 0.5%; Table 1). Tetraploid aggregation method was useful for the generation of founder mice from B6-derived ES cells (Shimizukawa *et al*., 2005). To improve their potency, we cultured TB6-2 cells on a gelatin-coated plate in 1000 U/ml LIF plus 15% Knockout Serum Replacement (KSR) medium, and they were maintained under “serum- and feeder-free” or “serum- and feeder-independent” conditions. Compared with a standard culture system, most colonies showed alkaline phosphatase activity, whereas some of them did not (Fig. 1a). This result indicates that the TB6-2 cells consisted of heterogeneous populations due to spontaneous differentiation.

**TABLE 1:** Comparison of Developmental Potential Among Various ES Cells Cultured with 10 U/ml (L10), 1000 U/ml LIF (L1000), 2 mM BIO (BIO), or 40 mM PD98059 (PD)

ES cells							No. of live offsprings					
								ES cell contribution in coat color				
Strain	No. of passages	Genotype	Culture condition	Host embryo strain (ploidy)	Method	No. of embryos transferred	Total	0%	<100%	100%	Rate of 100% ES-derived oat-color mice in live offsprings (%)	Germline transmission
B6 (TB6-2)	8	+/+	Serum 1 Feeder cells 1 L1000	ICR (4N)	Aggregation	427	2	0	0	2	100.0	n.d.
B6	12	+/+	L10	ICR (2N)	Aggregation	88	43	43	0	0	0.0	n.d
B6	12	+/+	L10 + BIO	ICR (2N)	Aggregation	100	15	12	0	3	20.0	n.d.
B6	12	+/+	L 1000	ICR (2N)	Aggregation	87	16	8	3	5	31.3	n.d
B6	12	+/+	L 1000+PD	ICR (2N)	Aggregation	63	10	8	0	2	20.0	2/2(100%)
B6	12	+/+	L 1000 + BIO	ICR (2N)	Aggregation	111	12	1	0	11	91.7	1/1(100%)
B6	12	+/+	L 1000 + BIO + PD	ICR (2N)	Aggregation	63	16	1	0	15	93.8	4/4(100%)
B6	12	+/+	L 1000 + BIO + PD	ICR (4N)	Aggregation	75	18	0	0	18	100	2/2(100%)
B6	12	+/+	L 1000 + BIO + PD	ICR (2N)	Conventional injection	44	6	1	0	5	83.3	n.d.
B6	12	+/+	L 1000 + BIO + PD	ICR (2N)	Laser-assited injection	60	8	1	0	7	87.5	n.d.
V6.5 (B6/129 F1 hybrid)	18	+/+	Serum 1 Feeder cells 1 L1000	ICR (4N)	Aggregation	270	19	0	0	19	100.0	n.d
V6.5	22	+/+	L1000 + BIO	ICR (2N)	Aggregation	42	10	0	0	10	100.0	2/2 (100%)
V6.5	22	+/+	L1000 1 BIO 1 PD	ICR (2N)	Aggregation	42	8	0	0	8	100.0	2/2(100%)
B6: Targeted (#48)	16	+/−	L1000+BIO	ICR(2N)	Aggregation	60	4	0	0	4	100.0	2/2(100%)
B6	B6: Targeted (#C5)	+/−	L1000 + BIO	ICR(2N)	Aggregation	120	2	1	0	1	50.0	n.d.
B6: Targeted (#F1)	16	+/−	L1000+BIO	ICR(2N)	Aggregation	84	6	0	0	6	100.0	2/2(100%)
B6: XO (#2C7)	16	+/+	L1000 + BIO + PD	ICR (2N)	Aggregation	80	6	1	0	5	83.3	2/2(100%)
B6: XO (#2G3)	16	+/+	L1000 + BIO + PD	ICR (2N)	Aggregation	105	4	0	0	4	100.0	n.d.
V6.5: Targeted (#16–16)	27	−/−	Serum + Feeder cells + L1000	ICR (4N)	Aggregation	90	4	0	0	4	100.0	n.d.
V6.5: Targeted (#16–16)	30	−/−	L1000+BIO	ICR(2N)	Aggregation	60	5	1	1	18	94.7	2/2 (100%)
B6: Targeted (#B14)	22	−/−	L1000+BIO	ICR(2N)	Aggregation	60	5	1	1	3	60.0	n.d.

To examine whether GSK3 inhibitor can support B6 ES self-renewal without spontaneous differentiation, we checked colony morphology and alkaline phosphatase activity in the following cultures: 10 U/ml LIF, 1000 U/ ml LIF, 10 U/ml LIF plus BIO, and 1000 U/ml LIF plus BIO. After 5 days, in the 1000 U/ml LIF plus BIO culture, ES colonies showed round shapes and strong activity of alkaline phosphatase under the serum- and feeder-free condition (Fig. 1b). These round-shaped colonies resembled ES colonies that were cultured on a feeder layer. On the other hand, in the 10 U/ml LIF plus BIO culture, the number of partially differentiated colonies gradually increased according to passage number similar to the 1000 U/ml LIF single culture. Moreover, we examined the effects of BIO on cell proliferation (Fig. 1c). The growth speed in BIO-treated cultures was significantly faster than that in BIO-absent cultures, and also LIF addition enhanced ES cell proliferation. Taken together, these results indicate that in combination with LIF, GSK3 inhibitor enhances self-renewal, the suppression of spontaneous differentiation, and cell proliferation even in the serum- and feeder-free culture.

Next, we investigated a pluripotency marker in the serum- and feeder-free B6 ES cells (Fig. 1d). To examine the activity of Oct4 (also known as Pou5f1) promoter containing distal and proximal enhancers, we established Oct4-GIN (Oct4 promoter-GFP-IRES-Neo) ES cells, in which both GFP and neomycin-resistance gene (Neo) were transcribed by the Oct4 promoter. After the selection culture with G418, GFP expression was clearly observed in all living ES colonies stimulated with 1000 U/ml LIF plus BIO (Fig. 1d). However, partial expression of GFP was found in 1000 U/ml LIF single, 10 U/ml LIF plus BIO, and 10 U/ml LIF single conditions, respectively. These observations suggest that in concert with LIF/STAT3 signaling, GSK3 inhibition maintains the serum- and feeder-free B6 ES cells in a pluripotent state.

### Expression of Self-Renewal and Pluripotency Makers was Upregulated by BIO Treatment in Serum- and Feeder-Free B6 ES Cells

**FIG. 2:**
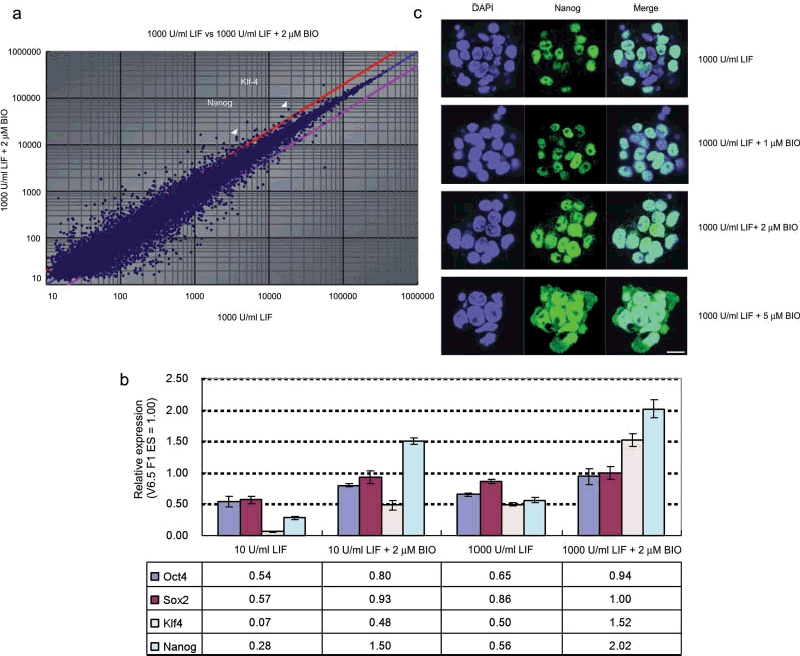
Upregulation of self-renewal and pluripotency markers in serum- and feeder-free B6 ES cells stimulated by BIO treatment. (a) Scatter plots showing a comparison of global gene expression between 1000 U/ml LIF alone and 1000 U/ml LIF plus 2 μM BIO culture conditions, as determined by DNA microarrays (*n* = 3). Lines indicate the linear equivalent and two-fold changes in gene expression levels between the samples. (b) Quantitative RT-PCR analyses. Error bars are standard deviations (*n* = 4). (c) Immunofluorescence analyses of the serum- and feeder-free B6 ES cells stained for Nanog and DAPI (nuclei). Scale bar: 50 μm.

To examine how GSK3 inhibitor enhanced self-renewal and pluripotency in the serum- and feeder-free B6 ES cells cultured with LIF (1000 U/ml) alone, we performed DNA microarray analyses (Fig. 2a). In this study, we identified 364 upregulated and 788 downregulated genes (over two-fold) in response to BIO (Tables S1 and S2). Gene Ontology analysis suggested that BIO functions as a general transcriptional repressor (*P*-value = 1.31 × 10^−8^; Tables S3 and S4). Upon BIO addition, expression levels of Wnt target genes such as Brachyury (T) and Cdx1 significantly increased, demonstrating that Wnt/β-catenin signaling was activated as shown in Figure S1. In the ES cells supported by LIF plus BIO, Nanog and Klf family members (Klf2, Klf4, and Klf5) of pluripotency markers were approximately three-fold upregulated (Fig. 2a,b). Interestingly, immunocytochemistry revealed that Nanog signals increased by BIO treatment in a dose-dependent manner and were also detected not only in the nucleus but also in the cytoplasm with 5 μM of BIO treatment (Fig. 2c). In addition, we observed that BIO stimulation converted the cells from heterogeneous to homogeneous expression of Nanog, while LIF single treatment showed heterogeneity as in previous reports (Chambers *et al*., 2007; Singh *et al*., 2007) (Fig. 2c). Expression levels of Oct4 and Sox2 also slightly increased to the same level as those in B6/129 F1 hybrid ES (V6.5) cells cultured with serum and feeder layers (Fig. 2b). For quantitative RT-PCR analysis, F1 hybrid ES cells were used as a standard control because of their high developmental potency to contribute to founder mice (19 100% ES-derived coat-color mice/270 embryos transferred, 7.0%; Table 1). Besides the core stemness genes, BIO treatment significantly increased expression levels of other self-renewal regulators (Tables S1 and S2). Upon BIO treatment, expression levels of BMP4, Id1, and Id3 were approximately four-fold upregulated, suggesting that autocrine BMP4 activates Id1 and Id3 expression through the activation of Smad signaling, which is involved in the inhibition of differentiation to neuroectoderm (Qi *et al*., 2004; Ying *et al*., 2003). In addition, BIO treatment also significantly increased the expressions of Tbx3, Esrrb, and Tcl1, which have been newly identified as self-renewal regulators by the method of short hairpin RNA (shRNA) loss-of-function (Ivanova *et al*., 2006). The overexpressions of Tbx3, Esrrb, and Tcl1 in BIO-treated ES cells might suppress ES cell differentiation into neuroectoderm, mesoderm, and endoderm as in a previous report (Ivanova *et al*., 2006). In contrast, expression levels of FGF5, a marker for primitive ectoderm cells, were 10-fold downregulated, suggesting that BIO-treated ES cells are resistant to differentiation stimuli. Consistent with this suggestion, even in the LIF-absent condition, BIO-treated ES cells had alkaline phosphatase activity and maintained Oct4 and Nanog expression (Fig. S2). Taken together, these results suggest that GSK3 inhibition by BIO efficiently enhance self-renewal and pluripotency by the upregulation of stemness genes, which form a robust autoregulatory circuit to maintain ES cells in a self-renewing pluripotent state.

### Germline-Competent ES-Derived Founder Mice were Stably Generated from Serum- and Feeder-Free ES Cells in LIF Plus BIO Medium

**FIG. 3:**
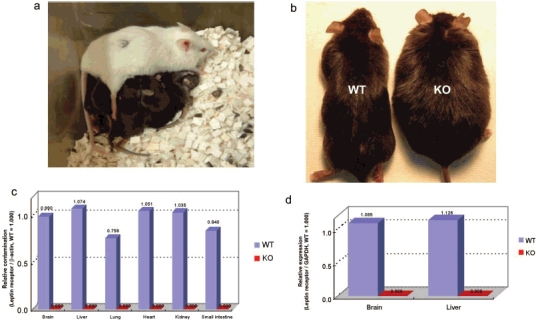
Serum- and feeder-free B6 ES cells in LIF plus BIO have the ability to differentiate into the whole body. WT (+/+) (a and b left side) and leptin receptor KO (−/−) (b right side) mice on B6 background were directly generated from WTand leptin receptor KO ES cells in 1000 U/ml LIF plus 2 μM BIO by aggregating with each eight-cell-stage diploid embryo. ICR mice were used as recipients for embryo transplantation. By using genomic DNA (c) and total RNA (d) of the founder mice, contamination of host ICR cells were examined with quantitative PCR of the leptin receptor.

Because BIO treatment enhanced self-renewal and pluripotency markers in serum- and feeder-free B6 ES cells in vitro, we tested their developmental potential by generating founder mice (Table 1). To investigate whether the production rate of germline-competent founder mice is improved by using B6 ES cells stimulated with 1000 U/ml LIF plus BIO, we used the aggregation method with one eight-cell-stage diploid embryo. At the beginning of this study, we traced the fate of GFP expressing B6 ES cells after aggregation. Unexpectedly, a fluorescent image of the blastocyst showed that GFP in B6 ES cells was entirely, but not partially, expressed in the ICM (Fig. S3). This observation indicates that serumand feeder-free B6 ES cells cultured in LIF plus BIO had full potential to contribute to the embryo proper. Actually, almost all founder mice obtained by the diploid aggregation method were 100% ES-derived black coatcolor, developed to adults without abnormality, and exhibited full germline transmission as well as mice obtained by the tetraploid complementation method (11 100% ES-derived coat-color mice/12 live offsprings, 91.7%; Fig. 3a and Table 1). Similar results were obtained, when ES cells stimulated with 1000 U/ml LIF plus BIO were injected into the perivitelline space of each ICR eight-cell-stage diploid embryo by conventional or laser-assisted method (Table 1). On the other hand, when the ES cells cultured in either 1000 U/ml LIF alone or 10 U/ml LIF plus BIO were aggregated with host diploid embryos, the rates of 100% black coat-color in live offsprings were ∼20–30% (Table 1). In the case of 10 U/ ml LIF-treated ES cells, all founder mice were entirely derived from host ICR embryos (Table 1). These results show that serum- and feeder-free B6 ES cells in 1000 U/ ml LIF plus BIO combination cultures have high developmental potency to contribute to founder mice.

The inhibition of the MEK pathway through the FGF receptor was reported to suppress lineage commitment even in the LIF absent condition (Burdon *et al*., 1999; Hamazaki *et al*., 2006; Kunath *et al*., 2007), suggesting that MEK inhibition promotes developmental potential of ES cells. However, in our study, PD98059, which is a known MEK inhibitor, did neither enhance the production rate of 100% ES-derived founder mice nor change the morphology of ES cells (Table 1 and Fig. S4). Therefore, it appears that MEK inhibition is not required for promoting the developmental potency of serum- and feeder-free B6 ES cells.

ES-derived coat-color founder mice (100%) were also efficiently generated from B6/129 F1 hybrid ES (V6.5) cells under the serum- and feeder-free condition (8 100% ES-derived coat-color mice/42 embryos transferred, 23.8%; Table 1 and Fig. S5). In our experiments, serumand feeder-free F1 hybrid ES cells in 1000 U/ml LIF plus BIO medium exhibited round morphology and full developmental potential by the diploid aggregation method, the same as inbred B6 ES cells. These results suggest that regardless of the genetic background of the mouse strain, a synergistic effect by LIF plus BIO treatment is effective for enhancing the developmental potential in various ES cells.

Next, we tested whether heterozygous mice were directly obtained from gene-targeted B6 ES clones (Table 1). After electroporation of gene-targeting vectors, heterozygous (+/−) clones were screened in the 1000 U/ml LIF plus BIO medium under the serum- and feeder-free condition. By aggregating those clones with each diploid embryo, almost all heterozygous pups were stably born with 100% black coat-color, and then exhibited full germline transmission (Table 1). However, some clones showed a loss of developmental potency due to abnormal karyotypes (data not shown). Thus, serum- and feeder-free ES cells cultured in 1000 U/ml LIF plus BIO were not only able to generate heterozygous mice, but also robust against genetic manipulation.

In addition, we successfully generated female B6 founder mice by the aggregation of female (38 + XO) B6 ES cells with ICR eight-cell-stage diploid embryos. These female mice with 100% black coat-color were fertile (Table 1) and also available for in vitro fertilization (data not shown).

Finally, to confirm that 100% black coat-color founder mice were fully derived from B6 ES cells treated with LIF plus BIO, we assessed the contamination of host ICR cells by quantitative PCR. For this analysis, we established homozygotes (−/−) of leptin receptor targeting in B6 ES cells cultured in 1000 U/ml LIF plus BIO under the serum- and feeder-free condition (Fig. S6), and then tested whether KO (−/−) founder mice were directly generated from the KO (−/−) ES cells without the contamination of host wild-type (+/+) cells. By aggregating KO ES cells with one eight-cell-stage diploid embryo, founder mice were born normally and developed to adults with 100% black coat-color. Expectedly, the KO ES-derived mice showed fatty bodies as same as db/db mice (Fig. 3b). Quantitative PCR of the KO ES-derived mice showed that Exon 1 of the leptin receptor was completely deleted in the genomes of diverse tissues (brain, liver, lung, heart, kidney, and small intestine) (Fig. 3c). Consistent with these results, its transcription was not detected in the brain and liver at all (Fig. 3d), indicating that the founder mice generated from KO ES cells were identical to KO mice. Taken together, we concluded that serum- and feeder-free ES cells cultured in LIF and BIO had a high capacity to differentiate into the whole body. In addition, the direct generation of KO ES-derived mice facilitated speedy phenotypic analyses in vivo.

## DISCUSSION

The treatment of LIF plus BIO dramatically changed the morphology of serum- and feeder-free B6 ES cells and significantly increased the expressions of self-renewal and pluripotency regulators. The aggregation or microinjection of serum- and feeder-free B6 ES cells cultured in LIF plus BIO to each eight-cell-stage diploid embryo stably provided germline-competent B6 ES-derived mice. Previously, it was reported that laser-assisted microinjection of ES cells into an eight-cell-stage diploid embryo efficiently provides founder generation mice that are available for immediate phenotypic analyses (Poueymirou *et al*., 2007). Certainly, the eight-cell-stage embryo method is useful for obtaining ES-derived mice compared to the conventional blastocyst injection method. However, judging from the data in Table 1, the birth rate of ES-derived mice seems to be highly dependent on the developmental potential of ES cells regardless of embryo manipulation. Accordingly, we hereby propose that serum- and feeder-free ES cells cultured with LIF plus GSK3 inhibitor are valuable for generating germline-competent ES-derived mice.

It is accepted that B6 ES cells have low potency to colonize germ cells in their founder mice. In ES cell culture, Nanog expression is thought to be an important factor for the maintenance of germline competency. Compared with LIF single-treated B6 ES cells, LIF plus BIO-treated B6 ES cells showed homogeneous expression pattern of Nanog (Fig. 2c). By aggregating them with each eight-cell-stage diploid embryo, they stably differentiated into germ cells in their founder mice, which exhibited full germline transmission (Table 1). It has been reported that Nanog is dispensable for expression of somatic pluripotency but is specifically required for formation of germ cells (Chambers *et al*., 2007). Moreover, by the selection of Nanog expression, the germline-competent induced pluripotent stem (iPS) cells have been successfully established from embryonic fibroblasts (Okita *et al*., 2007). From these findings, we speculate that homogeneous expression of Nanog is a landmark in ES cells and/or iPS cells that have high potential to differentiate particularly into germ cells.

ES cells have been reported to be maintained by three inhibitors (3i) for FGF receptor and GSK3 (Ying *et al*., 2008). For GSK3 inhibitors, BIO and CHIR99021 were used in our study and that of Ying and colleagues, respectively. After each treatment of GSK3 inhibitor, the expression levels of Wnt/β-catenin target genes such as Brachyury (T) and Cdx1 were similarly upregulated. However, BIO treatment significantly increased the expression levels of Tbx3, Esrrb, and Tcl1 in B6-derived ES cells, but CHIR99021 did not have the same upregulation effect on these genes in 129-derived ES cells. Moreover, In addition to GSK3 inhibitor, we have used LIF in KSR-based medium, while Ying's group used two inhibitors for the FGF receptor instead of LIF in N2B27-based medium. In our studies, the LIF plus BIO combination culture enhanced cell growth and developmental potential of serum- and feeder-free B6 ES cells as shown in Figure 1c and Table 1. Thus, different culture conditions derived from different combinations of chemical compounds, culture media, and ES cell strains may explain these different findings. From the standpoint of stable generation of founder mice, it is interesting for us to examine which conditions bring out the best developmental potential in each ES cell line, although GSK3 inhibition is crucial for ES cell culture.

Finally, diploid embryo aggregation with the serumand feeder-free B6 ES cells cultured in LIF plus GSK3 inhibitor is a rapid, cost-effective, and easy-to-learn method for generating germline-competent B6 ES-derived founder mice. Furthermore, GSK3 inhibition together with LIF/STAT3 signaling makes it possible to enhance the developmental potential of various ES cells without specific skills and experience, because the serum lot check and the preparation for feeder layers are skipped. Generally, in the conventional method, 129 or F1 hybrid ES cells cultured in the presence of serum and feeder layers have been used for the production of germline-competent chimeras. Partial chimeric mice generated from these ES cells require multiple rounds of breeding to produce homozygous mutations within a given mouse. The desired mice are produced with a mixed genetic background at low frequencies in the final breeding step. However, the use of B6 ES cell line instead of 129 or F1 hybrid ES cell line eliminates the need for backcross breeding. Founder mice entirely derived from female XO, heterozygous, or homozygous mutant B6 ES cells were directly available for intercross breeding and phenotypic analysis in vivo. In addition, the access to the public C57BL/6J mouse sequence and the RPCI23 C57BL/6J BAC genomic library is a powerful tool that facilitates the production of gene-targeting vectors for the manipulation of genes in C57/BL6 ES cell lines. Therefore, from the point of view of saving time and labor, the established serum- and feeder-free B6 ES cells will be helpful and useful for large-scale functional genomic analyses with genetically engineered mice on B6 background.

## MATERIALS AND METHODS

### ES Cell Culture

C57/BL6J ES cells (TB6-2) were originally established by a previously published procedure (Shimizukawa *et al*., 2005). By karyotyping, more than 90% of TB6-2 cells showed normal chromosome numbers (38 + XY). For serum-free cultures, we used Dulbecco's Minimal Essential Medium (DMEM) (Invitrogen) supplemented with 25 mM D-glucose, 0.1 mM nonessential amino acids, 1 mM sodium pyruvate, 0.1 mM 2 mercaptoethanol, 4 mM L-glutamine, 100 U/ml penicillin, 100 lg/ml streptomycin, 1000 U/ml LIF, and 15% KSR (Invitrogen). Feeder-free ES cells were established from TB6-2 cells cultured on MEF (Mouse Embryonic Fibroblast) cells. To establish the feeder-free ES cells, the feeder-dependent TB6-2 cells were cultured on a gelatin-coated plate in the presence of LIF. After two passages under feeder-free conditions, MEF cells were completely removed, and the expanded ES cells were stocked. For inhibition of GSK3 and/or MEK activities, we used 2 μM of BIO (6-bromoindirubin-3′-oxime) (Calbiochem) and/or 40 μM of PD98059 (Calbiochem). The alkaline phosphatase assays in ES cells were preformed by an alkaline phosphatase detection kit (Chemicon). Cell growth in ES cells was examined by a CellTitre-GloR Luminescent Cell Viability assay (Promega). Female karyotype (XO) ES cells were cloned by screening for Y chromosome deletion in XY ES cells. The targeting in B6 ES cells was performed under the feeder- and serum-free condition. Heterozygous ES cells containing an attenuated neomycin phosphotransferase gene (resistance to G418) under the control of the mouse phosphoglycerol kinase gene (PGK) promoter were selected in 0.25 mg/ml of G418. After 9 days of selection culture, we picked up colonies and screened heterozygous ES clones by PCR and Southern blot analyses. Homozygous ES cells were obtained from heterozygous ES cells by the selection culture at 1 mg/ ml (four times higher concentration) of G418 concentration.

### DNA Construction

For Oct4-GIN vector construction, the Oct4 promoter containing distal and proximal enhancer regions was amplified from mouse genomic DNA by PCR. Primers were as follows: forward, 5′-ttctgtcgactctaggcacgcttaggc-3′ and reverse, 5′-ttctagatctccgagccgggggcctggtgg-3′. The Oct4 promoter was inserted into a vector consisting of a GFP-IRES-Neo.

### Gene Expression Analysis

Total RNA was extracted by RNeasy kits (Qiagen). The first strand cDNA synthesis by reverse transcription was performed with 0.25 μg of total RNA at 37°C for 2 h using a high capacity cDNA archive kit (Applied Biosystems) and subsequently diluted 10 times. Endogenous mRNA levels of pluripotency markers (Oct4, Sox2, Nanog, and Klf4) were measured with inventoried TaqMan probes using an Applied Biosystems 7900HT (Applied Biosystems). PCR primers and TaqMan probes (5′-FAM-3′-Black hole Quencher, Biosearch); 5′-FAM- or 5′-VIC-Minor Groove Binder Non-Fluorescent Quencher (MGBNFQ; Applied Biosystems) were used as follows: leptin receptor forward, 5′-cggagaccacgcaactt-3′; leptin receptor reverse, 5′-ccccgggcagtttcca-3′; leptin receptor probe, 5′-FAM-caggcctctgactact-MGBNFQ-3′; β actin forward, 5′-aggtcatcactattggcaacga-3′; β actin reverse, 5′-cacaggattccatacccaagaag-3′; β actin probe, 5′-VIC-atgccctgaggctcttttccagcctt-MGBNFQ-3′. GAPDH expression was examined by using Rodent GAPDH control regents (Applied Biosystems). All results were normalized to β actin or GAPDH expression. DNA microarrays (Agilent) were performed with total RNAs from ES cells treated with or without 2 μM of BIO for five days.

### Immunofluorescence Microscopy

ES cells were fixed with 4% paraformaldehyde/PBS for 30 min at room temperature and permeabilized with 0.1% Triton X-100/PBS at room temperature for 30 min. Primary and secondary antibodies were diluted to final working concentrations in 1% BSA, 0.1% TritonX-100/ PBS. Specimens were mounted with ProLong gold anti-fade reagent containing DAPI (Molecular Probe). Images of immunofluorescent samples were obtained with a Zeiss LSM510 confocal microscope. The final concentrations of antibodies were as follows: anti-β catenin-FITC, 1:250 (BD Bioscience); anti-Nanog, 1:250 (ReproCELL, Tokyo, Japan); goat anti-rabbit IgG-FITC, 1/100 (Invitrogen).

### Generation of Founder Mice

Two-cell-stage embryos were collected from oviducts of plugged females at 1.5 dpc. After overnight incubation of the embryos at 37°C/5% CO_2_ in Hepes-buffered potassium simplex optimized medium (KSOM), zona pellucidae of eight-cell-stage embryos were removed with an acid-tyrode solution. The diploid embryos were transferred into each KSOM drop. For aggregation, a number of clumps of loosely connected ES cells were selected under a dissecting microscope, and then one of the ES cell clumps was put on the side of one embryo. After overnight incubation at 37°C/5% CO_2_ in KSOM, the aggregated embryos were developed to the late morula/ early blastocyst stage, and then transplanted into the uterine horns of pseudo-pregnant ICR mice by the standard method. Conventional or laser-assisted microinjection was performed according to a previous report (Poueymirou *et al*., 2007). A standard injection needle was used to introduce 7–9 ES cells through a perforation and into the perivitelline space. In laser-assisted microinjection, a XYClone laser system (Hamilton) was used to make a perforation in the zona pellucida. The injected eight-cell-stage embryos were cultured overnight at 37°C/5% CO_2_ in KSOM before embryo transplantation. All animal procedures were carried out according to the Merck Worldwide Policy on Animal Care and Use.

## References

[b1] Auerbach W, Dunmore JH, Fairchild-Huntress V, Fang Q, Auerbach AB, Huszar D, Joyner AL (2000). Establishment and chimera analysis of 129/SvEv- and C57BL/6-derived mouse embryonic stem cell lines. Biotechniques.

[b2] Burdon T, Stracey C, Chambers I, Nichols J, Smith A (1999). Suppression of SHP-2 and ERK signalling promotes self-renewal of mouse embryonic stem cells. Dev Biol.

[b3] Capecchi MR (2001). Generating mice with targeted mutations. Nat Med.

[b4] Cartwright P, McLean C, Sheppard A, Rivett D, Jones K, Dalton S (2005). LIF/STAT3 controls ES cell self-renewal and pluripotency by a Myc-dependent mechanism. Development.

[b5] Chambers I, Silva J, Colby D, Nichols J, Nijmeijer B, Robertson M, Vrana J, Jones K, Grotewold L, Smith A (2007). Nanog safeguards pluripotency and mediates germline development. Nature.

[b6] Doble BW, Patel S, Wood GA, Kockeritz LK, Woodgett JR (2007). Functional redundancy of GSK-3alpha and GSK-3beta in Wnt/beta-catenin signaling shown by using an allelic series of embryonic stem cell lines. Dev Cell.

[b7] Eggan K, Akutsu H, Loring J, Jackson-Grusby L, Klemm M, Rideout WM, Yanagimachi R, Jaenisch R (2001). Hybrid vigor, fetal overgrowth, and viability of mice derived by nuclear cloning and tetraploid embryo complementation. Proc Natl Acad Sci USA.

[b8] Evans MJ (2001). The cultural mouse. Nat Med.

[b9] Goldstein JL (2001). Laskers for 2001: Knockout mice and test-tube babies. Nat Med.

[b10] Hamazaki T, Kehoe SM, Nakano T, Terada N (2006). The Grb2/Mek pathway represses Nanog in murine embryonic stem cells. Mol Cell Biol.

[b11] Hoeflich KP, Luo J, Rubie EA, Tsao MS, Jin O, Woodgett JR (2000). Requirement for glycogen synthase kinase-3beta in cell survival and NF-kappaB activation. Nature.

[b12] Ivanova N, Dobrin R, Lu R, Kotenko I, Levorse J, DeCoste C, Schafer X, Lun Y, Lemischka IR (2006). Dissecting self-renewal in stem cells with RNA interference. Nature.

[b13] Kunath T, Saba-El-Leil MK, Almousailleakh M, Wray J, Meloche S, Smith A (2007). FGF stimulation of the Erk1/2 signalling cascade triggers transition of pluripotent embryonic stem cells from self-renewal to lineage commitment. Development.

[b14] Matsuda T, Nakamura T, Nakao K, Arai T, Katsuki M, Heike T, Yokota T (1999). STAT3 activation is sufficient to maintain an undifferentiated state of mouse embryonic stem cells. EMBO J.

[b15] Nagy A, Rossant J, Nagy R, Abramow-Newerly W, Roder JC (1993). Derivation of completely cell culture-derived mice from early-passage embryonic stem cells. Proc Natl Acad Sci USA.

[b16] Niwa H, Burdon T, Chambers I, Smith A (1998). Self-renewal of pluripotent embryonic stem cells is mediated via activation of STAT3. Genes Dev.

[b17] Okita K, Ichisaka T, Yamanaka S (2007). Generation of germline-competent induced pluripotent stem cells. Nature.

[b18] Poueymirou WT, Auerbach W, Frendewey D, Hickey JF, Escaravage JM, Esau L, Dore AT, Stevens S, Adams NC, Dominguez MG, Gale NW, Yancopoulos GD, DeChiara TM, Valenzuela DM (2007). F0 generation mice fully derived from gene-targeted embryonic stem cells allowing immediate phenotypic analyses. Nat Biotechnol.

[b19] Qi X, Li TG, Hao J, Hu J, Wang J, Simmons H, Miura S, Mishina Y, Zhao GQ (2004). BMP4 supports self-renewal of embryonic stem cells by inhibiting mitogen-activated protein kinase pathways. Proc Natl Acad Sci USA.

[b20] Sato N, Meijer L, Skaltsounis L, Greengard P, Brivanlou AH (2004). Maintenance of pluripotency in human and mouse embryonic stem cells through activation of Wnt signaling by a pharmacological GSK-3-specific inhibitor. Nat Med.

[b21] Shimizukawa R, Sakata A, Hirose M, Takahashi A, Iseki H, Liu Y, Kunita S, Sugiyama F, Yagami K (2005). Establishment of a new embryonic stem cell line derived from C57BL/6 mouse expressing EGFP ubiquitously. Genesis.

[b22] Singh AM, Hamazaki T, Hankowski KE, Terada N (2007). A heterogeneous expression pattern for Nanog in embryonic stem cells. Stem Cells.

[b23] Singla DK, Schneider DJ, LeWinter MM, Sobel BE (2006). wnt3a but not wnt11 supports self-renewal of embryonic stem cells. Biochem Biophys Res Commun.

[b24] Smithies O (2001). Forty years with homologous recombination. Nat Med.

[b25] Toyooka Y, Shimosato D, Murakami K, Takahashi K, Niwa H (2008). Identification and characterization of subpopulations in undifferentiated ES cell culture. Development.

[b26] Ying QL, Nichols J, Chambers I, Smith A (2003). BMP induction of Id proteins suppresses differentiation and sustains embryonic stem cell self-renewal in collaboration with STAT3. Cell.

[b27] Ying QL, Wray J, Nichols J, Batlle-Morera L, Doble B, Woodgett J, Cohen P, Smith A (2008). The ground state of embryonic stem cell self-renewal. Nature.

