# Supplementary material for: Stable generation of serum- and feeder-free embryonic stem cell-derived mice with full germline-competency by using a GSK3 specific inhibitor
Source: Genesis. 2009 Apr 23;47(6):414–22. doi: 10.1002/dvg.20514 (PMC2726955; doi:10.1002/dvg.20514)
Supplement: Supplementary file 7 [file dvg0047-0414-SD7.doc]

**Supplementary Table 1**

| **Entrez Gene ID** | **Gene Symbol** | **Gene Name** | **1000 U/ml LIF + 2 μM BIO** | **1000 U/ml LIF** | **Ratio(1000 U/ml LIF + 2 μM BIO / 1000 U/ml LIF)** |
| --- | --- | --- | --- | --- | --- |
| 21946 | Pglyrp1 | peptidoglycan recognition protein 1 | 686.7 | 14.8 | 46.42 |
| 12590 | Cdx1 | caudal type homeo box 1 | 1796.6 | 56.2 | 31.98 |
| 18208 | Ntn1 | netrin 1 | 477.0 | 34.8 | 13.58 |
| 21386 | Tbx3 | T-box 3 | 16031.0 | 1221.2 | 13.12 |
| 12182 | Bst1 | bone marrow stromal cell antigen 1 | 192.9 | 15.7 | 12.26 |
| 24108 | Ubd | ubiquitin D | 2168.0 | 178.5 | 12.14 |
| 20231 | Nkx1-2 | NK1 transcription factor related, locus 2 (Drosophila) | 241.2 | 20.1 | 11.96 |
| 75013 | 4930502E18Rik | RIKEN cDNA 4930502E18 gene | 1218.6 | 106.4 | 11.45 |
| 64406 | Sp5 | trans-acting transcription factor 5 | 2444.1 | 217.6 | 11.23 |
| 94179 | Krt23 | keratin 23 | 250.5 | 22.9 | 10.93 |
| 232441 | Rerg | RAS-like, estrogen-regulated, growth-inhibitor | 242.4 | 23.5 | 10.33 |
| 71939 | Apol6 | apolipoprotein L, 6 | 636.6 | 63.9 | 9.96 |
| 14859 | Gsta3 | glutathione S-transferase, alpha 3 | 1078.5 | 117.5 | 9.18 |
| 81879 | Tcfcp2l1 | transcription factor CP2-like 1 | 7099.9 | 829.1 | 8.53 |
| 13813 | Eomes | eomesodermin homolog (Xenopus laevis) | 709.8 | 85.0 | 8.36 |
| 330721 | Nek5 | NIMA (never in mitosis gene a)-related expressed kinase 5 | 127.4 | 16.1 | 7.93 |
| 246730 | Oas1a | 2'-5' oligoadenylate synthetase 1A | 839.0 | 109.7 | 7.64 |
| 22634 | Plagl1 | pleiomorphic adenoma gene-like 1 | 3104.4 | 407.7 | 7.61 |
| 15229 | Foxd1 | forkhead box D1 | 123.7 | 17.7 | 6.99 |
| 72381 | 2210409E12Rik | RIKEN cDNA 2210409E12 gene | 13763.6 | 1985.6 | 6.94 |
| 66222 | Serpinb1a | serine (or cysteine) peptidase inhibitor, clade B, member 1a | 277.7 | 40.6 | 6.82 |
| 76797 | 2410137M14Rik | RIKEN cDNA 2410137M14 gene | 162.3 | 25.4 | 6.38 |
| 13417 | Dnahc8 | dynein, axonemal, heavy chain 8 | 352.3 | 57.2 | 6.17 |
| 70744 | 6330403L08Rik | RIKEN cDNA 6330403L08 gene | 145.7 | 23.7 | 6.15 |
| 209176 | C230043N17Rik | RIKEN cDNA C230043N17 gene | 242.2 | 40.1 | 6.04 |
| 252830 | Obox6 | oocyte specific homeobox 6 | 128.4 | 21.4 | 6.01 |
| 18590 | Pdgfa | platelet derived growth factor, alpha | 30780.5 | 5133.7 | 6.00 |
| 71827 | Lrrc34 | leucine rich repeat containing 34 | 2551.3 | 431.9 | 5.87 |
| 17702 | Msx2 | homeo box, msh-like 2 | 261.8 | 44.8 | 5.85 |
| 85031 | Pla1a | phospholipase A1 member A | 854.2 | 149.6 | 5.70 |
| 240332 | Slc6a7 | solute carrier family 6 (neurotransmitter transporter, L-proline), member 7 | 174.3 | 30.9 | 5.65 |
| 20689 | Sall3 | sal-like 3 (Drosophila) | 519.2 | 92.9 | 5.58 |
| 12159 | Bmp4 | bone morphogenetic protein 4 | 1572.1 | 285.9 | 5.50 |
| 15427 | Hoxc9 | homeo box C9 | 123.6 | 22.5 | 5.48 |
| 14745 | Edg2 | endothelial differentiation, lysophosphatidic acid G-protein-coupled receptor, 2 | 331.0 | 60.7 | 5.45 |
| 67374 | Jam2 | junction adhesion molecule 2 | 6818.4 | 1254.9 | 5.43 |
| 54204 | Sept1 | septin 1 | 785.6 | 145.1 | 5.41 |
| 232333 | Slc6a1 | solute carrier family 6 (neurotransmitter transporter, GABA), member 1 | 292.8 | 57.5 | 5.09 |
| 19224 | Ptgs1 | prostaglandin-endoperoxide synthase 1 | 122.6 | 24.4 | 5.00 |
| 73660 | Cabp4 | calcium binding protein 4 | 100.0 | 20.4 | 4.91 |
| 108017 | Fxyd4 | FXYD domain-containing ion transport regulator 4 | 105.1 | 21.8 | 4.81 |
| 545428 | 2610301F02Rik | RIKEN cDNA 2610301F02 gene | 363.7 | 77.2 | 4.67 |
| 76886 | 6430514L14Rik | RIKEN cDNA 6430514L14 gene | 332.6 | 71.9 | 4.63 |
| 16970 | Lrmp | lymphoid-restricted membrane protein | 1317.8 | 288.1 | 4.57 |
| 18442 | P2ry2 | purinergic receptor P2Y, G-protein coupled 2 | 1270.5 | 277.8 | 4.56 |
| 268973 | Card12 | caspase recruitment domain family, member 12 | 263.5 | 58.1 | 4.53 |
| 76527 | 2010004A03Rik | RIKEN cDNA 2010004A03 gene | 417.5 | 93.2 | 4.48 |
| 217122 | A430060F13Rik | RIKEN cDNA A430060F13 gene | 104.4 | 23.5 | 4.44 |
| 109820 | Pgc | progastricsin (pepsinogen C) | 68.3 | 15.4 | 4.44 |
| 30785 | Cttnbp2 | cortactin binding protein 2 | 66.0 | 15.1 | 4.36 |
| 76306 | 1110021L09Rik | RIKEN cDNA 1110021L09 gene | 721.8 | 167.7 | 4.30 |
| 22416 | Wnt3a | wingless-related MMTV integration site 3A | 156.0 | 36.4 | 4.28 |
| 70977 | 4931407G18Rik | RIKEN cDNA 4931407G18 gene | 222.9 | 52.3 | 4.25 |
| 434903 | CN716893 | expressed sequence CN716893 | 429.4 | 102.6 | 4.18 |
| 381411 | Gm1967 | gene model 1967, (NCBI) | 1223.1 | 297.3 | 4.11 |
| 243262 | Oas1f | 2'-5' oligoadenylate synthetase 1F | 566.6 | 133.4 | 4.08 |
| 12683 | Cidea | cell death-inducing DNA fragmentation factor, alpha subunit-like effector A | 1029.1 | 252.7 | 4.07 |
| 20997 | T | brachyury | 164.2 | 40.3 | 4.07 |
| 13850 | Ephx2 | epoxide hydrolase 2, cytoplasmic | 10323.7 | 2545.0 | 4.06 |
| 69820 | 1810059H22Rik | RIKEN cDNA 1810059H22 gene | 124.0 | 30.7 | 4.03 |
| 231293 | C130090K23Rik | RIKEN cDNA C130090K23 gene | 60.5 | 15.0 | 4.00 |
| 69698 | 2310046K01Rik | RIKEN cDNA 2310046K01 gene | 1795.5 | 451.7 | 3.98 |
| 230810 | Slc30a2 | solute carrier family 30 (zinc transporter), member 2 | 679.2 | 172.2 | 3.94 |
| 69134 | 2200001I15Rik | RIKEN cDNA 2200001I15 gene | 2166.5 | 552.1 | 3.93 |
| 71930 | 2310043M15Rik | RIKEN cDNA 2310043M15 gene | 1817.3 | 463.2 | 3.92 |
| 627821 | Av381130 | hypothetical protein LOC627821 | 419.6 | 107.2 | 3.91 |
| 22420 | Wnt6 | wingless-related MMTV integration site 6 | 305.5 | 78.1 | 3.90 |
| 18053 | Ngfr | nerve growth factor receptor (TNFR superfamily, member 16) | 5364.6 | 1382.0 | 3.88 |
| 17355 | Aff1 | AF4/FMR2 family, member 1 | 53.8 | 13.8 | 3.88 |
| 17907 | Mylpf | myosin light chain, phosphorylatable, fast skeletal muscle | 14254.0 | 3677.4 | 3.87 |
| 239102 | Zfhx2 | zinc finger homeobox 2 | 82.0 | 21.2 | 3.87 |
| 22419 | Wnt5b | wingless-related MMTV integration site 5B | 1026.0 | 266.7 | 3.85 |
| 70727 | Rasgef1a | RasGEF domain family, member 1A | 310.9 | 80.8 | 3.84 |
| 230073 | Ddx58 | DEAD (Asp-Glu-Ala-Asp) box polypeptide 58 | 1742.6 | 459.1 | 3.78 |
| 73375 | 1700057H21Rik | RIKEN cDNA 1700057H21 gene | 83.6 | 22.2 | 3.77 |
| 228765 | Sdcbp2 | syndecan binding protein (syntenin) 2 | 1001.2 | 268.6 | 3.72 |
| 71950 | Nanog | Nanog homeobox | 14014.4 | 3776.1 | 3.71 |
| 17288 | Mep1b | meprin 1 beta | 281.8 | 76.1 | 3.69 |
| 79455 | Pdcl2 | phosducin-like 2 | 211.2 | 58.1 | 3.63 |
| 103511 | BB146404 | expressed sequence BB146404 | 123.3 | 34.0 | 3.62 |
| 12808 | Cobl | cordon-bleu | 5484.9 | 1522.9 | 3.60 |
| 381371 | Gm1631 | gene model 1631, (NCBI) | 1451.4 | 404.4 | 3.58 |
| 20728 | Spic | Spi-C transcription factor (Spi-1/PU.1 related) | 429.9 | 120.3 | 3.57 |
| 21679 | Tead4 | TEA domain family member 4 | 488.9 | 138.4 | 3.53 |
| 20210 | Saa3 | serum amyloid A 3 | 317.2 | 91.4 | 3.47 |
| 18719 | Pip5k1a | phosphatidylinositol-4-phosphate 5-kinase, type 1 alpha | 530.0 | 153.2 | 3.46 |
| 17916 | Myo1f | myosin IF | 4720.6 | 1367.2 | 3.45 |
| 15901 | Id1 | inhibitor of DNA binding 1 | 996.7 | 287.1 | 3.43 |
| 17450 | Morc1 | microrchidia 1 | 5902.2 | 1735.2 | 3.40 |
| 71857 | 1700019H03Rik | RIKEN cDNA 1700019H03 gene | 1340.5 | 394.7 | 3.39 |
| 13829 | Epb4.9 | erythrocyte protein band 4.9 | 2536.9 | 754.1 | 3.36 |
| 219132 | D14Ertd668e | DNA segment, Chr 14, ERATO Doi 668, expressed | 1201.9 | 356.7 | 3.36 |
| 22341 | Vegfc | vascular endothelial growth factor C | 1600.3 | 477.7 | 3.35 |
| 26424 | Nr5a2 | nuclear receptor subfamily 5, group A, member 2 | 4008.1 | 1197.2 | 3.33 |
| 26380 | Esrrb | estrogen related receptor, beta | 12332.9 | 3706.9 | 3.32 |
| 16622 | Klk1b5 | kallikrein 1-related peptidase b5 | 156.0 | 46.9 | 3.32 |
| 228802 | BC018465 | cDNA sequence BC018465 | 82.5 | 24.9 | 3.30 |
| 100434 | Slc44a1 | solute carrier family 44, member 1 | 181.5 | 55.0 | 3.30 |
| 58865 | Tdh | L-threonine dehydrogenase | 185194.5 | 56196.6 | 3.30 |
| 219131 | Phf11 | PHD finger protein 11 | 470.3 | 138.3 | 3.29 |
| 16792 | Laptm5 | lysosomal-associated protein transmembrane 5 | 456.8 | 138.1 | 3.29 |
| 18028 | Nfib | nuclear factor I/B | 704.9 | 215.9 | 3.26 |
| 78625 | 1700061G19Rik | RIKEN cDNA 1700061G19 gene | 7946.8 | 2431.8 | 3.26 |
| 13032 | Ctsc | cathepsin C | 838.2 | 257.4 | 3.26 |
| 53627 | Porcn | porcupine homolog (Drosophila) | 5122.4 | 1574.3 | 3.25 |
| 21923 | Tnc | tenascin C | 49.2 | 15.2 | 3.22 |
| 93897 | Fzd10 | frizzled homolog 10 (Drosophila) | 2911.7 | 903.4 | 3.22 |
| 77583 | 5730593N15Rik | RIKEN cDNA 5730593N15 gene | 1134.2 | 352.5 | 3.22 |
| 21432 | Tcl1 | T-cell lymphoma breakpoint 1 | 1415.7 | 440.9 | 3.21 |
| 12591 | Cdx2 | caudal type homeo box 2 | 683.5 | 213.4 | 3.20 |
| 18158 | Nppb | natriuretic peptide precursor type B | 304.2 | 95.2 | 3.20 |
| 241230 | St8sia6 | ST8 alpha-N-acetyl-neuraminide alpha-2,8-sialyltransferase 6 | 131.7 | 41.3 | 3.18 |
| 217721 | BC011209 | cDNA sequence BC011209 | 422.4 | 133.7 | 3.16 |
| 12494 | Cd38 | CD38 antigen | 212.2 | 67.2 | 3.15 |
| 209837 | Slc38a5 | solute carrier family 38, member 5 | 1086.0 | 345.6 | 3.14 |
| 52055 | Rab11fip5 | RAB11 family interacting protein 5 (class I) | 1174.4 | 385.4 | 3.04 |
| 18020 | Nfatc2ip | nuclear factor of activated T-cells, cytoplasmic, calcineurin-dependent 2 interacting protein | 19902.7 | 6561.9 | 3.03 |
| 76477 | Pcolce2 | procollagen C-endopeptidase enhancer 2 | 3070.2 | 1015.9 | 3.02 |
| 15903 | Id3 | inhibitor of DNA binding 3 | 4118.7 | 1372.5 | 3.00 |
| 13614 | Edn1 | endothelin 1 | 82.5 | 27.3 | 3.00 |
| 12505 | Cd44 | CD44 antigen | 240.0 | 80.4 | 2.98 |
| 76380 | Ccdc46 | coiled-coil domain containing 46 | 148.9 | 50.0 | 2.97 |
| 70788 | 4631423F02Rik | RIKEN cDNA 4631423F02 gene | 200.6 | 67.9 | 2.96 |
| 16600 | Klf4 | Kruppel-like factor 4 (gut) | 55197.2 | 18734.6 | 2.95 |
| 110173 | Manba | mannosidase, beta A, lysosomal | 20313.7 | 6906.6 | 2.94 |
| 109700 | Itga1 | integrin alpha 1 | 65.2 | 22.3 | 2.93 |
| 56788 | Scube2 | signal peptide, CUB domain, EGF-like 2 | 97.6 | 33.1 | 2.92 |
| 109904 | Mcf2 | mcf.2 transforming sequence | 118.5 | 40.8 | 2.90 |
| 74516 | 8430417A20Rik | RIKEN cDNA 8430417A20 gene | 61.7 | 21.4 | 2.88 |
| 107585 | Dio3 | deiodinase, iodothyronine type III | 179.8 | 62.5 | 2.88 |
| 74186 | Ccdc3 | coiled-coil domain containing 3 | 136.9 | 47.6 | 2.88 |
| 75040 | 4930504H06Rik | RIKEN cDNA 4930504H06 gene | 218.6 | 76.1 | 2.87 |
| 242667 | Dlgap3 | discs, large (Drosophila) homolog-associated protein 3 | 5031.1 | 1756.2 | 2.86 |
| 245671 | Klf8 | Kruppel-like factor 8 | 1586.3 | 552.1 | 2.86 |
| 20196 | S100a13 | S100 calcium binding protein A13 | 815.3 | 284.8 | 2.86 |
| 75514 | 1700013H16Rik | RIKEN cDNA 1700013H16 gene | 47.8 | 16.6 | 2.86 |
| 71862 | Gpr160 | G protein-coupled receptor 160 | 1273.1 | 446.4 | 2.85 |
| 228550 | Itpka | inositol 1,4,5-trisphosphate 3-kinase A | 4777.3 | 1678.5 | 2.84 |
| 328443 | E130202H07Rik | RIKEN cDNA E130202H07 gene | 377.2 | 132.7 | 2.84 |
| 12053 | Bcl6 | B-cell leukemia/lymphoma 6 | 574.9 | 202.6 | 2.84 |
| 12640 | Cga | glycoprotein hormones, alpha subunit | 92.4 | 32.7 | 2.82 |
| 170765 | Dscr6 | Down syndrome critical region homolog 6 (human) | 137.3 | 48.9 | 2.81 |
| 17898 | Myl7 | myosin, light polypeptide 7, regulatory | 1931.6 | 688.6 | 2.81 |
| 14598 | Ggt1 | gamma-glutamyltransferase 1 | 246.1 | 87.7 | 2.80 |
| 29820 | Tnfrsf19 | tumor necrosis factor receptor superfamily, member 19 | 1993.8 | 711.2 | 2.80 |
| 327959 | Fbxo39 | F-box protein 39 | 68.3 | 24.5 | 2.80 |
| 320214 | 4932425I24Rik | RIKEN cDNA 4932425I24 gene | 98.6 | 35.1 | 2.79 |
| 11614 | Nr0b1 | nuclear receptor subfamily 0, group B, member 1 | 7750.3 | 2773.7 | 2.78 |
| 78376 | Ng23 | Ng23 protein | 77.8 | 28.0 | 2.78 |
| 231413 | Grsf1 | G-rich RNA sequence binding factor 1 | 12727.2 | 4597.7 | 2.77 |
| 55943 | Stx8 | syntaxin 8 | 62.7 | 22.6 | 2.77 |
| 16612 | Klk1 | kallikrein 1 | 41.5 | 15.0 | 2.76 |
| 18163 | Ctnnd2 | catenin (cadherin associated protein), delta 2 | 81.4 | 29.3 | 2.76 |
| 192287 | Slc25a36 | solute carrier family 25, member 36 | 654.7 | 237.1 | 2.75 |
| 69633 | 2310014D11Rik | RIKEN cDNA 2310014D11 gene | 2689.4 | 976.9 | 2.75 |
| 52829 | D4Bwg0951e | DNA segment, Chr 4, Brigham & Women's Genetics 0951 expressed | 309.6 | 112.6 | 2.75 |
| 246177 | Myo1g | myosin IG | 2237.2 | 815.5 | 2.74 |
| 14840 | Gsg1 | germ cell-specific gene 1 | 43.7 | 15.9 | 2.74 |
| 223864 | Rapgef3 | Rap guanine nucleotide exchange factor (GEF) 3 | 2787.2 | 1021.6 | 2.73 |
| 15200 | Hbegf | heparin-binding EGF-like growth factor | 2501.6 | 924.6 | 2.71 |
| 622434 | 4631416L12Rik | RIKEN cDNA 4631416L12 gene | 585.6 | 217.0 | 2.70 |
| 72433 | Rab38 | Rab38, member of RAS oncogene family | 117.1 | 43.4 | 2.70 |
| 212448 | 9330159F19Rik | RIKEN cDNA 9330159F19 gene | 70.1 | 26.0 | 2.69 |
| 83560 | Tex14 | testis expressed gene 14 | 607.8 | 225.9 | 2.69 |
| 76429 | 2310007H09Rik | RIKEN cDNA 2310007H09 gene | 7213.0 | 2686.2 | 2.68 |
| 233887 | Zfp553 | zinc finger protein 553 | 8834.2 | 3297.8 | 2.68 |
| 231821 | Centa1 | centaurin, alpha 1 | 1055.0 | 394.2 | 2.68 |
| 24113 | Vax2 | ventral anterior homeobox containing gene 2 | 88.5 | 33.0 | 2.68 |
| 212892 | Rshl3 | radial spokehead-like 3 | 39.1 | 14.4 | 2.67 |
| 59290 | Gpa33 | glycoprotein A33 (transmembrane) | 891.5 | 335.1 | 2.66 |
| 70906 | 4921522E08Rik | RIKEN cDNA 4921522E08 gene | 168.2 | 62.8 | 2.65 |
| 74144 | Robo4 | roundabout homolog 4 (Drosophila) | 3179.5 | 1201.2 | 2.65 |
| 14633 | Gli2 | GLI-Kruppel family member GLI2 | 1423.6 | 541.0 | 2.63 |
| 66435 | Ugcgl2 | UDP-glucose ceramide glucosyltransferase-like 2 | 166.0 | 63.2 | 2.63 |
| 232431 | Gprc5a | G protein-coupled receptor, family C, group 5, member A | 2901.4 | 1106.0 | 2.62 |
| 432516 | Myo1a | myosin IA | 429.5 | 164.4 | 2.61 |
| 218820 | Zfp503 | zinc finger protein 503 | 114.3 | 43.7 | 2.61 |
| 100213 | Rusc2 | RUN and SH3 domain containing 2 | 1827.7 | 700.4 | 2.61 |
| 22441 | Xlr | X-linked lymphocyte-regulated complex | 152.9 | 58.5 | 2.60 |
| 109791 | Clps | colipase, pancreatic | 87.1 | 33.8 | 2.58 |
| 106557 | Ldhal6b | lactate dehydrogenase A-like 6B | 157.6 | 61.2 | 2.58 |
| 14130 | Fcgr2b | Fc receptor, IgG, low affinity IIb | 74.8 | 29.0 | 2.58 |
| 12825 | Col3a1 | procollagen, type III, alpha 1 | 130.9 | 50.9 | 2.57 |
| 16336 | Insl3 | insulin-like 3 | 426.6 | 165.7 | 2.57 |
| 227696 | Phyhd1 | phytanoyl-CoA dioxygenase domain containing 1 | 1001.5 | 389.8 | 2.57 |
| 73053 | 2900078C09Rik | RIKEN cDNA 2900078C09 gene | 71.1 | 27.6 | 2.56 |
| 16186 | Il2rg | interleukin 2 receptor, gamma chain | 42.5 | 16.6 | 2.56 |
| 68527 | 1110017I16Rik | RIKEN cDNA 1110017I16 gene | 166.5 | 65.1 | 2.56 |
| 22715 | Zfp57 | zinc finger protein 57 | 708.5 | 277.5 | 2.55 |
| 242864 | AB112350 | cDNA sequence AB112350 | 272.1 | 105.9 | 2.55 |
| 20401 | Sh3bp1 | SH3-domain binding protein 1 | 1231.4 | 481.8 | 2.55 |
| 70296 | Tbc1d13 | TBC1 domain family, member 13 | 38161.6 | 14994.2 | 2.54 |
| 211945 | Plekhh1 | pleckstrin homology domain containing, family H (with MyTH4 domain) member 1 | 1317.1 | 517.7 | 2.53 |
| 20563 | Slit2 | slit homolog 2 (Drosophila) | 215.1 | 85.2 | 2.52 |
| 70450 | Unc13d | unc-13 homolog D (C. elegans) | 346.0 | 137.2 | 2.52 |
| 17884 | Myh4 | myosin, heavy polypeptide 4, skeletal muscle | 43.5 | 17.3 | 2.51 |
| 66277 | Klf15 | Kruppel-like factor 15 | 203.3 | 80.7 | 2.51 |
| 21767 | Tex264 | testis expressed gene 264 | 7700.0 | 3066.6 | 2.51 |
| 22612 | Yes1 | Yamaguchi sarcoma viral (v-yes) oncogene homolog 1 | 801.9 | 318.1 | 2.51 |
| 98952 | C230093N12Rik | RIKEN cDNA C230093N12 gene | 1141.2 | 460.6 | 2.47 |
| 77938 | A930008G19Rik | RIKEN cDNA A930008G19 gene | 105.5 | 42.6 | 2.47 |
| 70174 | 2210409B22Rik | RIKEN cDNA 2210409B22 gene | 1584.5 | 643.3 | 2.45 |
| 14912 | Nkx6-2 | NK6 transcription factor related, locus 2 (Drosophila) | 4356.0 | 1776.9 | 2.45 |
| 68750 | Rreb1 | ras responsive element binding protein 1 | 380.0 | 154.9 | 2.45 |
| 56748 | Hirip5 | histone cell cycle regulation defective interacting protein 5 | 127.5 | 52.3 | 2.44 |
| 75600 | Calml4 | calmodulin-like 4 | 162.9 | 66.9 | 2.43 |
| 16453 | Jak3 | Janus kinase 3 | 302.0 | 124.3 | 2.43 |
| 72747 | 2810439F02Rik | RIKEN cDNA 2810439F02 gene | 2647.9 | 1089.9 | 2.43 |
| 20404 | Sh3gl2 | SH3-domain GRB2-like 2 | 910.6 | 375.9 | 2.42 |
| 72148 | 2610019F03Rik | RIKEN cDNA 2610019F03 gene | 181.8 | 74.9 | 2.42 |
| 20104 | Rps6 | ribosomal protein S6 | 44.7 | 18.5 | 2.42 |
| 16598 | Klf2 | Kruppel-like factor 2 (lung) | 38165.1 | 15932.3 | 2.39 |
| 13710 | Elf3 | E74-like factor 3 | 1053.9 | 439.1 | 2.39 |
| 277978 | C730015A04Rik | RIKEN cDNA C730015A04 gene | 467.2 | 195.6 | 2.39 |
| 11931 | Atp1b1 | ATPase, Na+/K+ transporting, beta 1 polypeptide | 2411.4 | 1010.8 | 2.39 |
| 13511 | Dsg2 | desmoglein 2 | 577.6 | 241.7 | 2.38 |
| 13595 | Ebp | phenylalkylamine Ca2+ antagonist (emopamil) binding protein | 21311.8 | 8943.3 | 2.38 |
| 217379 | Ubxd4 | UBX domain containing 4 | 1795.5 | 754.0 | 2.38 |
| 68678 | Smtnl1 | smoothelin-like 1 | 2133.6 | 897.9 | 2.38 |
| 11828 | Aqp3 | aquaporin 3 | 7405.2 | 3118.6 | 2.38 |
| 17524 | Mpp1 | membrane protein, palmitoylated | 4721.7 | 1988.5 | 2.38 |
| 68947 | Chst8 | carbohydrate (N-acetylgalactosamine 4-0) sulfotransferase 8 | 1327.9 | 561.4 | 2.36 |
| 12955 | Cryab | crystallin, alpha B | 585.2 | 248.4 | 2.36 |
| 13838 | Epha4 | Eph receptor A4 | 642.7 | 273.7 | 2.35 |
| 13482 | Dpp4 | dipeptidylpeptidase 4 | 1657.6 | 705.7 | 2.34 |
| 70640 | Dcp2 | DCP2 decapping enzyme homolog (S. cerevisiae) | 346.7 | 148.0 | 2.34 |
| 19419 | Rasgrp1 | RAS guanyl releasing protein 1 | 169.8 | 72.6 | 2.34 |
| 66371 | 2310010I16Rik | RIKEN cDNA 2310010I16 gene | 722.6 | 309.0 | 2.34 |
| 14314 | Fstl1 | follistatin-like 1 | 7102.5 | 3038.6 | 2.34 |
| 19698 | Relb | avian reticuloendotheliosis viral (v-rel) oncogene related B | 3248.1 | 1391.2 | 2.34 |
| 18035 | Nfkbia | nuclear factor of kappa light chain gene enhancer in B-cells inhibitor, alpha | 11146.6 | 4777.3 | 2.33 |
| 93739 | Gabarapl2 | gamma-aminobutyric acid (GABA-A) receptor-associated protein-like 2 | 12318.6 | 5272.3 | 2.33 |
| 21425 | Tcfeb | transcription factor EB | 168.0 | 72.1 | 2.33 |
| 66214 | 1190002H23Rik | RIKEN cDNA 1190002H23 gene | 2203.4 | 945.3 | 2.33 |
| 105785 | Kdelr3 | KDEL (Lys-Asp-Glu-Leu) endoplasmic reticulum protein retention receptor 3 | 2535.7 | 1089.3 | 2.33 |
| 225884 | BC021614 | cDNA sequence BC021614 | 32.9 | 14.1 | 2.33 |
| 73690 | Glipr1 | GLI pathogenesis-related 1 (glioma) | 63.5 | 27.3 | 2.32 |
| 14313 | Fst | follistatin | 217.9 | 94.0 | 2.32 |
| 20443 | St3gal4 | ST3 beta-galactoside alpha-2,3-sialyltransferase 4 | 185.2 | 79.9 | 2.31 |
| 231252 | Chrna9 | cholinergic receptor, nicotinic, alpha polypeptide 9 | 725.6 | 313.8 | 2.31 |
| 12894 | Cpt1a | carnitine palmitoyltransferase 1a, liver | 511.6 | 221.7 | 2.31 |
| 17772 | Mtm1 | X-linked myotubular myopathy gene 1 | 57.0 | 24.4 | 2.31 |
| 67896 | Ccdc80 | coiled-coil domain containing 80 | 108.2 | 46.9 | 2.31 |
| 73137 | 1190002C06Rik | RIKEN cDNA 1190002C06 gene | 11267.0 | 4888.5 | 2.31 |
| 54169 | Myst4 | MYST histone acetyltransferase monocytic leukemia 4 | 2521.0 | 1093.5 | 2.30 |
| 22202 | Ube1y1 | ubiquitin-activating enzyme E1, Chr Y 1 | 457.2 | 198.5 | 2.30 |
| 101602 | AI467606 | expressed sequence AI467606 | 699.2 | 303.9 | 2.30 |
| 18187 | Nrp2 | neuropilin 2 | 2916.1 | 1269.6 | 2.30 |
| 217837 | Itpk1 | inositol 1,3,4-triphosphate 5/6 kinase | 10166.2 | 4428.0 | 2.30 |
| 12035 | Bcat1 | branched chain aminotransferase 1, cytosolic | 16259.2 | 7058.3 | 2.30 |
| 216851 | Dnahc2 | dynein, axonemal, heavy chain 2 | 124.6 | 54.1 | 2.30 |
| 70122 | Mllt3 | myeloid/lymphoid or mixed lineage-leukemia translocation to 3 homolog (Drosophila) | 255.6 | 111.2 | 2.29 |
| 330938 | Dixdc1 | DIX domain containing 1 | 161.4 | 70.3 | 2.29 |
| 18596 | Pdgfrb | platelet derived growth factor receptor, beta polypeptide | 285.6 | 124.5 | 2.29 |
| 16331 | Inpp5d | inositol polyphosphate-5-phosphatase D | 1550.8 | 673.6 | 2.29 |
| 20262 | Stmn3 | stathmin-like 3 | 786.4 | 343.3 | 2.29 |
| 17896 | Myl4 | myosin, light polypeptide 4 | 248.7 | 109.1 | 2.28 |
| 77771 | A330102K23Rik | RIKEN cDNA A330102K23 gene | 100.4 | 44.0 | 2.28 |
| 56315 | Rhcg | Rhesus blood group-associated C glycoprotein | 116.2 | 51.2 | 2.27 |
| 74134 | Cyp2s1 | cytochrome P450, family 2, subfamily s, polypeptide 1 | 3667.0 | 1615.4 | 2.27 |
| 66346 | 1700029P11Rik | RIKEN cDNA 1700029P11 gene | 5496.2 | 2429.7 | 2.26 |
| 17076 | Ly75 | lymphocyte antigen 75 | 553.9 | 244.5 | 2.26 |
| 19725 | Rfx2 | regulatory factor X, 2 (influences HLA class II expression) | 614.4 | 272.1 | 2.26 |
| 74400 | 4933405K07Rik | RIKEN cDNA 4933405K07 gene | 5148.8 | 2278.4 | 2.26 |
| 20737 | Spn | sialophorin | 812.9 | 359.4 | 2.26 |
| 63872 | Zfp296 | zinc finger protein 296 | 14255.0 | 6313.0 | 2.26 |
| 56791 | Ube2l6 | ubiquitin-conjugating enzyme E2L 6 | 138.1 | 60.9 | 2.26 |
| 74559 | Elovl7 | ELOVL family member 7, elongation of long chain fatty acids (yeast) | 835.9 | 371.2 | 2.25 |
| 74365 | Lonrf3 | LON peptidase N-terminal domain and ring finger 3 | 389.2 | 172.5 | 2.25 |
| 72027 | Slc39a4 | solute carrier family 39 (zinc transporter), member 4 | 15514.2 | 6897.3 | 2.25 |
| 71911 | Bdh1 | 3-hydroxybutyrate dehydrogenase, type 1 | 7696.5 | 3422.8 | 2.25 |
| 79459 | Aldoa-ps1 | aldolase 1, A isoform, pseudogene 1 | 35.3 | 15.6 | 2.25 |
| 70536 | Qpct | glutaminyl-peptide cyclotransferase (glutaminyl cyclase) | 113.9 | 50.5 | 2.25 |
| 20431 | Si | silver | 688.9 | 307.1 | 2.24 |
| 231861 | Zfp469 | zinc finger protein 469 | 559.8 | 247.9 | 2.24 |
| 77106 | Gpr178 | G protein-coupled receptor 178 | 1412.9 | 629.6 | 2.24 |
| 72685 | Dnajc6 | DnaJ (Hsp40) homolog, subfamily C, member 6 | 839.4 | 374.9 | 2.24 |
| 74561 | Nkx6-3 | NK6 transcription factor related, locus 3 (Drosophila) | 268.4 | 120.2 | 2.23 |
| 94092 | Trim16 | tripartite motif protein 16 | 338.7 | 152.1 | 2.23 |
| 106347 | Ildr1 | immunoglobulin-like domain containing receptor 1 | 274.9 | 123.5 | 2.22 |
| 70274 | Ly6g6e | lymphocyte antigen 6 complex, locus G6E | 1188.1 | 535.2 | 2.22 |
| 16177 | Il1r1 | interleukin 1 receptor, type I | 33.1 | 14.9 | 2.21 |
| 22722 | Zfp64 | zinc finger protein 64 | 340.6 | 154.0 | 2.21 |
| 243220 | 4933413A10Rik | RIKEN cDNA 4933413A10 gene | 1357.7 | 615.0 | 2.21 |
| 14797 | Aes | amino-terminal enhancer of split | 2279.8 | 1035.2 | 2.20 |
| 214133 | E130014J05Rik | RIKEN cDNA E130014J05 gene | 705.9 | 318.9 | 2.20 |
| 666415 | LOC666415 | hypothetical protein LOC666415 | 61.3 | 27.8 | 2.20 |
| 319504 | C130076O07Rik | RIKEN cDNA C130076O07 gene | 459.7 | 209.1 | 2.20 |
| 12716 | Ckmt1 | creatine kinase, mitochondrial 1, ubiquitous | 1008.0 | 462.1 | 2.18 |
| 16969 | Zbtb7a | zinc finger and BTB domain containing 7a | 514.3 | 235.3 | 2.18 |
| 76071 | Jakmip1 | janus kinase and microtubule interacting protein 1 | 282.2 | 129.4 | 2.18 |
| 11813 | Apoc2 | apolipoprotein C-II | 153.9 | 70.7 | 2.17 |
| 16149 | Cd74 | CD74 antigen (invariant polypeptide of major histocompatibility complex, class II antigen-associated) | 224.8 | 103.5 | 2.17 |
| 16774 | Lama3 | laminin, alpha 3 | 404.3 | 186.4 | 2.17 |
| 70948 | Wdr20 | WD repeat domain 20 | 89.6 | 41.4 | 2.16 |
| 238076 | Kcns3 | potassium voltage-gated channel, delayed-rectifier, subfamily S, member 3 | 155.3 | 71.7 | 2.16 |
| 12705 | Cited1 | Cbp/p300-interacting transactivator with Glu/Asp-rich carboxy-terminal domain 1 | 570.5 | 264.1 | 2.16 |
| 13730 | Emp1 | epithelial membrane protein 1 | 258.6 | 119.2 | 2.16 |
| 665756 | LOC665756 | hypothetical protein LOC665756 | 186.0 | 86.1 | 2.16 |
| 14969 | H2-Eb1 | histocompatibility 2, class II antigen E beta | 150.7 | 70.1 | 2.15 |
| 12709 | Ckb | creatine kinase, brain | 25831.0 | 11915.0 | 2.15 |
| 232146 | BC014699 | cDNA sequence BC014699 | 331.3 | 154.1 | 2.15 |
| 71020 | Spats1 | spermatogenesis associated, serine-rich 1 | 71.4 | 33.1 | 2.15 |
| 677447 | LOC677447 | similar to RIKEN cDNA 5730590G19-like | 583.2 | 271.6 | 2.15 |
| 333669 | LOC333669 | hypothetical gene supported by AK041995 | 93.9 | 43.7 | 2.14 |
| 19012 | Ppap2a | phosphatidic acid phosphatase 2a | 2990.3 | 1394.6 | 2.14 |
| 14268 | Fn1 | fibronectin 1 | 11738.5 | 5483.7 | 2.14 |
| 80888 | Hspb8 | heat shock protein 8 | 1838.3 | 860.4 | 2.13 |
| 333182 | Cox6b2 | cytochrome c oxidase subunit VIb polypeptide 2 | 2464.2 | 1153.6 | 2.13 |
| 209630 | Frmd4a | FERM domain containing 4A | 533.7 | 248.3 | 2.13 |
| 97895 | Nalp4f | NACHT, leucine rich repeat and PYD containing 4F | 1063.0 | 497.5 | 2.13 |
| 11636 | Ak1 | adenylate kinase 1 | 3959.1 | 1863.5 | 2.12 |
| 19038 | Ppic | peptidylprolyl isomerase C | 9538.0 | 4486.7 | 2.12 |
| 14615 | Gja7 | gap junction membrane channel protein alpha 7 | 8441.8 | 3968.3 | 2.12 |
| 20257 | Stmn2 | stathmin-like 2 | 3053.8 | 1436.6 | 2.12 |
| 66686 | Dcbld1 | discoidin, CUB and LCCL domain containing 1 | 1564.9 | 739.8 | 2.12 |
| 71887 | Ppm1j | protein phosphatase 1J | 2313.0 | 1094.6 | 2.11 |
| 17532 | Mras | muscle and microspikes RAS | 1212.8 | 572.2 | 2.11 |
| 17762 | Mapt | microtubule-associated protein tau | 763.9 | 361.6 | 2.11 |
| 102857 | Slc6a8 | solute carrier family 6 (neurotransmitter transporter, creatine), member 8 | 4324.7 | 2048.9 | 2.11 |
| 57279 | Slc25a20 | solute carrier family 25 (mitochondrial carnitine/acylcarnitine translocase), member 20 | 2136.7 | 1011.1 | 2.11 |
| 381067 | BC043476 | cDNA sequence BC043476 | 154.7 | 72.8 | 2.10 |
| 65970 | Lima1 | LIM domain and actin binding 1 | 15101.8 | 7171.1 | 2.10 |
| 18787 | Serpine1 | serine (or cysteine) peptidase inhibitor, clade E, member 1 | 470.7 | 223.0 | 2.10 |
| 242584 | Wdr78 | WD repeat domain 78 | 302.2 | 144.2 | 2.09 |
| 242484 | D630039A03Rik | RIKEN cDNA D630039A03 gene | 294.9 | 140.9 | 2.09 |
| 382161 | LOC382161 | similar to zinc finger protein 124 | 556.5 | 265.9 | 2.09 |
| 226422 | Rab7l1 | RAB7, member RAS oncogene family-like 1 | 501.2 | 239.0 | 2.09 |
| 78781 | Zc3hav1 | zinc finger CCCH type, antiviral 1 | 1358.4 | 648.7 | 2.09 |
| 435271 | LOC435271 | similar to schlafen 3 | 369.7 | 175.9 | 2.09 |
| 243937 | Zfp536 | zinc finger protein 536 | 355.7 | 170.4 | 2.09 |
| 263406 | Plekhg3 | pleckstrin homology domain containing, family G (with RhoGef domain) member 3 | 1978.7 | 946.9 | 2.09 |
| 66819 | 9130422G05Rik | RIKEN cDNA 9130422G05 gene | 592.1 | 283.8 | 2.08 |
| 665798 | LOC665798 | similar to aurora kinase C | 480.4 | 230.4 | 2.08 |
| 74959 | 4930500J02Rik | RIKEN cDNA 4930500J02 gene | 1549.9 | 744.4 | 2.08 |
| 66395 | Ahnak | AHNAK nucleoprotein (desmoyokin) | 1855.8 | 887.2 | 2.08 |
| 69863 | 1810054D07Rik | RIKEN cDNA 1810054D07 gene | 1026.9 | 494.0 | 2.08 |
| 12224 | Klf5 | Kruppel-like factor 5 | 9804.3 | 4719.6 | 2.08 |
| 11992 | Auh | AU RNA binding protein/enoyl-coenzyme A hydratase | 864.9 | 415.4 | 2.08 |
| 56386 | B4galt6 | UDP-Gal:betaGlcNAc beta 1,4-galactosyltransferase, polypeptide 6 | 1247.8 | 603.9 | 2.07 |
| 21808 | Tgfb2 | transforming growth factor, beta 2 | 103.1 | 50.2 | 2.06 |
| 434459 | LOC434459 | similar to RIKEN cDNA 4930503E14 | 63.5 | 31.0 | 2.05 |
| 74136 | Sec14l1 | SEC14-like 1 (S. cerevisiae) | 610.0 | 297.7 | 2.05 |
| 74107 | Cep55 | centrosomal protein 55 | 2946.8 | 1436.6 | 2.05 |
| 68775 | Atp6v1c2 | ATPase, H+ transporting, lysosomal V1 subunit C2 | 72.1 | 35.2 | 2.04 |
| 244551 | Nanos3 | nanos homolog 3 (Drosophila) | 84.9 | 41.5 | 2.04 |
| 207565 | Camkk2 | calcium/calmodulin-dependent protein kinase kinase 2, beta | 955.8 | 468.0 | 2.04 |
| 108012 | Ap1s2 | adaptor-related protein complex 1, sigma 2 subunit | 812.7 | 397.1 | 2.04 |
| 20871 | Aurkc | aurora kinase C | 473.7 | 232.1 | 2.04 |
| 15985 | Cd79b | CD79B antigen | 416.5 | 204.9 | 2.03 |
| 12571 | Cdk6 | cyclin-dependent kinase 6 | 2212.7 | 1085.0 | 2.03 |
| 11647 | Akp2 | alkaline phosphatase 2, liver | 12125.4 | 5922.9 | 2.03 |
| 16407 | Itgae | integrin, alpha E, epithelial-associated | 234.8 | 115.7 | 2.03 |
| 231287 | Atp10d | ATPase, Class V, type 10D | 111.2 | 54.6 | 2.03 |
| 105855 | Nckap1l | NCK associated protein 1 like | 435.0 | 214.5 | 2.03 |
| 12308 | Calb2 | calbindin 2 | 35.7 | 17.6 | 2.03 |
| 22702 | Zfp42 | zinc finger protein 42 | 7328.2 | 3615.5 | 2.02 |
| 654795 | 2310014G06Rik | RIKEN cDNA 2310014G06 gene | 2480.0 | 1225.6 | 2.02 |
| 20908 | Stx3 | syntaxin 3 | 71.9 | 35.5 | 2.02 |
| 234678 | D230025D16Rik | RIKEN cDNA D230025D16 gene | 1686.0 | 832.2 | 2.02 |
| 12587 | Mia1 | melanoma inhibitory activity 1 | 476.2 | 235.8 | 2.02 |
| 16782 | Lamc2 | laminin, gamma 2 | 1104.3 | 547.4 | 2.02 |
| 22788 | Zp3 | zona pellucida glycoprotein 3 | 2051.3 | 1019.6 | 2.01 |
| 114654 | Ly6g6d | lymphocyte antigen 6 complex, locus G6D | 201.9 | 100.9 | 2.00 |
