# Supplementary material for: Stable generation of serum- and feeder-free embryonic stem cell-derived mice with full germline-competency by using a GSK3 specific inhibitor
Source: Genesis. 2009 Apr 23;47(6):414–22. doi: 10.1002/dvg.20514 (PMC2726955; doi:10.1002/dvg.20514)
Supplement: Supplementary file 8 [file dvg0047-0414-SD8.doc]

Supplementary Table 2

| **Entrez Gene ID** | **Gene Symbol** | **Gene Name** | **1000U/ml LIF + 2 μM BIO** | **1000 U/ml LIF** | **Ratio(1000 U/ml LIF + 2 μM BIO / 1000 U/ml LIF)** |
| --- | --- | --- | --- | --- | --- |
| 12351 | Car4 | carbonic anhydrase 4 | 48.1 | 915.8 | 0.05 |
| 22431 | Wt1 | Wilms tumor homolog | 34.7 | 647.1 | 0.05 |
| 18991 | Pou3f1 | POU domain, class 3, transcription factor 1 | 3152.6 | 57382.0 | 0.05 |
| 76217 | Jakmip2 | janus kinase and microtubule interacting protein 2 | 18.9 | 337.1 | 0.06 |
| 252837 | Ccrl1 | chemokine (C-C motif) receptor-like 1 | 16.1 | 285.8 | 0.06 |
| 18993 | Pou3f3 | POU domain, class 3, transcription factor 3 | 69.7 | 1233.8 | 0.06 |
| 69299 | Asb9 | ankyrin repeat and SOCS box-containing protein 9 | 26.4 | 457.8 | 0.06 |
| 80976 | Syt13 | synaptotagmin XIII | 93.6 | 1244.1 | 0.08 |
| 72014 | 1500005I02Rik | RIKEN cDNA 1500005I02 gene | 34.3 | 447.9 | 0.08 |
| 18424 | Otx2 | orthodenticle homolog 2 (Drosophila) | 46.4 | 580.7 | 0.08 |
| 14725 | Lrp2 | low density lipoprotein receptor-related protein 2 | 319.8 | 3841.1 | 0.08 |
| 14176 | Fgf5 | fibroblast growth factor 5 | 30.4 | 359.4 | 0.08 |
| 71932 | Abhd9 | abhydrolase domain containing 9 | 23.7 | 277.7 | 0.09 |
| 106522 | AW548124 | expressed sequence AW548124 | 143.4 | 1673.1 | 0.09 |
| 18212 | Ntrk2 | neurotrophic tyrosine kinase, receptor, type 2 | 29.5 | 332.1 | 0.09 |
| 66959 | Dusp26 | dual specificity phosphatase 26 (putative) | 28.7 | 305.3 | 0.09 |
| 23962 | Oasl2 | 2'-5' oligoadenylate synthetase-like 2 | 53.3 | 526.1 | 0.10 |
| 269023 | Zfp608 | zinc finger protein 608 | 26.4 | 260.3 | 0.10 |
| 23831 | Car14 | carbonic anhydrase 14 | 106.8 | 1028.5 | 0.10 |
| 242506 | Frmd3 | FERM domain containing 3 | 18.2 | 168.0 | 0.11 |
| 20317 | Serpinf1 | serine (or cysteine) peptidase inhibitor, clade F, member 1 | 157.2 | 1494.0 | 0.11 |
| 21828 | Thbs4 | thrombospondin 4 | 17.4 | 156.9 | 0.11 |
| 14183 | Fgfr2 | fibroblast growth factor receptor 2 | 45.9 | 413.1 | 0.11 |
| 76969 | Chst1 | carbohydrate (keratan sulfate Gal-6) sulfotransferase 1 | 33.8 | 305.1 | 0.11 |
| 20522 | Slc23a1 | solute carrier family 23 (nucleobase transporters), member 1 | 21.4 | 186.7 | 0.11 |
| 11535 | Adm | adrenomedullin | 239.7 | 2091.1 | 0.11 |
| 18606 | Enpp2 | ectonucleotide pyrophosphatase/phosphodiesterase 2 | 25.2 | 213.7 | 0.12 |
| 100102 | Pcsk9 | proprotein convertase subtilisin/kexin type 9 | 195.5 | 1680.2 | 0.12 |
| 20672 | Sox18 | SRY-box containing gene 18 | 30.7 | 256.9 | 0.12 |
| 75770 | Brsk2 | BR serine/threonine kinase 2 | 40.2 | 333.2 | 0.12 |
| 240047 | Mmp25 | matrix metallopeptidase 25 | 30.6 | 247.7 | 0.12 |
| 676870 | LOC676870 | region containing RIKEN cDNA 2310056B04 gene; pre B-cell leukemia transcription factor 1 | 51.7 | 419.8 | 0.12 |
| 140904 | Caln1 | calneuron 1 | 19.6 | 156.9 | 0.13 |
| 20502 | Slc16a2 | solute carrier family 16 (monocarboxylic acid transporters), member 2 | 95.0 | 757.1 | 0.13 |
| 66260 | Tmem54 | transmembrane protein 54 | 409.6 | 3239.9 | 0.13 |
| 12349 | Car2 | carbonic anhydrase 2 | 338.0 | 2666.1 | 0.13 |
| 19242 | Ptn | pleiotrophin | 21.8 | 166.5 | 0.13 |
| 53626 | Insm1 | insulinoma-associated 1 | 17.1 | 132.2 | 0.13 |
| 68588 | Cthrc1 | collagen triple helix repeat containing 1 | 84.5 | 646.3 | 0.13 |
| 19228 | Pthr1 | parathyroid hormone receptor 1 | 135.9 | 1001.4 | 0.14 |
| 65969 | Cubn | cubilin (intrinsic factor-cobalamin receptor) | 15.3 | 110.8 | 0.14 |
| 14170 | Fgf15 | fibroblast growth factor 15 | 54.5 | 351.6 | 0.16 |
| 270097 | AI427515 | expressed sequence AI427515 | 31.6 | 200.2 | 0.16 |
| 14025 | Bcl11a | B-cell CLL/lymphoma 11A (zinc finger protein) | 33.6 | 212.2 | 0.16 |
| 18715 | Pim2 | proviral integration site 2 | 1161.8 | 7292.6 | 0.16 |
| 16913 | Psmb8 | proteosome (prosome, macropain) subunit, beta type 8 (large multifunctional peptidase 7) | 92.2 | 569.2 | 0.16 |
| 11553 | Adra2c | adrenergic receptor, alpha 2c | 14.8 | 91.1 | 0.16 |
| 19132 | Prph1 | peripherin 1 | 234.0 | 1417.3 | 0.16 |
| 333564 | Gm784 | gene model 784, (NCBI) | 169.8 | 1015.5 | 0.17 |
| 12287 | Cacna1b | calcium channel, voltage-dependent, N type, alpha 1B subunit | 17.8 | 103.7 | 0.17 |
| 108116 | Slco3a1 | solute carrier organic anion transporter family, member 3a1 | 90.4 | 516.5 | 0.17 |
| 14842 | Gsh1 | genomic screened homeo box 1 | 66.2 | 369.1 | 0.17 |
| 20451 | St8sia3 | ST8 alpha-N-acetyl-neuraminide alpha-2,8-sialyltransferase 3 | 41.2 | 231.5 | 0.18 |
| 207667 | Lbxcor1 | ladybird homeobox 1 homolog (Drosophila) corepressor 1 | 61.6 | 344.2 | 0.18 |
| 381157 | AK220484 | cDNA sequence AK220484 | 227.0 | 1268.4 | 0.18 |
| 22360 | Nrsn1 | neurensin 1 | 174.2 | 971.4 | 0.18 |
| 110805 | Foxe1 | forkhead box E1 (thyroid transcription factor 2) | 21.6 | 118.7 | 0.18 |
| 403178 | Plcxd1 | phosphatidylinositol-specific phospholipase C, X domain containing 1 | 91.2 | 503.3 | 0.18 |
| 15117 | Has2 | hyaluronan synthase 2 | 51.3 | 280.1 | 0.18 |
| 68952 | 1500016O10Rik | RIKEN cDNA 1500016O10 gene | 36.3 | 195.6 | 0.19 |
| 102871 | D330045A20Rik | RIKEN cDNA D330045A20 gene | 26.6 | 138.2 | 0.19 |
| 66892 | Eif4e3 | eukaryotic translation initiation factor 4E member 3 | 42.4 | 222.4 | 0.19 |
| 16439 | Itpr2 | inositol 1,4,5-triphosphate receptor 2 | 51.1 | 268.5 | 0.19 |
| 29857 | Mapk12 | mitogen-activated protein kinase 12 | 528.2 | 2724.7 | 0.19 |
| 20230 | Satb1 | special AT-rich sequence binding protein 1 | 123.9 | 634.3 | 0.20 |
| 19331 | Rab19 | RAB19, member RAS oncogene family | 78.5 | 400.6 | 0.20 |
| 14758 | Gpm6b | glycoprotein m6b | 35.8 | 178.4 | 0.20 |
| 257635 | Sdsl | serine dehydratase-like | 14.2 | 71.3 | 0.20 |
| 74568 | Mlkl | mixed lineage kinase domain-like | 40.6 | 201.4 | 0.20 |
| 13642 | Efnb2 | ephrin B2 | 237.7 | 1194.7 | 0.20 |
| 14121 | Fbp1 | fructose bisphosphatase 1 | 29.1 | 138.7 | 0.20 |
| 12064 | Bdnf | brain derived neurotrophic factor | 59.3 | 294.2 | 0.20 |
| 57754 | Cend1 | cell cycle exit and neuronal differentiation 1 | 52.4 | 258.6 | 0.20 |
| 14428 | Galr2 | galanin receptor 2 | 69.6 | 342.5 | 0.20 |
| 26458 | Slc27a2 | solute carrier family 27 (fatty acid transporter), member 2 | 515.6 | 2519.6 | 0.20 |
| 103889 | Hoxb2 | homeo box B2 | 118.1 | 572.9 | 0.21 |
| 12292 | Cacna1s | calcium channel, voltage-dependent, L type, alpha 1S subunit | 51.4 | 247.5 | 0.21 |
| 217012 | Unc45b | unc-45 homolog B (C. elegans) | 66.7 | 322.4 | 0.21 |
| 53416 | Stk39 | serine/threonine kinase 39, STE20/SPS1 homolog (yeast) | 124.4 | 601.0 | 0.21 |
| 16560 | Kif1a | kinesin family member 1A | 513.8 | 2481.1 | 0.21 |
| 78784 | Tnrc4 | trinucleotide repeat containing 4 | 20.3 | 94.7 | 0.21 |
| 16467 | Atcay | ataxia, cerebellar, Cayman type homolog (human) | 18.3 | 86.1 | 0.21 |
| 17534 | Mrc2 | mannose receptor, C type 2 | 85.7 | 412.0 | 0.21 |
| 13132 | Dab2 | disabled homolog 2 (Drosophila) | 46.7 | 224.3 | 0.21 |
| 384061 | Fndc5 | fibronectin type III domain containing 5 | 124.9 | 596.8 | 0.21 |
| 319984 | Jph4 | junctophilin 4 | 96.8 | 460.9 | 0.21 |
| 76293 | Mfap4 | microfibrillar-associated protein 4 | 78.6 | 369.1 | 0.21 |
| 381813 | Prmt8 | protein arginine N-methyltransferase 8 | 87.0 | 403.2 | 0.21 |
| 18514 | Pbx1 | pre B-cell leukemia transcription factor 1 | 15.9 | 73.8 | 0.21 |
| 67564 | Tmem35 | transmembrane protein 35 | 26.0 | 121.1 | 0.21 |
| 74189 | Phactr3 | phosphatase and actin regulator 3 | 110.1 | 510.0 | 0.22 |
| 14469 | Gbp2 | guanylate nucleotide binding protein 2 | 135.0 | 620.5 | 0.22 |
| 242939 | Cpz | carboxypeptidase Z | 22.0 | 98.9 | 0.22 |
| 278672 | 1110051B16Rik | RIKEN cDNA 1110051B16 gene | 106.9 | 480.0 | 0.22 |
| 71691 | 0710005I19Rik | RIKEN cDNA 0710005I19 gene | 20.4 | 90.5 | 0.22 |
| 106014 | AW049604 | expressed sequence AW049604 | 21.0 | 94.0 | 0.22 |
| 50524 | Sall2 | sal-like 2 (Drosophila) | 278.4 | 1241.6 | 0.22 |
| 207521 | Dtx4 | deltex 4 homolog (Drosophila) | 257.0 | 1145.4 | 0.22 |
| 13491 | Drd4 | dopamine receptor 4 | 36.3 | 161.5 | 0.22 |
| 21355 | Tap2 | transporter 2, ATP-binding cassette, sub-family B (MDR/TAP) | 28.4 | 126.2 | 0.22 |
| 211468 | Kcnh8 | potassium voltage-gated channel, subfamily H (eag-related), member 8 | 17.5 | 76.7 | 0.23 |
| 18751 | Prkcb1 | protein kinase C, beta 1 | 32.7 | 144.4 | 0.23 |
| 73340 | Nptxr | neuronal pentraxin receptor | 80.5 | 354.5 | 0.23 |
| 207182 | Ggtl3 | gamma-glutamyltransferase-like 3 | 39.3 | 173.0 | 0.23 |
| 15019 | H2-Q8 | histocompatibility 2, Q region locus 8 | 194.2 | 854.6 | 0.23 |
| 209558 | Enpp3 | ectonucleotide pyrophosphatase/phosphodiesterase 3 | 752.0 | 3292.3 | 0.23 |
| 70696 | 3830417A13Rik | RIKEN cDNA 3830417A13 gene | 24.0 | 104.2 | 0.23 |
| 20315 | Cxcl12 | chemokine (C-X-C motif) ligand 12 | 298.6 | 1274.5 | 0.23 |
| 434784 | Ldoc1 | leucine zipper, down-regulated in cancer 1 | 15.8 | 67.2 | 0.23 |
| 207474 | Kctd12b | potassium channel tetramerisation domain containing 12b | 50.9 | 212.1 | 0.24 |
| 59012 | Moxd1 | monooxygenase, DBH-like 1 | 194.8 | 821.9 | 0.24 |
| 17150 | Mfap2 | microfibrillar-associated protein 2 | 149.8 | 628.4 | 0.24 |
| 64051 | Sv2a | synaptic vesicle glycoprotein 2 a | 22.4 | 92.1 | 0.24 |
| 78303 | Hist3h2ba | histone 3, H2ba | 465.6 | 1945.2 | 0.24 |
| 228858 | Gdap1l1 | ganglioside-induced differentiation-associated protein 1-like 1 | 153.3 | 638.5 | 0.24 |
| 241520 | D430039N05Rik | RIKEN cDNA D430039N05 gene | 433.4 | 1767.3 | 0.24 |
| 56876 | Nelf | nasal embryonic LHRH factor | 537.4 | 2200.0 | 0.24 |
| 21350 | Tal2 | T-cell acute lymphocytic leukemia 2 | 38.0 | 155.1 | 0.24 |
| 99296 | Hrh3 | histamine receptor H 3 | 73.1 | 298.5 | 0.24 |
| 240675 | Vwa2 | von Willebrand factor A domain containing 2 | 42.5 | 173.1 | 0.24 |
| 209195 | Clic6 | chloride intracellular channel 6 | 58.5 | 239.0 | 0.24 |
| 13082 | Cyp26a1 | cytochrome P450, family 26, subfamily a, polypeptide 1 | 57.6 | 234.7 | 0.25 |
| 107448 | Unc5a | unc-5 homolog A (C. elegans) | 65.7 | 265.4 | 0.25 |
| 76156 | 6330503C03Rik | RIKEN cDNA 6330503C03 gene | 30.7 | 123.9 | 0.25 |
| 66725 | Lrrk2 | leucine-rich repeat kinase 2 | 53.6 | 217.0 | 0.25 |
| 72514 | 2610306H15Rik | RIKEN cDNA 2610306H15 gene | 20.6 | 82.0 | 0.25 |
| 13389 | Dll3 | delta-like 3 (Drosophila) | 232.6 | 939.4 | 0.25 |
| 18508 | Pax6 | paired box gene 6 | 15.5 | 61.9 | 0.25 |
| 18389 | Oprl1 | opioid receptor-like 1 | 24.0 | 95.6 | 0.25 |
| 58203 | Zbp1 | Z-DNA binding protein 1 | 57.5 | 228.5 | 0.25 |
| 231238 | 2310045A20Rik | RIKEN cDNA 2310045A20 gene | 22.1 | 87.8 | 0.25 |
| 17122 | Mxd4 | Max dimerization protein 4 | 39.7 | 156.6 | 0.25 |
| 74016 | Phf19 | PHD finger protein 19 | 301.2 | 1192.3 | 0.25 |
| 71306 | Mfap3l | microfibrillar-associated protein 3-like | 23.6 | 92.0 | 0.25 |
| 63828 | Fn3k | fructosamine 3 kinase | 20.9 | 82.1 | 0.25 |
| 69847 | Wnk4 | WNK lysine deficient protein kinase 4 | 23.8 | 93.1 | 0.25 |
| 241324 | Crb2 | crumbs homolog 2 (Drosophila) | 37.9 | 147.2 | 0.26 |
| 77590 | 4631426J05Rik | RIKEN cDNA 4631426J05 gene | 48.7 | 187.8 | 0.26 |
| 320974 | B430119L13Rik | RIKEN cDNA B430119L13 gene | 111.1 | 430.4 | 0.26 |
| 19249 | Ptpn13 | protein tyrosine phosphatase, non-receptor type 13 | 582.2 | 2243.3 | 0.26 |
| 14559 | Gdf1 | growth differentiation factor 1 | 2527.5 | 9720.8 | 0.26 |
| 69538 | Antxr1 | anthrax toxin receptor 1 | 67.3 | 257.6 | 0.26 |
| 209378 | Itih5 | inter-alpha (globulin) inhibitor H5 | 30.9 | 116.6 | 0.26 |
| 554327 | LOC554327 | hypothetical protein LOC554327 | 18.7 | 71.4 | 0.26 |
| 20420 | Shd | src homology 2 domain-containing transforming protein D | 92.0 | 351.4 | 0.26 |
| 14675 | Gna14 | guanine nucleotide binding protein, alpha 14 | 71.8 | 272.5 | 0.26 |
| 214791 | Sertad4 | SERTA domain containing 4 | 104.8 | 395.5 | 0.26 |
| 104384 | Rhox9 | reproductive homeobox 9 | 1240.8 | 4713.1 | 0.26 |
| 218194 | Phactr1 | phosphatase and actin regulator 1 | 45.3 | 171.9 | 0.26 |
| 65255 | Asb4 | ankyrin repeat and SOCS box-containing protein 4 | 47.1 | 177.4 | 0.26 |
| 16909 | Lmo2 | LIM domain only 2 | 15.5 | 57.8 | 0.27 |
| 26382 | Fgd2 | FYVE, RhoGEF and PH domain containing 2 | 47.8 | 179.7 | 0.27 |
| 277432 | Gm691 | gene model 691, (NCBI) | 52.3 | 193.9 | 0.27 |
| 18810 | Plec1 | plectin 1 | 138.3 | 511.7 | 0.27 |
| 13388 | Dll1 | delta-like 1 (Drosophila) | 67.3 | 246.4 | 0.27 |
| 66104 | 1500026B10Rik | RIKEN cDNA 1500026B10 gene | 132.6 | 488.0 | 0.27 |
| 12609 | Cebpd | CCAAT/enhancer binding protein (C/EBP), delta | 832.0 | 3051.7 | 0.27 |
| 30956 | Aass | aminoadipate-semialdehyde synthase | 133.5 | 489.2 | 0.27 |
| 269642 | 1110038O08Rik | RIKEN cDNA 1110038O08 gene | 591.9 | 2152.9 | 0.27 |
| 11517 | Adcyap1r1 | adenylate cyclase activating polypeptide 1 receptor 1 | 17.3 | 62.4 | 0.28 |
| 229521 | Syt11 | synaptotagmin XI | 798.0 | 2880.5 | 0.28 |
| 210029 | Metrnl | meteorin, glial cell differentiation regulator-like | 138.7 | 500.0 | 0.28 |
| 74175 | 2300002G24Rik | RIKEN cDNA 2300002G24 gene | 117.9 | 421.9 | 0.28 |
| 23985 | Slc26a4 | solute carrier family 26, member 4 | 17.3 | 61.5 | 0.28 |
| 594844 | Tceal3 | transcription elongation factor A (SII)-like 3 | 71.7 | 255.0 | 0.28 |
| 53945 | Slc40a1 | solute carrier family 40 (iron-regulated transporter), member 1 | 182.2 | 643.6 | 0.28 |
| 67198 | 2810022L02Rik | RIKEN cDNA 2810022L02 gene | 232.7 | 824.4 | 0.28 |
| 57340 | Jph3 | junctophilin 3 | 88.2 | 311.6 | 0.28 |
| 270109 | E330039K12Rik | RIKEN cDNA E330039K12 gene | 102.6 | 361.4 | 0.28 |
| 229722 | 5330417C22Rik | RIKEN cDNA 5330417C22 gene | 34.3 | 120.7 | 0.28 |
| 13449 | Dok2 | docking protein 2 | 3805.8 | 13357.3 | 0.29 |
| 70031 | Cmtm8 | CKLF-like MARVEL transmembrane domain containing 8 | 666.5 | 2321.8 | 0.29 |
| 19202 | Rhox6 | reproductive homeobox 6 | 979.6 | 3414.5 | 0.29 |
| 192198 | Lrrc4 | leucine rich repeat containing 4 | 64.9 | 226.2 | 0.29 |
| 212733 | BC038613 | cDNA sequence BC038613 | 167.2 | 582.5 | 0.29 |
| 20776 | Tmie | transmembrane inner ear | 21.1 | 73.3 | 0.29 |
| 13590 | Lefty1 | left right determination factor 1 | 11293.4 | 39287.5 | 0.29 |
| 269788 | Lhfpl4 | lipoma HMGIC fusion partner-like protein 4 | 147.6 | 512.7 | 0.29 |
| 209268 | Igsf1 | immunoglobulin superfamily, member 1 | 32.5 | 112.0 | 0.29 |
| 16564 | Kif21a | kinesin family member 21A | 32.4 | 110.3 | 0.29 |
| 69665 | 2310043J07Rik | RIKEN cDNA 2310043J07 gene | 123.4 | 424.0 | 0.29 |
| 54409 | Ramp2 | receptor (calcitonin) activity modifying protein 2 | 183.2 | 626.9 | 0.29 |
| 18619 | Penk1 | preproenkephalin 1 | 115.5 | 393.0 | 0.29 |
| 64450 | Gpr85 | G protein-coupled receptor 85 | 14.8 | 50.0 | 0.30 |
| 436089 | LOC436089 | similar to matrilin 1, cartilage matrix protein | 64.3 | 217.3 | 0.30 |
| 21367 | Cntn2 | contactin 2 | 28.7 | 96.9 | 0.30 |
| 20183 | Rxrg | retinoid X receptor gamma | 186.5 | 629.5 | 0.30 |
| 16525 | Kcnk1 | potassium channel, subfamily K, member 1 | 371.7 | 1253.1 | 0.30 |
| 75429 | 3100002J23Rik | RIKEN cDNA 3100002J23 gene | 163.7 | 551.0 | 0.30 |
| 72121 | Dennd2d | DENN/MADD domain containing 2D | 44.8 | 150.2 | 0.30 |
| 227659 | Slc2a6 | solute carrier family 2 (facilitated glucose transporter), member 6 | 29.3 | 98.2 | 0.30 |
| 104601 | Mycbpap | Mycbp associated protein | 17.6 | 58.9 | 0.30 |
| 231134 | A930013K19Rik | RIKEN cDNA A930013K19 gene | 29.6 | 98.5 | 0.30 |
| 11474 | Actn3 | actinin alpha 3 | 990.4 | 3309.7 | 0.30 |
| 233651 | Dchs1 | dachsous 1 (Drosophila) | 16.4 | 54.5 | 0.30 |
| 71583 | 9130008F23Rik | RIKEN cDNA 9130008F23 gene | 117.3 | 390.8 | 0.30 |
| 18131 | Notch3 | Notch gene homolog 3 (Drosophila) | 1467.1 | 4870.9 | 0.30 |
| 20289 | Scx | scleraxis | 114.5 | 381.1 | 0.30 |
| 171166 | Mcoln3 | mucolipin 3 | 27.2 | 89.9 | 0.30 |
| 73235 | 3110082D06Rik | RIKEN cDNA 3110082D06 gene | 28.8 | 95.3 | 0.30 |
| 72185 | Dbndd1 | dysbindin (dystrobrevin binding protein 1) domain containing 1 | 203.9 | 670.7 | 0.30 |
| 20319 | Sfrp2 | secreted frizzled-related protein 2 | 478.2 | 1566.9 | 0.30 |
| 20363 | Sepp1 | selenoprotein P, plasma, 1 | 619.0 | 2015.6 | 0.31 |
| 18574 | Pde1b | phosphodiesterase 1B, Ca2+-calmodulin dependent | 258.2 | 843.3 | 0.31 |
| 24056 | Sh3bp5 | SH3-domain binding protein 5 (BTK-associated) | 48.2 | 157.4 | 0.31 |
| 24136 | Zfhx1b | zinc finger homeobox 1b | 42.1 | 136.4 | 0.31 |
| 20661 | Sort1 | sortilin 1 | 219.9 | 712.5 | 0.31 |
| 66451 | 2610528J11Rik | RIKEN cDNA 2610528J11 gene | 107.1 | 347.9 | 0.31 |
| 12023 | Barx2 | BarH-like homeobox 2 | 34.0 | 108.6 | 0.31 |
| 109151 | Chd9 | chromodomain helicase DNA binding protein 9 | 256.5 | 829.3 | 0.31 |
| 12293 | Cacna2d1 | calcium channel, voltage-dependent, alpha2/delta subunit 1 | 52.4 | 170.1 | 0.31 |
| 52552 | Parp8 | poly (ADP-ribose) polymerase family, member 8 | 38.8 | 125.0 | 0.31 |
| 12794 | Cnih2 | cornichon homolog 2 (Drosophila) | 551.3 | 1775.9 | 0.31 |
| 26877 | B3galt1 | UDP-Gal:betaGlcNAc beta 1,3-galactosyltransferase, polypeptide 1 | 22.3 | 71.6 | 0.31 |
| 53318 | Pdlim3 | PDZ and LIM domain 3 | 82.5 | 265.4 | 0.31 |
| 545015 | 2610042L04Rik | RIKEN cDNA 2610042L04 gene | 383.2 | 1232.7 | 0.31 |
| 56615 | Mgst1 | microsomal glutathione S-transferase 1 | 53.1 | 168.5 | 0.31 |
| 76980 | 3110006E14Rik | RIKEN cDNA 3110006E14 gene | 23.0 | 73.6 | 0.31 |
| 14527 | Gcgr | glucagon receptor | 37.6 | 120.6 | 0.31 |
| 69816 | 2010001M09Rik | RIKEN cDNA 2010001M09 gene | 14.6 | 46.8 | 0.31 |
| 14587 | Gfra3 | glial cell line derived neurotrophic factor family receptor alpha 3 | 57.1 | 182.7 | 0.31 |
| 102278 | Cpne7 | copine VII | 90.8 | 289.1 | 0.31 |
| 235472 | Prtg | protogenin homolog (Gallus gallus) | 948.1 | 3021.8 | 0.31 |
| 118449 | Synpo2 | synaptopodin 2 | 81.4 | 258.3 | 0.31 |
| 19253 | Ptpn18 | protein tyrosine phosphatase, non-receptor type 18 | 279.6 | 889.2 | 0.31 |
| 12577 | Cdkn1c | cyclin-dependent kinase inhibitor 1C (P57) | 1553.5 | 4940.3 | 0.31 |
| 216635 | Hbq1 | hemoglobin, theta 1 | 11.8 | 37.3 | 0.31 |
| 230971 | Megf6 | multiple EGF-like-domains 6 | 65.8 | 209.1 | 0.31 |
| 73251 | Setd7 | SET domain containing (lysine methyltransferase) 7 | 496.4 | 1571.8 | 0.32 |
| 73072 | BC068157 | cDNA sequence BC068157 | 37.3 | 117.2 | 0.32 |
| 211949 | Spsb4 | splA/ryanodine receptor domain and SOCS box containing 4 | 108.9 | 343.1 | 0.32 |
| 11624 | Ahrr | aryl-hydrocarbon receptor repressor | 26.0 | 80.3 | 0.32 |
| 67063 | 2810432L12Rik | RIKEN cDNA 2810432L12 gene | 291.6 | 914.3 | 0.32 |
| 69982 | Spink2 | serine peptidase inhibitor, Kazal type 2 | 64.9 | 203.5 | 0.32 |
| 71206 | 3110023G01Rik | RIKEN cDNA 3110023G01 gene | 141.8 | 444.2 | 0.32 |
| 12804 | Cntfr | ciliary neurotrophic factor receptor | 313.5 | 981.3 | 0.32 |
| 15208 | Hes5 | hairy and enhancer of split 5 (Drosophila) | 75.2 | 235.3 | 0.32 |
| 11982 | Atp10a | ATPase, class V, type 10A | 103.7 | 323.1 | 0.32 |
| 64337 | Gng13 | guanine nucleotide binding protein 13, gamma | 144.5 | 449.6 | 0.32 |
| 56461 | Kcnip3 | Kv channel interacting protein 3, calsenilin | 25.1 | 77.9 | 0.32 |
| 15944 | Irgm | immunity-related GTPase family, M | 126.2 | 391.9 | 0.32 |
| 223435 | Trio | triple functional domain (PTPRF interacting) | 533.1 | 1649.1 | 0.32 |
| 381319 | 9130211I03Rik | RIKEN cDNA 9130211I03 gene | 235.7 | 729.8 | 0.32 |
| 109205 | 5330439J01Rik | RIKEN cDNA 5330439J01 gene | 45.4 | 139.8 | 0.32 |
| 108655 | Foxp1 | forkhead box P1 | 1035.4 | 3201.0 | 0.32 |
| 216867 | Slc16a11 | solute carrier family 16 (monocarboxylic acid transporters), member 11 | 277.5 | 856.2 | 0.32 |
| 211623 | Plac9 | placenta specific 9 | 3249.0 | 9999.7 | 0.33 |
| 13435 | Dnmt3a | DNA methyltransferase 3A | 4588.9 | 13990.6 | 0.33 |
| 76459 | Car12 | carbonic anyhydrase 12 | 45.5 | 139.7 | 0.33 |
| 320472 | Ppm1e | protein phosphatase 1E (PP2C domain containing) | 21.7 | 66.3 | 0.33 |
| 330043 | 6430702L12 | hypothetical protein 6430702L12 | 171.2 | 523.8 | 0.33 |
| 170740 | Zfp287 | zinc finger protein 287 | 122.7 | 370.5 | 0.33 |
| 12889 | Cplx1 | complexin 1 | 41.0 | 124.9 | 0.33 |
| 93691 | Klf7 | Kruppel-like factor 7 (ubiquitous) | 498.1 | 1518.1 | 0.33 |
| 240444 | LOC240444 | similar to Potassium voltage-gated channel subfamily G member 2 (Voltage-gated potassium channel subunit Kv6.2) (Cardiac potassium channel subunit) | 19.1 | 58.1 | 0.33 |
| 67477 | 1300007F04Rik | RIKEN cDNA 1300007F04 gene | 19.9 | 60.2 | 0.33 |
| 63954 | Rbp7 | retinol binding protein 7, cellular | 56.8 | 169.7 | 0.33 |
| 94180 | Acsbg1 | acyl-CoA synthetase bubblegum family member 1 | 68.5 | 206.9 | 0.33 |
| 232237 | Fgd5 | FYVE, RhoGEF and PH domain containing 5 | 45.0 | 134.3 | 0.33 |
| 70377 | Derl3 | Der1-like domain family, member 3 | 247.7 | 748.5 | 0.33 |
| 675467 | LOC675467 | hypothetical protein LOC675467 | 154.9 | 459.8 | 0.33 |
| 21349 | Tal1 | T-cell acute lymphocytic leukemia 1 | 21.7 | 63.4 | 0.33 |
| 114479 | Slc5a5 | solute carrier family 5 (sodium iodide symporter), member 5 | 32.3 | 97.3 | 0.33 |
| 15407 | Hoxb1 | homeo box B1 | 26.4 | 79.6 | 0.33 |
| 194237 | BC057371 | cDNA sequence BC057371 | 379.3 | 1139.2 | 0.33 |
| 404337 | Olfr1383 | olfactory receptor 1383 | 21.1 | 62.8 | 0.33 |
| 13641 | Efnb1 | ephrin B1 | 518.9 | 1555.3 | 0.33 |
| 106042 | Prickle1 | prickle like 1 (Drosophila) | 534.4 | 1602.0 | 0.33 |
| 14582 | Gfi1b | growth factor independent 1B | 21.4 | 63.8 | 0.33 |
| 235604 | Camkv | CaM kinase-like vesicle-associated | 241.7 | 722.5 | 0.33 |
| 240725 | Sulf1 | sulfatase 1 | 906.2 | 2704.3 | 0.34 |
| 20349 | Sema3e | sema domain, immunoglobulin domain (Ig), short basic domain, secreted, (semaphorin) 3E | 222.7 | 657.9 | 0.34 |
| 11302 | Aatk | apoptosis-associated tyrosine kinase | 35.0 | 103.6 | 0.34 |
| 330485 | Tmem145 | transmembrane protein 145 | 495.2 | 1467.5 | 0.34 |
| 67937 | 5330410G16Rik | RIKEN cDNA 5330410G16 gene | 1575.2 | 4672.5 | 0.34 |
| 107684 | Coro2a | coronin, actin binding protein 2A | 31.3 | 92.9 | 0.34 |
| 280635 | Emilin3 | elastin microfibril interfacer 3 | 124.6 | 367.5 | 0.34 |
| 67865 | Rgs10 | regulator of G-protein signalling 10 | 208.4 | 614.7 | 0.34 |
| 56615 | Mgst1 | microsomal glutathione S-transferase 1 | 102.0 | 299.4 | 0.34 |
| 13527 | Dtna | dystrobrevin alpha | 56.3 | 164.9 | 0.34 |
| 223726 | Mpped1 | metallophosphoesterase domain containing 1 | 84.1 | 246.4 | 0.34 |
| 110835 | Chrna5 | cholinergic receptor, nicotinic, alpha polypeptide 5 | 17.3 | 50.2 | 0.34 |
| 215653 | Rassf2 | Ras association (RalGDS/AF-6) domain family 2 | 63.2 | 183.1 | 0.34 |
| 109676 | Ank2 | ankyrin 2, brain | 47.1 | 137.6 | 0.34 |
| 16912 | Psmb9 | proteosome (prosome, macropain) subunit, beta type 9 (large multifunctional peptidase 2) | 290.8 | 847.0 | 0.34 |
| 18549 | Pcsk2 | proprotein convertase subtilisin/kexin type 2 | 37.5 | 109.3 | 0.34 |
| 70784 | Rasl12 | RAS-like, family 12 | 129.9 | 377.6 | 0.34 |
| 55927 | Hes6 | hairy and enhancer of split 6 (Drosophila) | 3011.5 | 8750.1 | 0.34 |
| 24136 | Zfhx1b | zinc finger homeobox 1b | 19.7 | 57.0 | 0.34 |
| 18196 | Nsg1 | neuron specific gene family member 1 | 131.7 | 381.6 | 0.34 |
| 74760 | Rab3il1 | RAB3A interacting protein (rabin3)-like 1 | 64.2 | 184.9 | 0.35 |
| 59020 | Pdzk1 | PDZ domain containing 1 | 117.6 | 340.0 | 0.35 |
| 102153 | C230098O21Rik | RIKEN cDNA C230098O21 gene | 158.4 | 454.2 | 0.35 |
| 214931 | Fbxl16 | F-box and leucine-rich repeat protein 16 | 342.6 | 986.6 | 0.35 |
| 58208 | Bcl11b | B-cell leukemia/lymphoma 11B | 128.3 | 369.1 | 0.35 |
| 67092 | Gatm | glycine amidinotransferase (L-arginine:glycine amidinotransferase) | 52.6 | 151.1 | 0.35 |
| 20899 | Stra8 | stimulated by retinoic acid gene 8 | 15408.8 | 44187.1 | 0.35 |
| 242022 | Frem2 | Fras1 related extracellular matrix protein 2 | 124.5 | 356.0 | 0.35 |
| 74190 | 1200009I06Rik | RIKEN cDNA 1200009I06 gene | 142.9 | 409.8 | 0.35 |
| 14425 | Galnt3 | UDP-N-acetyl-alpha-D-galactosamine:polypeptide N-acetylgalactosaminyltransferase 3 | 204.1 | 584.4 | 0.35 |
| 12555 | Cdh15 | cadherin 15 | 32.1 | 91.9 | 0.35 |
| 70355 | Gprc5c | G protein-coupled receptor, family C, group 5, member C | 104.3 | 298.5 | 0.35 |
| 26897 | Acot1 | acyl-CoA thioesterase 1 | 2002.8 | 5731.1 | 0.35 |
| 73442 | Hspa12a | heat shock protein 12A | 16.0 | 45.6 | 0.35 |
| 76560 | Prss8 | protease, serine, 8 (prostasin) | 318.7 | 910.5 | 0.35 |
| 210741 | Kcnk12 | potassium channel, subfamily K, member 12 | 19.1 | 53.1 | 0.35 |
| 12904 | Crabp2 | cellular retinoic acid binding protein II | 17.9 | 50.8 | 0.35 |
| 15110 | Hand1 | heart and neural crest derivatives expressed transcript 1 | 17.0 | 48.1 | 0.35 |
| 109689 | Arrb1 | arrestin, beta 1 | 1381.0 | 3932.9 | 0.35 |
| 435965 | Lrp3 | low density lipoprotein receptor-related protein 3 | 140.0 | 398.4 | 0.35 |
| 192950 | AB182283 | cDNA sequence AB182283 | 20.3 | 57.4 | 0.35 |
| 68867 | Rnf122 | ring finger protein 122 | 104.8 | 297.4 | 0.35 |
| 230787 | BC013712 | cDNA sequence BC013712 | 254.1 | 721.3 | 0.35 |
| 105853 | Mal2 | mal, T-cell differentiation protein 2 | 169.5 | 478.6 | 0.35 |
| 67622 | 1810057P16Rik | RIKEN cDNA 1810057P16 gene | 358.2 | 1008.9 | 0.35 |
| 70676 | Gulp1 | GULP, engulfment adaptor PTB domain containing 1 | 139.0 | 393.3 | 0.35 |
| 73102 | 3110004L20Rik | RIKEN cDNA 3110004L20 gene | 353.8 | 1000.4 | 0.35 |
| 55932 | Gbp4 | guanylate nucleotide binding protein 4 | 52.1 | 146.2 | 0.35 |
| 216739 | Acsl6 | acyl-CoA synthetase long-chain family member 6 | 38.2 | 107.0 | 0.35 |
| 20675 | Sox3 | SRY-box containing gene 3 | 23.0 | 64.7 | 0.35 |
| 76884 | Cyfip2 | cytoplasmic FMR1 interacting protein 2 | 267.5 | 753.5 | 0.35 |
| 73246 | Rassf6 | Ras association (RalGDS/AF-6) domain family 6 | 18.7 | 52.6 | 0.35 |
| 17263 | Gtl2 | GTL2, imprinted maternally expressed untranslated mRNA | 222.5 | 623.3 | 0.36 |
| 215723 | BC024997 | cDNA sequence BC024997 | 31.2 | 87.2 | 0.36 |
| 64685 | Nmi | N-myc (and STAT) interactor | 413.3 | 1158.6 | 0.36 |
| 208777 | Sned1 | sushi, nidogen and EGF-like domains 1 | 25.0 | 70.1 | 0.36 |
| 319832 | 6332401O19Rik | RIKEN cDNA 6332401O19 gene | 14.6 | 40.8 | 0.36 |
| 108978 | 4930555G01Rik | RIKEN cDNA 4930555G01 gene | 36.7 | 101.0 | 0.36 |
| 71703 | Armcx3 | armadillo repeat containing, X-linked 3 | 132.8 | 369.5 | 0.36 |
| 57257 | Vav3 | vav 3 oncogene | 28.5 | 79.6 | 0.36 |
| 76574 | Mfsd2 | major facilitator superfamily domain containing 2 | 436.1 | 1216.5 | 0.36 |
| 237504 | Pamci | peptidylglycine alpha-amidating monooxygenase COOH-terminal interactor | 17.2 | 47.2 | 0.36 |
| 102566 | Tmem16k | transmembrane protein 16K | 103.3 | 287.4 | 0.36 |
| 72293 | Nkd2 | naked cuticle 2 homolog (Drosophila) | 205.6 | 572.0 | 0.36 |
| 15571 | Elavl3 | ELAV (embryonic lethal, abnormal vision, Drosophila)-like 3 (Hu antigen C) | 520.4 | 1443.0 | 0.36 |
| 20350 | Sema3f | sema domain, immunoglobulin domain (Ig), short basic domain, secreted, (semaphorin) 3 F | 23.1 | 64.1 | 0.36 |
| 22141 | Tub | tubby candidate gene | 261.1 | 723.3 | 0.36 |
| 101490 | Inpp5f | inositol polyphosphate-5-phosphatase F | 279.9 | 775.6 | 0.36 |
| 24088 | Tlr2 | toll-like receptor 2 | 107.0 | 296.5 | 0.36 |
| 217219 | BC025575 | cDNA sequence BC025575 | 947.4 | 2622.1 | 0.36 |
| 19088 | Prkar2b | protein kinase, cAMP dependent regulatory, type II beta | 425.3 | 1170.3 | 0.36 |
| 208076 | Pknox2 | Pbx/knotted 1 homeobox 2 | 29.1 | 79.0 | 0.36 |
| 11925 | Neurog3 | neurogenin 3 | 33.0 | 89.6 | 0.36 |
| 235130 | Adamts15 | a disintegrin-like and metallopeptidase (reprolysin type) with thrombospondin type 1 motif, 15 | 63.9 | 172.9 | 0.36 |
| 14451 | Gas1 | growth arrest specific 1 | 39.2 | 107.3 | 0.36 |
| 14704 | Gng3 | guanine nucleotide binding protein (G protein), gamma 3 subunit | 1583.6 | 4335.4 | 0.37 |
| 19073 | Prg1 | proteoglycan 1, secretory granule | 54.4 | 147.9 | 0.37 |
| 18481 | Pak3 | p21 (CDKN1A)-activated kinase 3 | 18.8 | 51.1 | 0.37 |
| 18755 | Prkch | protein kinase C, eta | 21.6 | 58.9 | 0.37 |
| 20358 | Sema6a | sema domain, transmembrane domain (TM), and cytoplasmic domain, (semaphorin) 6A | 31.7 | 86.3 | 0.37 |
| 69638 | 2310040A07Rik | RIKEN cDNA 2310040A07 gene | 621.9 | 1694.2 | 0.37 |
| 629974 | D11Ertd759e | DNA segment, Chr 11, ERATO Doi 759, expressed | 444.2 | 1207.5 | 0.37 |
| 19273 | Ptpru | protein tyrosine phosphatase, receptor type, U | 158.8 | 431.7 | 0.37 |
| 98682 | 2210010L05Rik | RIKEN cDNA 2210010L05 gene | 38.1 | 103.6 | 0.37 |
| 21955 | Tnnt1 | troponin T1, skeletal, slow | 706.4 | 1917.9 | 0.37 |
| 208117 | Aph1b | anterior pharynx defective 1b homolog (C. elegans) | 52.0 | 141.1 | 0.37 |
| 320827 | C530008M17Rik | RIKEN cDNA C530008M17 gene | 85.1 | 230.9 | 0.37 |
| 217558 | 6030408C04Rik | RIKEN cDNA 6030408C04 gene | 702.6 | 1906.0 | 0.37 |
| 332937 | Tcfap2e | transcription factor AP-2, epsilon | 39.3 | 105.3 | 0.37 |
| 75607 | Wnk2 | WNK lysine deficient protein kinase 2 | 57.5 | 155.7 | 0.37 |
| 72434 | Lypd3 | Ly6/Plaur domain containing 3 | 42.6 | 115.0 | 0.37 |
| 60527 | Fads3 | fatty acid desaturase 3 | 161.4 | 434.9 | 0.37 |
| 20353 | Sema4c | sema domain, immunoglobulin domain (Ig), transmembrane domain (TM) and short cytoplasmic domain, (semaphorin) 4C | 230.5 | 621.3 | 0.37 |
| 27385 | Magel2 | melanoma antigen, family L, 2 | 79.4 | 212.9 | 0.37 |
| 67473 | 1300013J15Rik | RIKEN cDNA 1300013J15 gene | 178.9 | 479.4 | 0.37 |
| 97086 | C80638 | expressed sequence C80638 | 120.4 | 323.1 | 0.37 |
| 56177 | Olfm1 | olfactomedin 1 | 391.1 | 1049.9 | 0.37 |
| 230249 | AI314180 | expressed sequence AI314180 | 35.5 | 95.2 | 0.37 |
| 320982 | Arl4c | ADP-ribosylation factor-like 4C | 4975.0 | 13352.1 | 0.37 |
| 328644 | LOC328644 | hypothetical gene supported by AK045595 | 17.0 | 45.5 | 0.37 |
| 109857 | Cbr3 | carbonyl reductase 3 | 2970.6 | 7963.4 | 0.37 |
| 78560 | Gpr124 | G protein-coupled receptor 124 | 315.3 | 838.0 | 0.37 |
| 20148 | Dhrs3 | dehydrogenase/reductase (SDR family) member 3 | 104.8 | 278.5 | 0.37 |
| 15376 | Foxa2 | forkhead box A2 | 39.9 | 106.3 | 0.37 |
| 11876 | Artn | artemin | 20.9 | 55.3 | 0.37 |
| 72898 | Asphd2 | aspartate beta-hydroxylase domain containing 2 | 1298.3 | 3455.1 | 0.38 |
| 21810 | Tgfbi | transforming growth factor, beta induced | 394.4 | 1050.7 | 0.38 |
| 228880 | Prkcbp1 | protein kinase C binding protein 1 | 1306.9 | 3481.4 | 0.38 |
| 14600 | Ghr | growth hormone receptor | 69.6 | 185.1 | 0.38 |
| 76770 | 2010005H15Rik | RIKEN cDNA 2010005H15 gene | 22.3 | 59.3 | 0.38 |
| 17095 | Lyl1 | lymphoblastomic leukemia | 64.8 | 172.1 | 0.38 |
| 14421 | B4galnt1 | beta-1,4-N-acetyl-galactosaminyl transferase 1 | 206.5 | 547.1 | 0.38 |
| 13384 | Mpp3 | membrane protein, palmitoylated 3 (MAGUK p55 subfamily member 3) | 99.1 | 262.8 | 0.38 |
| 12475 | Cd14 | CD14 antigen | 86.1 | 227.8 | 0.38 |
| 17259 | Mef2b | myocyte enhancer factor 2B | 374.1 | 985.9 | 0.38 |
| 13386 | Dlk1 | delta-like 1 homolog (Drosophila) | 302.2 | 795.4 | 0.38 |
| 76933 | Ifi27 | interferon, alpha-inducible protein 27 | 18.6 | 48.2 | 0.38 |
| 14605 | Tsc22d3 | TSC22 domain family 3 | 160.0 | 420.6 | 0.38 |
| 76566 | 1500005K14Rik | RIKEN cDNA 1500005K14 gene | 24.7 | 64.7 | 0.38 |
| 75745 | Rian | RNA imprinted and accumulated in nucleus | 297.8 | 767.5 | 0.38 |
| 380753 | Atxn7l1 | ataxin 7-like 1 | 20.3 | 53.2 | 0.38 |
| 108816 | 4933409K07Rik | RIKEN cDNA 4933409K07 gene | 48.9 | 128.1 | 0.38 |
| 12695 | Inadl | InaD-like (Drosophila) | 168.0 | 440.9 | 0.38 |
| 12125 | Bcl2l11 | BCL2-like 11 (apoptosis facilitator) | 1093.0 | 2854.5 | 0.38 |
| 319757 | Smo | smoothened homolog (Drosophila) | 4362.4 | 11397.9 | 0.38 |
| 57319 | Smpdl3a | sphingomyelin phosphodiesterase, acid-like 3A | 26.4 | 68.7 | 0.38 |
| 669759 | LOC669759 | similar to putative retrovirus-related gag protein | 32.3 | 83.5 | 0.38 |
| 237387 | Lrrc3 | leucine rich repeat containing 3 | 89.8 | 232.9 | 0.38 |
| 14345 | Fut4 | fucosyltransferase 4 | 30.8 | 79.7 | 0.38 |
| 75178 | 4930528F23Rik | RIKEN cDNA 4930528F23 gene | 46.3 | 120.1 | 0.38 |
| 57265 | Fzd2 | frizzled homolog 2 (Drosophila) | 1004.8 | 2613.0 | 0.38 |
| 24084 | Tekt2 | tektin 2 | 217.2 | 564.1 | 0.38 |
| 21354 | Tap1 | transporter 1, ATP-binding cassette, sub-family B (MDR/TAP) | 55.8 | 145.0 | 0.38 |
| 70571 | Tcerg1l | transcription elongation regulator 1-like | 62.5 | 159.5 | 0.39 |
| 226841 | Vash2 | vasohibin 2 | 149.7 | 387.8 | 0.39 |
| 433256 | Acsl5 | acyl-CoA synthetase long-chain family member 5 | 477.1 | 1233.9 | 0.39 |
| 13078 | Cyp1b1 | cytochrome P450, family 1, subfamily b, polypeptide 1 | 31.6 | 81.3 | 0.39 |
| 50873 | Park2 | parkin | 125.1 | 322.8 | 0.39 |
| 19735 | Rgs2 | regulator of G-protein signaling 2 | 170.7 | 437.2 | 0.39 |
| 24115 | Vmd2 | vitelliform macular dystrophy 2 homolog (human) | 68.6 | 175.1 | 0.39 |
| 12933 | Crmp1 | collapsin response mediator protein 1 | 1739.8 | 4465.6 | 0.39 |
| 103743 | Tmem98 | transmembrane protein 98 | 297.6 | 763.7 | 0.39 |
| 226180 | Ina | internexin neuronal intermediate filament protein, alpha | 530.3 | 1361.2 | 0.39 |
| 56741 | Nope | neighbor of Punc E11 | 109.1 | 278.2 | 0.39 |
| 80898 | Arts1 | type 1 tumor necrosis factor receptor shedding aminopeptidase regulator | 95.3 | 244.1 | 0.39 |
| 18516 | Pbx3 | pre B-cell leukemia transcription factor 3 | 460.7 | 1181.2 | 0.39 |
| 72475 | Ssbp3 | single-stranded DNA binding protein 3 | 2484.2 | 6339.5 | 0.39 |
| 620807 | LOC620807 | similar to Major urinary protein 4 precursor (MUP 4) | 76.9 | 196.1 | 0.39 |
| 12745 | Clgn | calmegin | 343.0 | 874.7 | 0.39 |
| 58200 | Ppp1r1a | protein phosphatase 1, regulatory (inhibitor) subunit 1A | 1210.9 | 3093.3 | 0.39 |
| 11864 | Arnt2 | aryl hydrocarbon receptor nuclear translocator 2 | 40.9 | 102.7 | 0.39 |
| 16009 | Igfbp3 | insulin-like growth factor binding protein 3 | 192.6 | 489.1 | 0.39 |
| 13446 | Doc2a | double C2, alpha | 211.8 | 540.4 | 0.39 |
| 215113 | Slc43a2 | solute carrier family 43, member 2 | 23.3 | 58.9 | 0.39 |
| 70082 | Lysmd2 | LysM, putative peptidoglycan-binding, domain containing 2 | 395.3 | 1005.5 | 0.39 |
| 208666 | Diras1 | DIRAS family, GTP-binding RAS-like 1 | 182.8 | 465.4 | 0.39 |
| 230603 | 4922503N01Rik | RIKEN cDNA 4922503N01 gene | 55.9 | 142.1 | 0.39 |
| 17954 | Nap1l2 | nucleosome assembly protein 1-like 2 | 61.8 | 155.7 | 0.39 |
| 18211 | Ntrk1 | neurotrophic tyrosine kinase, receptor, type 1 | 39.5 | 99.8 | 0.39 |
| 55983 | Pdzrn3 | PDZ domain containing RING finger 3 | 164.9 | 416.4 | 0.39 |
| 12931 | Crlf1 | cytokine receptor-like factor 1 | 489.5 | 1241.2 | 0.39 |
| 20513 | Slc1a6 | solute carrier family 1 (high affinity aspartate/glutamate transporter), member 6 | 116.1 | 291.9 | 0.40 |
| 19734 | Rgs16 | regulator of G-protein signaling 16 | 170.3 | 428.6 | 0.40 |
| 20677 | Sox4 | SRY-box containing gene 4 | 824.3 | 2046.2 | 0.40 |
| 58909 | D430015B01Rik | RIKEN cDNA D430015B01 gene | 25.7 | 63.9 | 0.40 |
| 192663 | Abcg4 | ATP-binding cassette, sub-family G (WHITE), member 4 | 28.0 | 69.4 | 0.40 |
| 53606 | Isg15 | ISG15 ubiquitin-like modifier | 25.8 | 64.5 | 0.40 |
| 171180 | Syt12 | synaptotagmin XII | 29.5 | 73.8 | 0.40 |
| 20855 | Stc1 | stanniocalcin 1 | 39.9 | 99.8 | 0.40 |
| 194388 | D230004J03Rik | RIKEN cDNA D230004J03 gene | 66.4 | 164.1 | 0.40 |
| 21925 | Tnnc2 | troponin C2, fast | 294.6 | 734.2 | 0.40 |
| 15572 | Elavl4 | ELAV (embryonic lethal, abnormal vision, Drosophila)-like 4 (Hu antigen D) | 49.5 | 123.7 | 0.40 |
| 207777 | Bzrap1 | benzodiazapine receptor associated protein 1 | 213.0 | 532.8 | 0.40 |
| 14809 | Grik5 | glutamate receptor, ionotropic, kainate 5 (gamma 2) | 156.8 | 391.2 | 0.40 |
| 15408 | Hoxb13 | homeo box B13 | 99.7 | 247.7 | 0.40 |
| 544988 | LOC544988 | hypothetical protein LOC544988 | 365.4 | 904.6 | 0.40 |
| 67874 | Rprm | reprimo, TP53 dependent G2 arrest mediator candidate | 197.9 | 493.1 | 0.40 |
| 17888 | Myh6 | myosin, heavy polypeptide 6, cardiac muscle, alpha | 129.7 | 322.5 | 0.40 |
| 11513 | Adcy7 | adenylate cyclase 7 | 80.0 | 197.6 | 0.40 |
| 19206 | Ptch1 | patched homolog 1 | 57.1 | 141.7 | 0.40 |
| 21928 | Tnfaip2 | tumor necrosis factor, alpha-induced protein 2 | 60.4 | 149.3 | 0.40 |
| 207495 | Baiap2l2 | BAI1-associated protein 2-like 2 | 44.8 | 110.4 | 0.40 |
| 12721 | Coro1a | coronin, actin binding protein 1A | 572.8 | 1414.7 | 0.40 |
| 78317 | Ccdc88 | coiled-coil domain containing 88 | 519.4 | 1280.7 | 0.40 |
| 107769 | Tm6sf1 | transmembrane 6 superfamily member 1 | 21.9 | 54.0 | 0.40 |
| 217198 | Plekhh3 | pleckstrin homology domain containing, family H (with MyTH4 domain) member 3 | 583.3 | 1438.6 | 0.40 |
| 20743 | Spnb3 | spectrin beta 3 | 253.0 | 624.9 | 0.40 |
| 107022 | Gramd3 | GRAM domain containing 3 | 299.0 | 735.3 | 0.40 |
| 69824 | 2010001H14Rik | RIKEN cDNA 2010001H14 gene | 300.0 | 739.9 | 0.41 |
| 76686 | 1500005P14Rik | RIKEN cDNA 1500005P14 gene | 149.1 | 367.8 | 0.41 |
| 11628 | Aicda | activation-induced cytidine deaminase | 26.6 | 64.5 | 0.41 |
| 71683 | Gypc | glycophorin C | 36.5 | 89.6 | 0.41 |
| 73173 | Pcdh18 | protocadherin 18 | 19.5 | 47.4 | 0.41 |
| 231858 | D930005D10Rik | RIKEN cDNA D930005D10 gene | 243.5 | 586.4 | 0.41 |
| 269116 | Nfasc | neurofascin | 21.3 | 51.9 | 0.41 |
| 78408 | 2900046G09Rik | RIKEN cDNA 2900046G09 gene | 174.2 | 427.5 | 0.41 |
| 103712 | 6330403K07Rik | RIKEN cDNA 6330403K07 gene | 280.1 | 687.6 | 0.41 |
| 232943 | Klc3 | kinesin light chain 3 | 1036.0 | 2539.7 | 0.41 |
| 231440 | 9130213B05Rik | RIKEN cDNA 9130213B05 gene | 195.7 | 478.1 | 0.41 |
| 26565 | Pla2g10 | phospholipase A2, group X | 428.5 | 1034.9 | 0.41 |
| 13656 | Egr4 | early growth response 4 | 168.4 | 410.4 | 0.41 |
| 320683 | Zfp629 | zinc finger protein 629 | 27.7 | 67.6 | 0.41 |
| 69047 | 1810010G06Rik | RIKEN cDNA 1810010G06 gene | 28.1 | 68.0 | 0.41 |
| 16190 | Il4ra | interleukin 4 receptor, alpha | 79.2 | 193.3 | 0.41 |
| 432720 | Akr1c19 | aldo-keto reductase family 1, member C19 | 33.0 | 80.4 | 0.41 |
| 58522 | Trim54 | tripartite motif-containing 54 | 558.1 | 1360.3 | 0.41 |
| 330319 | BQ952480 | expressed sequence BQ952480 | 198.3 | 482.2 | 0.41 |
| 231832 | BC019731 | cDNA sequence BC019731 | 260.7 | 633.3 | 0.41 |
| 11732 | Ank | progressive ankylosis | 208.8 | 506.3 | 0.41 |
| 330369 | Fbxo41 | F-box protein 41 | 26.7 | 64.3 | 0.41 |
| 16574 | Kif5c | kinesin family member 5C | 860.9 | 2090.5 | 0.41 |
| 68519 | Eml1 | echinoderm microtubule associated protein like 1 | 359.7 | 868.3 | 0.41 |
| 213783 | Plekhg1 | pleckstrin homology domain containing, family G (with RhoGef domain) member 1 | 126.9 | 305.5 | 0.41 |
| 112407 | Egln3 | EGL nine homolog 3 (C. elegans) | 1396.5 | 3365.9 | 0.41 |
| 68895 | Rasl11a | RAS-like, family 11, member A | 108.0 | 259.8 | 0.41 |
| 69993 | Chn2 | chimerin (chimaerin) 2 | 65.9 | 158.5 | 0.42 |
| 107528 | Magee1 | melanoma antigen, family E, 1 | 33.2 | 80.0 | 0.42 |
| 56437 | Rrad | Ras-related associated with diabetes | 51.6 | 124.1 | 0.42 |
| 15258 | Hipk2 | homeodomain interacting protein kinase 2 | 210.6 | 505.1 | 0.42 |
| 15206 | Hes2 | hairy and enhancer of split 2 (Drosophila) | 43.1 | 101.9 | 0.42 |
| 320706 | 9830001H06Rik | RIKEN cDNA 9830001H06 gene | 704.0 | 1690.4 | 0.42 |
| 21390 | Tbxa2r | thromboxane A2 receptor | 87.1 | 208.8 | 0.42 |
| 67621 | 2310026E23Rik | RIKEN cDNA 2310026E23 gene | 301.6 | 723.9 | 0.42 |
| 13004 | Cspg3 | chondroitin sulfate proteoglycan 3 | 458.8 | 1099.0 | 0.42 |
| 56089 | Ramp3 | receptor (calcitonin) activity modifying protein 3 | 435.7 | 1044.2 | 0.42 |
| 241556 | Tspan18 | tetraspanin 18 | 24.1 | 57.1 | 0.42 |
| 68166 | Spire1 | spire homolog 1 (Drosophila) | 59.5 | 141.7 | 0.42 |
| 18008 | Nes | nestin | 1524.0 | 3644.4 | 0.42 |
| 14778 | Gpx3 | glutathione peroxidase 3 | 171.6 | 409.6 | 0.42 |
| 328643 | BC052055 | cDNA sequence BC052055 | 31.5 | 74.5 | 0.42 |
| 20617 | Snca | synuclein, alpha | 98.0 | 230.2 | 0.42 |
| 52331 | D5Ertd593e | DNA segment, Chr 5, ERATO Doi 593, expressed | 81.6 | 193.6 | 0.42 |
| 673676 | LOC673676 | hypothetical protein LOC673676 | 209.3 | 495.5 | 0.42 |
| 114643 | Oas1c | 2'-5' oligoadenylate synthetase 1C | 120.2 | 285.1 | 0.42 |
| 27278 | Clnk | cytokine-dependent hematopoietic cell linker | 38.5 | 91.5 | 0.42 |
| 14788 | Gpr162 | G protein-coupled receptor 162 | 337.9 | 802.4 | 0.42 |
| 22239 | Ugt8a | UDP galactosyltransferase 8A | 45.1 | 107.0 | 0.42 |
| 66039 | D14Ertd449e | DNA segment, Chr 14, ERATO Doi 449, expressed | 2508.2 | 5944.0 | 0.42 |
| 207607 | Ccdc40 | coiled-coil domain containing 40 | 460.0 | 1089.3 | 0.42 |
| 98845 | Eps8l2 | EPS8-like 2 | 52.5 | 123.9 | 0.42 |
| 15371 | Hmx1 | H6 homeo box 1 | 77.4 | 183.1 | 0.42 |
| 66889 | Rnf128 | ring finger protein 128 | 254.6 | 601.8 | 0.42 |
| 64339 | Fndc4 | fibronectin type III domain containing 4 | 162.5 | 383.7 | 0.42 |
| 381738 | Gm1060 | gene model 1060, (NCBI) | 292.7 | 690.6 | 0.42 |
| 244418 | D8Ertd82e | DNA segment, Chr 8, ERATO Doi 82, expressed | 215.5 | 507.5 | 0.42 |
| 69938 | Scrn1 | secernin 1 | 445.9 | 1050.7 | 0.42 |
| 14866 | Gstm5 | glutathione S-transferase, mu 5 | 643.4 | 1514.4 | 0.42 |
| 67263 | Zswim6 | zinc finger, SWIM domain containing 6 | 307.6 | 723.4 | 0.42 |
| 78887 | Sfi1 | Sfi1 homolog, spindle assembly associated (yeast) | 153.6 | 361.8 | 0.42 |
| 17390 | Mmp2 | matrix metallopeptidase 2 | 163.7 | 385.4 | 0.42 |
| 100604 | Lrrc8c | leucine rich repeat containing 8 family, member C | 59.4 | 139.2 | 0.43 |
| 15893 | Ica1 | islet cell autoantigen 1 | 149.4 | 348.5 | 0.43 |
| 338523 | A630082K20Rik | RIKEN cDNA A630082K20 gene | 27.2 | 63.1 | 0.43 |
| 98932 | Myl9 | myosin, light polypeptide 9, regulatory | 1855.5 | 4355.1 | 0.43 |
| 20536 | Slc4a3 | solute carrier family 4 (anion exchanger), member 3 | 137.4 | 321.0 | 0.43 |
| 68842 | Tulp4 | tubby like protein 4 | 373.2 | 873.1 | 0.43 |
| 75426 | Igfbpl1 | insulin-like growth factor binding protein-like 1 | 23.8 | 55.5 | 0.43 |
| 20811 | Srms | src-related kinase lacking C-terminal regulatory tyrosine and N-terminal myristylation sites | 26.4 | 61.9 | 0.43 |
| 236920 | Stard8 | START domain containing 8 | 50.4 | 117.7 | 0.43 |
| 71912 | Jsrp1 | junctional sarcoplasmic reticulum protein 1 | 47.0 | 109.7 | 0.43 |
| 68480 | 1110007C09Rik | RIKEN cDNA 1110007C09 gene | 1363.3 | 3190.6 | 0.43 |
| 105653 | Phyhip | phytanoyl-CoA hydroxylase interacting protein | 84.8 | 198.1 | 0.43 |
| 208795 | Tmem63a | transmembrane protein 63a | 889.9 | 2074.7 | 0.43 |
| 56745 | C1qtnf1 | C1q and tumor necrosis factor related protein 1 | 19.2 | 44.1 | 0.43 |
| 13436 | Dnmt3b | DNA methyltransferase 3B | 12094.6 | 28130.8 | 0.43 |
| 73296 | Rhobtb3 | Rho-related BTB domain containing 3 | 280.3 | 650.8 | 0.43 |
| 20482 | Skil | SKI-like | 1290.6 | 2986.2 | 0.43 |
| 73608 | Marveld3 | MARVEL (membrane-associating) domain containing 3 | 86.8 | 201.2 | 0.43 |
| 100121 | Tdrd7 | tudor domain containing 7 | 100.1 | 231.7 | 0.43 |
| 18591 | Pdgfb | platelet derived growth factor, B polypeptide | 45.4 | 104.5 | 0.43 |
| 80909 | Gats | opposite strand transcription unit to Stag3 | 79.3 | 183.0 | 0.43 |
| 320799 | Zhx3 | zinc fingers and homeoboxes 3 | 34.6 | 79.1 | 0.43 |
| 11790 | Speg | SPEG complex locus | 565.2 | 1303.2 | 0.43 |
| 384309 | Trim56 | tripartite motif-containing 56 | 113.5 | 261.2 | 0.43 |
| 68655 | Fndc1 | fibronectin type III domain containing 1 | 123.2 | 283.8 | 0.43 |
| 18576 | Pde3b | phosphodiesterase 3B, cGMP-inhibited | 46.8 | 107.2 | 0.43 |
| 11689 | Alox5 | arachidonate 5-lipoxygenase | 67.4 | 155.2 | 0.43 |
| 76820 | D12Ertd553e | DNA segment, Chr 12, ERATO Doi 553, expressed | 32.5 | 74.2 | 0.43 |
| 12741 | Cldn5 | claudin 5 | 125.5 | 287.3 | 0.43 |
| 12824 | Col2a1 | procollagen, type II, alpha 1 | 174.0 | 400.1 | 0.43 |
| 23882 | Gadd45g | growth arrest and DNA-damage-inducible 45 gamma | 3904.9 | 8981.5 | 0.43 |
| 385109 | Gm1499 | gene model 1499, (NCBI) | 35.6 | 81.5 | 0.44 |
| 225631 | Onecut2 | one cut domain, family member 2 | 17.4 | 39.8 | 0.44 |
| 66991 | 2410004A20Rik | RIKEN cDNA 2410004A20 gene | 1696.0 | 3889.9 | 0.44 |
| 272723 | NP_TR6JSE50FPA | olfactory receptor NP_TR6JSE50FPA | 30.6 | 70.3 | 0.44 |
| 212326 | BC035537 | cDNA sequence BC035537 | 105.3 | 240.2 | 0.44 |
| 233071 | Snx26 | sorting nexin 26 | 31.0 | 70.2 | 0.44 |
| 74048 | 4632428N05Rik | RIKEN cDNA 4632428N05 gene | 39.4 | 90.2 | 0.44 |
| 58226 | Cacna1h | calcium channel, voltage-dependent, T type, alpha 1H subunit | 17.5 | 40.0 | 0.44 |
| 12043 | Bcl2 | B-cell leukemia/lymphoma 2 | 101.4 | 231.7 | 0.44 |
| 12266 | C3 | complement component 3 | 52.1 | 119.1 | 0.44 |
| 74185 | Gbe1 | glucan (1,4-alpha-), branching enzyme 1 | 716.7 | 1634.5 | 0.44 |
| 22644 | Rnf103 | ring finger protein 103 | 97.6 | 221.5 | 0.44 |
| 69239 | 2610034M16Rik | RIKEN cDNA 2610034M16 gene | 32.3 | 73.4 | 0.44 |
| 75438 | 1700001E04Rik | RIKEN cDNA 1700001E04 gene | 165.1 | 375.8 | 0.44 |
| 11944 | Atp4a | ATPase, H+/K+ exchanging, gastric, alpha polypeptide | 28.4 | 63.8 | 0.44 |
| 14632 | Gli1 | GLI-Kruppel family member GLI1 | 61.5 | 140.1 | 0.44 |
| 235406 | Sh3px3 | SH3 and PX domain containing 3 | 91.0 | 207.2 | 0.44 |
| 16524 | Kcnj9 | potassium inwardly-rectifying channel, subfamily J, member 9 | 31.7 | 71.7 | 0.44 |
| 18741 | Pitx2 | paired-like homeodomain transcription factor 2 | 281.3 | 637.5 | 0.44 |
| 20449 | St8sia1 | ST8 alpha-N-acetyl-neuraminide alpha-2,8-sialyltransferase 1 | 25.6 | 58.1 | 0.44 |
| 65956 | Ccl21c | chemokine (C-C motif) ligand 21c (leucine) | 20.2 | 45.5 | 0.44 |
| 67844 | Rab32 | RAB32, member RAS oncogene family | 24.6 | 55.3 | 0.44 |
| 230126 | Shb | src homology 2 domain-containing transforming protein B | 196.4 | 445.0 | 0.44 |
| 20713 | Serpini1 | serine (or cysteine) peptidase inhibitor, clade I, member 1 | 238.2 | 539.5 | 0.44 |
| 219151 | Scara3 | scavenger receptor class A, member 3 | 16.3 | 36.7 | 0.44 |
| 67839 | Gpsm1 | G-protein signalling modulator 1 (AGS3-like, C. elegans) | 1957.3 | 4429.2 | 0.44 |
| 108978 | 4930555G01Rik | RIKEN cDNA 4930555G01 gene | 287.5 | 647.6 | 0.44 |
| 218772 | Rarb | retinoic acid receptor, beta | 71.8 | 162.3 | 0.44 |
| 12608 | Cebpb | CCAAT/enhancer binding protein (C/EBP), beta | 1302.0 | 2939.2 | 0.44 |
| 18739 | Pitpnm1 | phosphatidylinositol membrane-associated 1 | 887.9 | 2008.8 | 0.44 |
| 73234 | 3110079O15Rik | RIKEN cDNA 3110079O15 gene | 21.3 | 48.1 | 0.44 |
| 26557 | Homer2 | homer homolog 2 (Drosophila) | 238.5 | 537.9 | 0.44 |
| 12490 | Cd34 | CD34 antigen | 301.9 | 680.5 | 0.44 |
| 66532 | 2210417D09Rik | RIKEN cDNA 2210417D09 gene | 68.7 | 155.0 | 0.44 |
| 22063 | Trpc1 | transient receptor potential cation channel, subfamily C, member 1 | 36.5 | 82.4 | 0.44 |
| 246104 | Rhbdl3 | rhomboid, veinlet-like 3 (Drosophila) | 19.6 | 44.3 | 0.44 |
| 28042 | D5Wsu178e | DNA segment, Chr 5, Wayne State University 178, expressed | 669.7 | 1506.9 | 0.44 |
| 22359 | Vldlr | very low density lipoprotein receptor | 388.8 | 873.3 | 0.44 |
| 192199 | Rspo1 | R-spondin homolog (Xenopus laevis) | 823.1 | 1846.8 | 0.45 |
| 72088 | Ush1c | Usher syndrome 1C homolog (human) | 47.3 | 106.0 | 0.45 |
| 93835 | Amn | amnionless | 247.4 | 555.3 | 0.45 |
| 75860 | 4930588N13Rik | RIKEN cDNA 4930588N13 gene | 22.3 | 49.9 | 0.45 |
| 108760 | Galntl1 | UDP-N-acetyl-alpha-D-galactosamine:polypeptide N-acetylgalactosaminyltransferase-like 1 | 157.9 | 354.3 | 0.45 |
| 67803 | Limd2 | LIM domain containing 2 | 11638.3 | 26091.4 | 0.45 |
| 53883 | Celsr2 | cadherin EGF LAG seven-pass G-type receptor 2 | 85.7 | 191.9 | 0.45 |
| 23849 | Klf6 | Kruppel-like factor 6 | 1463.7 | 3263.6 | 0.45 |
| 67731 | Fbxo32 | F-box only protein 32 | 60.3 | 133.3 | 0.45 |
| 14761 | Gpr27 | G protein-coupled receptor 27 | 38.6 | 86.2 | 0.45 |
| 14299 | Freq | frequenin homolog (Drosophila) | 30.2 | 67.4 | 0.45 |
| 207920 | Rbm35a | RNA binding motif protein 35A | 234.5 | 523.6 | 0.45 |
| 16145 | Igtp | interferon gamma induced GTPase | 209.8 | 467.7 | 0.45 |
| 320790 | Chd7 | chromodomain helicase DNA binding protein 7 | 7389.9 | 16494.6 | 0.45 |
| 20739 | Spna1 | spectrin alpha 1 | 87.4 | 194.7 | 0.45 |
| 20471 | Six1 | sine oculis-related homeobox 1 homolog (Drosophila) | 173.2 | 384.3 | 0.45 |
| 259302 | Srgap3 | SLIT-ROBO Rho GTPase activating protein 3 | 146.4 | 324.1 | 0.45 |
| 74519 | Cyp2j9 | cytochrome P450, family 2, subfamily j, polypeptide 9 | 26.0 | 57.6 | 0.45 |
| 621603 | Aldh3b2 | aldehyde dehydrogenase 3 family, member B2 | 58.5 | 130.1 | 0.45 |
| 22033 | Traf5 | Tnf receptor-associated factor 5 | 71.3 | 158.3 | 0.45 |
| 27357 | Gyg | glycogenin | 1609.5 | 3577.7 | 0.45 |
| 19883 | Rora | RAR-related orphan receptor alpha | 54.4 | 121.0 | 0.45 |
| 105298 | Epdr2 | ependymin related protein 2 (zebrafish) | 224.8 | 495.5 | 0.45 |
| 12845 | Comp | cartilage oligomeric matrix protein | 52.7 | 116.7 | 0.45 |
| 19883 | Rora | RAR-related orphan receptor alpha | 63.0 | 139.0 | 0.45 |
| 233752 | 3830422K02Rik | RIKEN cDNA 3830422K02 gene | 21.2 | 46.5 | 0.45 |
| 20287 | Sct | secretin | 293.1 | 647.7 | 0.45 |
| 234353 | 4931420C21Rik | RIKEN cDNA 4931420C21 gene | 28.9 | 62.7 | 0.45 |
| 14619 | Gjb2 | gap junction membrane channel protein beta 2 | 46.8 | 103.4 | 0.45 |
| 243923 | Rgs9bp | regulator of G-protein signalling 9 binding protein | 18.2 | 39.9 | 0.45 |
| 26561 | Mmp23 | matrix metallopeptidase 23 | 97.1 | 214.9 | 0.45 |
| 72693 | Zcchc12 | zinc finger, CCHC domain containing 12 | 57.7 | 127.3 | 0.45 |
| 15444 | Hpca | hippocalcin | 37.5 | 82.3 | 0.45 |
| 60344 | Fign | fidgetin | 79.6 | 175.9 | 0.45 |
| 106952 | Centd3 | centaurin, delta 3 | 276.2 | 609.1 | 0.45 |
| 94352 | Loxl2 | lysyl oxidase-like 2 | 382.6 | 844.0 | 0.45 |
| 69585 | Hfe2 | hemochromatosis type 2 (juvenile) (human homolog) | 75.0 | 165.2 | 0.45 |
| 18845 | Plxna2 | plexin A2 | 45.4 | 99.6 | 0.45 |
| 68797 | Pdgfrl | platelet-derived growth factor receptor-like | 435.5 | 958.1 | 0.45 |
| 26896 | Crsp2 | cofactor required for Sp1 transcriptional activation, subunit 2 | 24.7 | 53.7 | 0.45 |
| 230316 | Megf9 | multiple EGF-like-domains 9 | 46.5 | 102.0 | 0.46 |
| 71310 | Tbc1d9 | TBC1 domain family, member 9 | 36.9 | 80.6 | 0.46 |
| 237730 | AI595406 | expressed sequence AI595406 | 49.9 | 108.6 | 0.46 |
| 215705 | Arrdc1 | arrestin domain containing 1 | 284.1 | 621.3 | 0.46 |
| 11551 | Adra2a | adrenergic receptor, alpha 2a | 37.9 | 82.1 | 0.46 |
| 223649 | Nrbp2 | nuclear receptor binding protein 2 | 503.3 | 1098.0 | 0.46 |
| 20927 | Abcc8 | ATP-binding cassette, sub-family C (CFTR/MRP), member 8 | 253.5 | 553.7 | 0.46 |
| 68941 | 1110018N20Rik | RIKEN cDNA 1110018N20 gene | 19.1 | 41.4 | 0.46 |
| 11785 | Apbb1 | amyloid beta (A4) precursor protein-binding, family B, member 1 | 253.2 | 551.5 | 0.46 |
| 29877 | Hdgfrp3 | hepatoma-derived growth factor, related protein 3 | 93.8 | 203.3 | 0.46 |
| 11504 | Adamts1 | a disintegrin-like and metallopeptidase (reprolysin type) with thrombospondin type 1 motif, 1 | 15.4 | 33.3 | 0.46 |
| 243771 | Parp12 | poly (ADP-ribose) polymerase family, member 12 | 96.0 | 208.0 | 0.46 |
| 19416 | Rasd1 | RAS, dexamethasone-induced 1 | 71.0 | 153.9 | 0.46 |
| 140703 | Emid1 | EMI domain containing 1 | 270.6 | 586.0 | 0.46 |
| 18767 | Pkia | protein kinase inhibitor, alpha | 30.1 | 65.2 | 0.46 |
| 67608 | Narf | nuclear prelamin A recognition factor | 160.3 | 346.6 | 0.46 |
| 244579 | Tnrc9 | trinucleotide repeat containing 9 | 111.8 | 242.4 | 0.46 |
| 229003 | BC006779 | cDNA sequence BC006779 | 16.3 | 35.3 | 0.46 |
| 69195 | Tmem121 | transmembrane protein 121 | 4396.3 | 9522.3 | 0.46 |
| 93761 | Smarca1 | SWI/SNF related, matrix associated, actin dependent regulator of chromatin, subfamily a, member 1 | 650.0 | 1404.9 | 0.46 |
| 99003 | Qser1 | glutamine and serine rich 1 | 896.0 | 1938.0 | 0.46 |
| 544817 | Arhgap27 | Rho GTPase activating protein 27 | 246.2 | 532.8 | 0.46 |
| 217310 | C630004H02Rik | RIKEN cDNA C630004H02 gene | 35.7 | 77.1 | 0.46 |
| 59036 | Dact1 | dapper homolog 1, antagonist of beta-catenin (xenopus) | 210.2 | 454.3 | 0.46 |
| 21827 | Thbs3 | thrombospondin 3 | 1257.4 | 2717.1 | 0.46 |
| 66970 | Ssbp2 | single-stranded DNA binding protein 2 | 154.0 | 333.0 | 0.46 |
| 30052 | Pcsk1n | proprotein convertase subtilisin/kexin type 1 inhibitor | 1218.4 | 2629.7 | 0.46 |
| 70821 | 4921507P07Rik | RIKEN cDNA 4921507P07 gene | 23.3 | 50.2 | 0.46 |
| 20440 | St6gal1 | beta galactoside alpha 2,6 sialyltransferase 1 | 171.9 | 370.9 | 0.46 |
| 50876 | Tmod2 | tropomodulin 2 | 423.2 | 913.0 | 0.46 |
| 71069 | Stox2 | storkhead box 2 | 167.7 | 360.1 | 0.46 |
| 238455 | 4732474O15Rik | RIKEN cDNA 4732474O15 gene | 33.2 | 71.1 | 0.46 |
| 16842 | Lef1 | lymphoid enhancer binding factor 1 | 1308.3 | 2822.3 | 0.46 |
| 232975 | Atp1a3 | ATPase, Na+/K+ transporting, alpha 3 polypeptide | 513.1 | 1105.1 | 0.46 |
| 70152 | Mettl7a | methyltransferase like 7A | 166.2 | 358.3 | 0.46 |
| 56430 | Rsn | restin (Reed-Steinberg cell-expressed intermediate filament-associated protein) | 524.5 | 1129.2 | 0.46 |
| 207798 | Gramd1c | GRAM domain containing 1C | 33.8 | 72.8 | 0.46 |
| 27223 | Trp53bp1 | transformation related protein 53 binding protein 1 | 2484.1 | 5348.2 | 0.46 |
| 13640 | Efna5 | ephrin A5 | 245.0 | 526.5 | 0.46 |
| 195208 | Dcdc2a | doublecortin domain containing 2a | 17.5 | 37.0 | 0.46 |
| 19283 | Ptprz1 | protein tyrosine phosphatase, receptor type Z, polypeptide 1 | 21.1 | 45.3 | 0.46 |
| 329406 | 5230400M03Rik | RIKEN cDNA 5230400M03 gene | 16.3 | 34.9 | 0.47 |
| 67254 | 2900011O08Rik | RIKEN cDNA 2900011O08 gene | 483.0 | 1036.2 | 0.47 |
| 81905 | Cacng8 | calcium channel, voltage-dependent, gamma subunit 8 | 44.5 | 94.6 | 0.47 |
| 630499 | LOC630499 | similar to H-2 class I histocompatibility antigen, D-K alpha chain precursor (H-2D(K)) | 2664.4 | 5709.2 | 0.47 |
| 78473 | Scap1 | src family associated phosphoprotein 1 | 22.4 | 47.9 | 0.47 |
| 66259 | Camk2n1 | calcium/calmodulin-dependent protein kinase II inhibitor 1 | 104.7 | 223.3 | 0.47 |
| 21678 | Tead3 | TEA domain family member 3 | 18.7 | 39.5 | 0.47 |
| 14964 | H2-D1 | histocompatibility 2, D region locus 1 | 2968.9 | 6337.3 | 0.47 |
| 246154 | Vasn | vasorin | 241.9 | 515.2 | 0.47 |
| 50790 | Acsl4 | acyl-CoA synthetase long-chain family member 4 | 2459.2 | 5226.9 | 0.47 |
| 56636 | Fgf21 | fibroblast growth factor 21 | 25.6 | 54.3 | 0.47 |
| 13036 | Ctsh | cathepsin H | 63.5 | 135.0 | 0.47 |
| 246316 | Lgi2 | leucine-rich repeat LGI family, member 2 | 180.0 | 382.3 | 0.47 |
| 71904 | Paqr7 | progestin and adipoQ receptor family member VII | 52.3 | 111.1 | 0.47 |
| 213019 | Pdlim2 | PDZ and LIM domain 2 | 555.0 | 1178.2 | 0.47 |
| 320207 | Pik3r5 | phosphoinositide-3-kinase, regulatory subunit 5, p101 | 40.6 | 86.0 | 0.47 |
| 225362 | Reep2 | receptor accessory protein 2 | 245.9 | 519.1 | 0.47 |
| 213121 | Ankrd35 | ankyrin repeat domain 35 | 1017.4 | 2156.1 | 0.47 |
| 12496 | Entpd2 | ectonucleoside triphosphate diphosphohydrolase 2 | 210.4 | 445.1 | 0.47 |
| 213649 | Arhgef19 | Rho guanine nucleotide exchange factor (GEF) 19 | 112.0 | 237.3 | 0.47 |
| 277396 | Klhl23 | kelch-like 23 (Drosophila) | 113.3 | 239.9 | 0.47 |
| 66643 | Lix1 | limb expression 1 homolog (chicken) | 27.6 | 58.3 | 0.47 |
| 13483 | Dpp6 | dipeptidylpeptidase 6 | 34.4 | 72.4 | 0.47 |
| 84652 | Drctnnb1a | down-regulated by Ctnnb1, a | 382.1 | 806.3 | 0.47 |
| 319876 | Cobll1 | Cobl-like 1 | 31.6 | 65.8 | 0.47 |
| 68149 | Otub2 | OTU domain, ubiquitin aldehyde binding 2 | 244.8 | 517.3 | 0.47 |
| 433886 | LOC433886 | hypothetical gene supported by AK049058; BC025881 | 16.7 | 35.3 | 0.47 |
| 319749 | C230078M08Rik | RIKEN cDNA C230078M08 gene | 132.4 | 279.5 | 0.47 |
| 16011 | Igfbp5 | insulin-like growth factor binding protein 5 | 54.4 | 114.5 | 0.47 |
| 76415 | Tmem162 | transmembrane protein 162 | 36.7 | 77.0 | 0.47 |
| 15902 | Id2 | inhibitor of DNA binding 2 | 336.7 | 707.9 | 0.47 |
| 218232 | AW456874 | expressed sequence AW456874 | 25.4 | 53.4 | 0.47 |
| 227733 | Pip5kl1 | phosphatidylinositol-4-phosphate 5-kinase-like 1 | 41.2 | 86.8 | 0.47 |
| 245269 | E130304F04Rik | RIKEN cDNA E130304F04 gene | 27.2 | 56.4 | 0.47 |
| 230587 | Glis1 | GLIS family zinc finger 1 | 69.9 | 147.0 | 0.48 |
| 21974 | Top2b | topoisomerase (DNA) II beta | 2712.0 | 5689.2 | 0.48 |
| 74053 | Grip1 | glutamate receptor interacting protein 1 | 155.5 | 326.7 | 0.48 |
| 15006 | H2-Q1 | histocompatibility 2, Q region locus 1 | 2950.3 | 6207.6 | 0.48 |
| 14570 | Arhgdig | Rho GDP dissociation inhibitor (GDI) gamma | 1053.8 | 2214.7 | 0.48 |
| 18441 | P2ry1 | purinergic receptor P2Y, G-protein coupled 1 | 41.5 | 87.1 | 0.48 |
| 83921 | Tmem2 | transmembrane protein 2 | 1066.3 | 2236.9 | 0.48 |
| 18189 | Nrxn1 | neurexin I | 26.2 | 54.8 | 0.48 |
| 15018 | H2-Q7 | histocompatibility 2, Q region locus 7 | 1510.5 | 3165.5 | 0.48 |
| 18616 | Peg3 | paternally expressed 3 | 681.3 | 1428.8 | 0.48 |
| 399612 | 9630010G10Rik | RIKEN cDNA 9630010G10 gene | 101.2 | 212.1 | 0.48 |
| 20616 | Snap91 | synaptosomal-associated protein 91 | 33.8 | 70.0 | 0.48 |
| 218630 | Ung2 | uracil DNA glycosylase 2 | 747.0 | 1563.3 | 0.48 |
| 27390 | Mmel1 | membrane metallo-endopeptidase-like 1 | 103.7 | 215.5 | 0.48 |
| 19267 | Ptpre | protein tyrosine phosphatase, receptor type, E | 46.2 | 96.0 | 0.48 |
| 83603 | Elovl4 | elongation of very long chain fatty acids (FEN1/Elo2, SUR4/Elo3, yeast)-like 4 | 131.7 | 275.1 | 0.48 |
| 11504 | Adamts1 | a disintegrin-like and metallopeptidase (reprolysin type) with thrombospondin type 1 motif, 1 | 123.1 | 257.1 | 0.48 |
| 108089 | Rnf144 | ring finger protein 144 | 73.0 | 152.3 | 0.48 |
| 232670 | Tspan33 | tetraspanin 33 | 44.8 | 92.7 | 0.48 |
| 75036 | 4930488B01Rik | RIKEN cDNA 4930488B01 gene | 37.1 | 77.5 | 0.48 |
| 76681 | Trim12 | tripartite motif protein 12 | 316.9 | 660.1 | 0.48 |
| 52850 | Rutbc2 | RUN and TBC1 domain containing 2 | 745.7 | 1552.9 | 0.48 |
| 246707 | Emilin2 | elastin microfibril interfacer 2 | 175.5 | 365.4 | 0.48 |
| 56296 | Dmrtb1 | DMRT-like family B with proline-rich C-terminal, 1 | 52.7 | 109.6 | 0.48 |
| 246228 | Vwa1 | von Willebrand factor A domain containing 1 | 78.4 | 163.1 | 0.48 |
| 215821 | D10Bwg1379e | DNA segment, Chr 10, Brigham & Women's Genetics 1379 expressed | 19.2 | 40.0 | 0.48 |
| 12258 | Serping1 | serine (or cysteine) peptidase inhibitor, clade G, member 1 | 174.0 | 362.0 | 0.48 |
| 69274 | Ctdspl | CTD (carboxy-terminal domain, RNA polymerase II, polypeptide A) small phosphatase-like | 147.1 | 305.9 | 0.48 |
| 15040 | H2-T23 | histocompatibility 2, T region locus 23 | 1707.6 | 3539.4 | 0.48 |
| 20359 | Sema6b | sema domain, transmembrane domain (TM), and cytoplasmic domain, (semaphorin) 6B | 68.6 | 141.9 | 0.48 |
| 16975 | Lrp8 | low density lipoprotein receptor-related protein 8, apolipoprotein e receptor | 122.1 | 252.4 | 0.48 |
| 230863 | Sh2d5 | SH2 domain containing 5 | 89.4 | 184.7 | 0.48 |
| 668212 | LOC668212 | similar to RIKEN cDNA C920006C10 | 111.7 | 229.5 | 0.48 |
| 668303 | LOC668303 | similar to CG14535-PA | 340.5 | 702.7 | 0.48 |
| 217517 | Stxbp6 | syntaxin binding protein 6 (amisyn) | 79.4 | 163.1 | 0.48 |
| 81904 | Cacng7 | calcium channel, voltage-dependent, gamma subunit 7 | 68.5 | 141.2 | 0.48 |
| 27419 | Naglu | alpha-N-acetylglucosaminidase (Sanfilippo disease IIIB) | 109.2 | 225.2 | 0.48 |
| 66066 | Gng11 | guanine nucleotide binding protein (G protein), gamma 11 | 169.7 | 349.8 | 0.49 |
| 20741 | Spnb1 | spectrin beta 1 | 18.5 | 38.0 | 0.49 |
| 17000 | Ltbr | lymphotoxin B receptor | 212.2 | 434.3 | 0.49 |
| 98733 | AW822216 | expressed sequence AW822216 | 586.8 | 1207.2 | 0.49 |
| 320204 | 4833442J19Rik | RIKEN cDNA 4833442J19 gene | 177.1 | 364.3 | 0.49 |
| 331623 | AK122525 | cDNA sequence AK122525 | 6983.1 | 14375.2 | 0.49 |
| 319666 | D630014A15Rik | RIKEN cDNA D630014A15 gene | 35.9 | 72.9 | 0.49 |
| 98365 | Slamf9 | SLAM family member 9 | 19.0 | 38.9 | 0.49 |
| 20866 | Stim1 | stromal interaction molecule 1 | 800.5 | 1639.9 | 0.49 |
| 16161 | Il12rb1 | interleukin 12 receptor, beta 1 | 67.0 | 136.3 | 0.49 |
| 72480 | Tspyl4 | TSPY-like 4 | 132.0 | 268.9 | 0.49 |
| 230678 | 6330530A05Rik | RIKEN cDNA 6330530A05 gene | 424.5 | 867.0 | 0.49 |
| 60525 | Acss2 | acyl-CoA synthetase short-chain family member 2 | 92.1 | 187.5 | 0.49 |
| 57765 | Tbx21 | T-box 21 | 106.0 | 215.2 | 0.49 |
| 27387 | Sh2d3c | SH2 domain containing 3C | 150.5 | 307.0 | 0.49 |
| 13808 | Eno3 | enolase 3, beta muscle | 2680.4 | 5466.2 | 0.49 |
| 15937 | Ier3 | immediate early response 3 | 739.8 | 1502.8 | 0.49 |
| 233335 | Dmn | desmuslin | 25.9 | 52.5 | 0.49 |
| 56628 | LOC56628 | MHC (A.CA/J(H-2K-f) class I antigen | 3521.7 | 7166.6 | 0.49 |
| 19122 | Prnp | prion protein | 259.2 | 526.4 | 0.49 |
| 74777 | Sepn1 | selenoprotein N, 1 | 218.4 | 444.0 | 0.49 |
| 15900 | Irf8 | interferon regulatory factor 8 | 97.0 | 196.7 | 0.49 |
| 243931 | Tshz3 | teashirt zinc finger family member 3 | 47.5 | 96.5 | 0.49 |
| 269615 | Plcl4 | phospholipase C-like 4 | 46.3 | 93.4 | 0.49 |
| 70911 | Phyhipl | phytanoyl-CoA hydroxylase interacting protein-like | 16.9 | 34.0 | 0.49 |
| 71532 | 9030418K01Rik | RIKEN cDNA 9030418K01 gene | 110.4 | 223.5 | 0.49 |
| 330863 | Trim67 | tripartite motif-containing 67 | 21.8 | 44.1 | 0.49 |
| 12759 | Clu | clusterin | 549.4 | 1112.5 | 0.49 |
| 53623 | Gria3 | glutamate receptor, ionotropic, AMPA3 (alpha 3) | 27.0 | 54.4 | 0.49 |
| 16450 | Jag2 | jagged 2 | 301.6 | 609.7 | 0.49 |
| 56320 | Dbn1 | drebrin 1 | 752.7 | 1523.1 | 0.49 |
| 212943 | BC023892 | cDNA sequence BC023892 | 88.4 | 178.4 | 0.49 |
| 67557 | Larp6 | La ribonucleoprotein domain family, member 6 | 27.0 | 54.2 | 0.49 |
| 15979 | Ifngr1 | interferon gamma receptor 1 | 936.4 | 1890.3 | 0.49 |
| 194126 | Mtmr11 | myotubularin related protein 11 | 35.2 | 71.3 | 0.49 |
| 276952 | Rasl10b | RAS-like, family 10, member B | 44.0 | 88.6 | 0.49 |
| 66857 | 1100001H23Rik | RIKEN cDNA 1100001H23 gene | 35.2 | 71.0 | 0.50 |
| 56517 | Slc22a21 | solute carrier family 22 (organic cation transporter), member 21 | 46.1 | 92.8 | 0.50 |
| 83561 | Tdrd1 | tudor domain containing 1 | 195.9 | 394.6 | 0.50 |
| 142682 | Zcchc14 | zinc finger, CCHC domain containing 14 | 74.7 | 149.3 | 0.50 |
| 14412 | Slc6a13 | solute carrier family 6 (neurotransmitter transporter, GABA), member 13 | 38.8 | 77.8 | 0.50 |
| 69065 | Chac1 | ChaC, cation transport regulator-like 1 (E. coli) | 5048.2 | 10128.2 | 0.50 |
| 140580 | Elmo1 | engulfment and cell motility 1, ced-12 homolog (C. elegans) | 19.7 | 39.3 | 0.50 |
| 14972 | H2-K1 | histocompatibility 2, K1, K region | 761.8 | 1529.4 | 0.50 |
| 69066 | 1810010H24Rik | RIKEN cDNA 1810010H24 gene | 35.5 | 71.0 | 0.50 |
| 18783 | Pla2g4a | phospholipase A2, group IVA (cytosolic, calcium-dependent) | 70.6 | 140.7 | 0.50 |
| 217578 | Baz1a | bromodomain adjacent to zinc finger domain 1A | 3116.2 | 6234.5 | 0.50 |
