# Supplementary material for: Stable generation of serum- and feeder-free embryonic stem cell-derived mice with full germline-competency by using a GSK3 specific inhibitor
Source: Genesis. 2009 Apr 23;47(6):414–22. doi: 10.1002/dvg.20514 (PMC2726955; doi:10.1002/dvg.20514)
Supplement: Supplementary file 9 [file dvg0047-0414-SD9.doc]

Supplementary Table 3

| **Category** | **Genes in Category** | **% of Genes in Category** | **Genes in List in Category** | **% of Genes in List in Category** | **p-Value** |
| --- | --- | --- | --- | --- | --- |
| GO:16565: general transcriptional repressor activity | 7 | 0.0272 | 5 | 1.333 | 1.31E-08 |
| GO:3700: transcription factor activity | 1482 | 5.754 | 45 | 12 | 2.64E-06 |
| GO:30528: transcription regulator activity | 2151 | 8.351 | 58 | 15.47 | 3.66E-06 |
| GO:16504: protease activator activity | 25 | 0.0971 | 5 | 1.333 | 2.66E-05 |
| GO:4111: creatine kinase activity | 5 | 0.0194 | 3 | 0.8 | 3.00E-05 |
| GO:1871: pattern binding | 176 | 0.683 | 11 | 2.933 | 5.79E-05 |
| GO:5539: glycosaminoglycan binding | 152 | 0.59 | 10 | 2.667 | 8.16E-05 |
| GO:3774: motor activity | 292 | 1.134 | 14 | 3.733 | 0.000108 |
| GO:30247: polysaccharide binding | 166 | 0.644 | 10 | 2.667 | 0.000169 |
| GO:5102: receptor binding | 908 | 3.525 | 28 | 7.467 | 0.000174 |
| GO:43121: neurotrophin binding | 2 | 0.00776 | 2 | 0.533 | 0.000211 |
| GO:48406: nerve growth factor binding | 2 | 0.00776 | 2 | 0.533 | 0.000211 |
| GO:8201: heparin binding | 121 | 0.47 | 8 | 2.133 | 0.000404 |
| GO:16806: dipeptidyl-peptidase and tripeptidyl-peptidase activity | 25 | 0.0971 | 4 | 1.067 | 0.000439 |
| GO:8239: dipeptidyl-peptidase activity | 25 | 0.0971 | 4 | 1.067 | 0.000439 |
| GO:16564: transcriptional repressor activity | 307 | 1.192 | 13 | 3.467 | 0.000617 |
| GO:3836: beta-galactoside alpha-2,3-sialyltransferase activity | 3 | 0.0116 | 2 | 0.533 | 0.000628 |
| GO:4567: beta-mannosidase activity | 3 | 0.0116 | 2 | 0.533 | 0.000628 |
| GO:15220: choline transporter activity | 3 | 0.0116 | 2 | 0.533 | 0.000628 |
| GO:4287: prolyl oligopeptidase activity | 13 | 0.0505 | 3 | 0.8 | 0.000786 |
| GO:3858: 3-hydroxybutyrate dehydrogenase activity | 4 | 0.0155 | 2 | 0.533 | 0.00124 |
| GO:3953: NAD+ nucleosidase activity | 4 | 0.0155 | 2 | 0.533 | 0.00124 |
| GO:8494: translation activator activity | 4 | 0.0155 | 2 | 0.533 | 0.00124 |
| GO:4084: branched-chain-amino-acid transaminase activity | 5 | 0.0194 | 2 | 0.533 | 0.00205 |
| GO:8195: phosphatidate phosphatase activity | 5 | 0.0194 | 2 | 0.533 | 0.00205 |
| GO:4274: dipeptidyl-peptidase IV activity | 18 | 0.0699 | 3 | 0.8 | 0.00212 |
| GO:30742: GTP-dependent protein binding | 21 | 0.0815 | 3 | 0.8 | 0.00335 |
| GO:5519: cytoskeletal regulatory protein binding | 22 | 0.0854 | 3 | 0.8 | 0.00384 |
| GO:4095: carnitine O-palmitoyltransferase activity | 7 | 0.0272 | 2 | 0.533 | 0.00423 |
| GO:16416: O-palmitoyltransferase activity | 7 | 0.0272 | 2 | 0.533 | 0.00423 |
| GO:4718: Janus kinase activity | 7 | 0.0272 | 2 | 0.533 | 0.00423 |
| GO:4963: follicle stimulating hormone receptor activity | 7 | 0.0272 | 2 | 0.533 | 0.00423 |
| GO:42813: Wnt receptor activity | 7 | 0.0272 | 2 | 0.533 | 0.00423 |
| GO:3730: mRNA 3'-UTR binding | 7 | 0.0272 | 2 | 0.533 | 0.00423 |
| GO:4716: receptor signaling protein tyrosine kinase activity | 8 | 0.0311 | 2 | 0.533 | 0.00559 |
| GO:30506: ankyrin binding | 8 | 0.0311 | 2 | 0.533 | 0.00559 |
| GO:8367: bacterial binding | 8 | 0.0311 | 2 | 0.533 | 0.00559 |
| GO:8083: growth factor activity | 229 | 0.889 | 9 | 2.4 | 0.00664 |
| GO:5243: gap-junction forming channel activity | 27 | 0.105 | 3 | 0.8 | 0.00691 |
| GO:15285: connexon channel activity | 27 | 0.105 | 3 | 0.8 | 0.00691 |
| GO:5488: binding | 18524 | 71.92 | 291 | 77.6 | 0.00708 |
| GO:3980: UDP-glucose:glycoprotein glucosyltransferase activity | 9 | 0.0349 | 2 | 0.533 | 0.00711 |
| GO:19864: IgG binding | 9 | 0.0349 | 2 | 0.533 | 0.00711 |
| GO:8092: cytoskeletal protein binding | 752 | 2.92 | 20 | 5.333 | 0.0075 |
| GO:3677: DNA binding | 3379 | 13.12 | 66 | 17.6 | 0.00759 |
| GO:16406: carnitine O-acyltransferase activity | 10 | 0.0388 | 2 | 0.533 | 0.00881 |
| GO:4415: hyalurononglucosaminidase activity | 10 | 0.0388 | 2 | 0.533 | 0.00881 |
| GO:5035: death receptor activity | 10 | 0.0388 | 2 | 0.533 | 0.00881 |
| GO:8301: DNA bending activity | 10 | 0.0388 | 2 | 0.533 | 0.00881 |
| GO:5328: neurotransmitter:sodium symporter activity | 31 | 0.12 | 3 | 0.8 | 0.0102 |
| GO:5515: protein binding | 9486 | 36.83 | 160 | 42.67 | 0.011 |
| GO:4364: glutathione transferase activity | 33 | 0.128 | 3 | 0.8 | 0.0121 |
| GO:3707: steroid hormone receptor activity | 94 | 0.365 | 5 | 1.333 | 0.0122 |
| GO:50811: GABA receptor binding | 12 | 0.0466 | 2 | 0.533 | 0.0127 |
| GO:4879: ligand-dependent nuclear receptor activity | 95 | 0.369 | 5 | 1.333 | 0.0127 |
| GO:5326: neurotransmitter transporter activity | 35 | 0.136 | 3 | 0.8 | 0.0142 |
| GO:8743: L-threonine 3-dehydrogenase activity | 1 | 0.00388 | 1 | 0.267 | 0.0146 |
| GO:16603: glutaminyl-peptide cyclotransferase activity | 1 | 0.00388 | 1 | 0.267 | 0.0146 |
| GO:47325: inositol tetrakisphosphate 1-kinase activity | 1 | 0.00388 | 1 | 0.267 | 0.0146 |
| GO:4214: dipeptidyl-peptidase I activity | 1 | 0.00388 | 1 | 0.267 | 0.0146 |
| GO:31705: bombesin receptor binding | 1 | 0.00388 | 1 | 0.267 | 0.0146 |
| GO:31707: endothelin A receptor binding | 1 | 0.00388 | 1 | 0.267 | 0.0146 |
| GO:5309: creatine:sodium symporter activity | 1 | 0.00388 | 1 | 0.267 | 0.0146 |
| GO:5308: creatine transporter activity | 1 | 0.00388 | 1 | 0.267 | 0.0146 |
| GO:4293: tissue kallikrein activity | 13 | 0.0505 | 2 | 0.533 | 0.0148 |
| GO:43015: gamma-tubulin binding | 13 | 0.0505 | 2 | 0.533 | 0.0148 |
| GO:8373: sialyltransferase activity | 40 | 0.155 | 3 | 0.8 | 0.0203 |
| GO:3909: DNA ligase activity | 16 | 0.0621 | 2 | 0.533 | 0.0222 |
| GO:3910: DNA ligase (ATP) activity | 16 | 0.0621 | 2 | 0.533 | 0.0222 |
| GO:1619: lysosphingolipid and lysophosphatidic acid receptor activity | 16 | 0.0621 | 2 | 0.533 | 0.0222 |
| GO:45125: bioactive lipid receptor activity | 17 | 0.066 | 2 | 0.533 | 0.0249 |
| GO:19865: immunoglobulin binding | 17 | 0.066 | 2 | 0.533 | 0.0249 |
| GO:5048: signal sequence binding | 18 | 0.0699 | 2 | 0.533 | 0.0277 |
| GO:50528: acyloxyacyl hydrolase activity | 2 | 0.00776 | 1 | 0.267 | 0.0289 |
| GO:4490: methylglutaconyl-CoA hydratase activity | 2 | 0.00776 | 1 | 0.267 | 0.0289 |
| GO:247: C-8 sterol isomerase activity | 2 | 0.00776 | 1 | 0.267 | 0.0289 |
| GO:47750: cholestenol delta-isomerase activity | 2 | 0.00776 | 1 | 0.267 | 0.0289 |
| GO:16987: sigma factor activity | 2 | 0.00776 | 1 | 0.267 | 0.0289 |
| GO:4926: non-G-protein coupled 7TM receptor activity | 19 | 0.0738 | 2 | 0.533 | 0.0307 |
| GO:3779: actin binding | 499 | 1.937 | 13 | 3.467 | 0.0322 |
| GO:35251: UDP-glucosyltransferase activity | 20 | 0.0776 | 2 | 0.533 | 0.0338 |
| GO:16500: protein-hormone receptor activity | 20 | 0.0776 | 2 | 0.533 | 0.0338 |
| GO:16702: oxidoreductase activity, acting on single donors with incorporation of molecular oxygen, incorporation of two atoms of oxygen | 85 | 0.33 | 4 | 1.067 | 0.0357 |
| GO:51213: dioxygenase activity | 85 | 0.33 | 4 | 1.067 | 0.0357 |
| GO:16701: oxidoreductase activity, acting on single donors with incorporation of molecular oxygen | 86 | 0.334 | 4 | 1.067 | 0.037 |
| GO:16755: transferase activity, transferring amino-acyl groups | 21 | 0.0815 | 2 | 0.533 | 0.037 |
| GO:46527: glucosyltransferase activity | 21 | 0.0815 | 2 | 0.533 | 0.037 |
| GO:4693: cyclin-dependent protein kinase activity | 21 | 0.0815 | 2 | 0.533 | 0.037 |
| GO:16775: phosphotransferase activity, nitrogenous group as acceptor | 52 | 0.202 | 3 | 0.8 | 0.0401 |
| GO:42296: ISG15 conjugating enzyme activity | 3 | 0.0116 | 1 | 0.267 | 0.043 |
| GO:4800: thyroxine 5'-deiodinase activity | 3 | 0.0116 | 1 | 0.267 | 0.043 |
| GO:4666: prostaglandin-endoperoxide synthase activity | 3 | 0.0116 | 1 | 0.267 | 0.043 |
| GO:4238: meprin A activity | 3 | 0.0116 | 1 | 0.267 | 0.043 |
| GO:4035: alkaline phosphatase activity | 3 | 0.0116 | 1 | 0.267 | 0.043 |
| GO:8800: beta-lactamase activity | 3 | 0.0116 | 1 | 0.267 | 0.043 |
| GO:16019: peptidoglycan receptor activity | 3 | 0.0116 | 1 | 0.267 | 0.043 |
| GO:8519: ammonium transporter activity | 3 | 0.0116 | 1 | 0.267 | 0.043 |
| GO:5003: ephrin receptor activity | 23 | 0.0893 | 2 | 0.533 | 0.0437 |
| GO:5516: calmodulin binding | 223 | 0.866 | 7 | 1.867 | 0.0455 |
| GO:19838: growth factor binding | 93 | 0.361 | 4 | 1.067 | 0.0471 |
| GO:15929: hexosaminidase activity | 24 | 0.0932 | 2 | 0.533 | 0.0473 |
