# Supplementary material for: Stable generation of serum- and feeder-free embryonic stem cell-derived mice with full germline-competency by using a GSK3 specific inhibitor
Source: Genesis. 2009 Apr 23;47(6):414–22. doi: 10.1002/dvg.20514 (PMC2726955; doi:10.1002/dvg.20514)
Supplement: Supplementary file 10 [file dvg0047-0414-SD10.doc]

Supplementary Table 4

| **Category** | **Genes in Category** | **% of Genes in Category** | **Genes in List in Category** | **% of Genes in List in Category** | **p-Value** |
| --- | --- | --- | --- | --- | --- |
| GO:5509: calcium ion binding | 1418 | 5.505 | 79 | 9.962 | 2.57E-07 |
| GO:5515: protein binding | 9486 | 36.83 | 350 | 44.14 | 1.06E-05 |
| GO:4467: long-chain-fatty-acid-CoA ligase activity | 21 | 0.0815 | 6 | 0.757 | 3.05E-05 |
| GO:15645: fatty-acid ligase activity | 23 | 0.0893 | 6 | 0.757 | 5.39E-05 |
| GO:16877: ligase activity, forming carbon-sulfur bonds | 35 | 0.136 | 7 | 0.883 | 8.09E-05 |
| GO:42606: endogenous peptide antigen binding | 4 | 0.0155 | 3 | 0.378 | 0.000114 |
| GO:46982: protein heterodimerization activity | 151 | 0.586 | 14 | 1.765 | 0.000247 |
| GO:3700: transcription factor activity | 1482 | 5.754 | 70 | 8.827 | 0.000255 |
| GO:4938: alpha2-adrenergic receptor activity | 5 | 0.0194 | 3 | 0.378 | 0.000278 |
| GO:30528: transcription regulator activity | 2151 | 8.351 | 93 | 11.73 | 0.000537 |
| GO:3886: DNA (cytosine-5-)-methyltransferase activity | 6 | 0.0233 | 3 | 0.378 | 0.000542 |
| GO:9008: DNA-methyltransferase activity | 6 | 0.0233 | 3 | 0.378 | 0.000542 |
| GO:8307: structural constituent of muscle | 25 | 0.0971 | 5 | 0.631 | 0.000869 |
| GO:4528: phosphodiesterase I activity | 7 | 0.0272 | 3 | 0.378 | 0.000927 |
| GO:4991: parathyroid hormone receptor activity | 2 | 0.00776 | 2 | 0.252 | 0.000947 |
| GO:30227: apolipoprotein E receptor activity | 2 | 0.00776 | 2 | 0.252 | 0.000947 |
| GO:30160: GKAP/Homer scaffold activity | 2 | 0.00776 | 2 | 0.252 | 0.000947 |
| GO:4089: carbonate dehydratase activity | 27 | 0.105 | 5 | 0.631 | 0.00126 |
| GO:42605: peptide antigen binding | 17 | 0.066 | 4 | 0.504 | 0.00154 |
| GO:5262: calcium channel activity | 125 | 0.485 | 11 | 1.387 | 0.00169 |
| GO:30106: MHC class I receptor activity | 29 | 0.113 | 5 | 0.631 | 0.00176 |
| GO:4926: non-G-protein coupled 7TM receptor activity | 19 | 0.0738 | 4 | 0.504 | 0.00239 |
| GO:4051: arachidonate 5-lipoxygenase activity | 3 | 0.0116 | 2 | 0.252 | 0.00278 |
| GO:8900: hydrogen:potassium-exchanging ATPase activity | 3 | 0.0116 | 2 | 0.252 | 0.00278 |
| GO:30226: apolipoprotein receptor activity | 3 | 0.0116 | 2 | 0.252 | 0.00278 |
| GO:5261: cation channel activity | 395 | 1.534 | 23 | 2.9 | 0.00284 |
| GO:4936: alpha-adrenergic receptor activity | 10 | 0.0388 | 3 | 0.378 | 0.00297 |
| GO:3828: alpha-N-acetylneuraminate alpha-2,8-sialyltransferase activity | 11 | 0.0427 | 3 | 0.378 | 0.00399 |
| GO:4551: nucleotide diphosphatase activity | 11 | 0.0427 | 3 | 0.378 | 0.00399 |
| GO:5488: binding | 18524 | 71.92 | 603 | 76.04 | 0.00443 |
| GO:3947: (N-acetylneuraminyl)-galactosylglucosylceramide N-acetylgalactosaminyltransferase activity | 4 | 0.0155 | 2 | 0.252 | 0.00545 |
| GO:51400: BH domain binding | 4 | 0.0155 | 2 | 0.252 | 0.00545 |
| GO:51434: BH3 domain binding | 4 | 0.0155 | 2 | 0.252 | 0.00545 |
| GO:8160: protein tyrosine phosphatase activator activity | 4 | 0.0155 | 2 | 0.252 | 0.00545 |
| GO:5094: Rho GDP-dissociation inhibitor activity | 4 | 0.0155 | 2 | 0.252 | 0.00545 |
| GO:5112: Notch binding | 13 | 0.0505 | 3 | 0.378 | 0.0066 |
| GO:4857: enzyme inhibitor activity | 356 | 1.382 | 20 | 2.522 | 0.00756 |
| GO:4622: lysophospholipase activity | 5 | 0.0194 | 2 | 0.252 | 0.0089 |
| GO:15027: coreceptor, soluble ligand activity | 5 | 0.0194 | 2 | 0.252 | 0.0089 |
| GO:50780: dopamine receptor binding | 5 | 0.0194 | 2 | 0.252 | 0.0089 |
| GO:46872: metal ion binding | 6150 | 23.88 | 218 | 27.49 | 0.00933 |
| GO:4860: protein kinase inhibitor activity | 43 | 0.167 | 5 | 0.631 | 0.01 |
| GO:16836: hydro-lyase activity | 79 | 0.307 | 7 | 0.883 | 0.0109 |
| GO:5244: voltage-gated ion channel activity | 273 | 1.06 | 16 | 2.018 | 0.0109 |
| GO:8083: growth factor activity | 229 | 0.889 | 14 | 1.765 | 0.0118 |
| GO:43167: ion binding | 6278 | 24.37 | 221 | 27.87 | 0.0119 |
| GO:3779: actin binding | 499 | 1.937 | 25 | 3.153 | 0.0124 |
| GO:30234: enzyme regulator activity | 1226 | 4.76 | 52 | 6.557 | 0.0126 |
| GO:19208: phosphatase regulator activity | 101 | 0.392 | 8 | 1.009 | 0.0128 |
| GO:3840: gamma-glutamyltransferase activity | 6 | 0.0233 | 2 | 0.252 | 0.0131 |
| GO:5332: gamma-aminobutyric acid:sodium symporter activity | 6 | 0.0233 | 2 | 0.252 | 0.0131 |
| GO:4289: subtilase activity | 47 | 0.182 | 5 | 0.631 | 0.0144 |
| GO:5520: insulin-like growth factor binding | 31 | 0.12 | 4 | 0.504 | 0.0145 |
| GO:8376: acetylgalactosaminyltransferase activity | 48 | 0.186 | 5 | 0.631 | 0.0157 |
| GO:19210: kinase inhibitor activity | 48 | 0.186 | 5 | 0.631 | 0.0157 |
| GO:48503: GPI anchor binding | 171 | 0.664 | 11 | 1.387 | 0.0171 |
| GO:8092: cytoskeletal protein binding | 752 | 2.92 | 34 | 4.288 | 0.0172 |
| GO:1730: 2'-5'-oligoadenylate synthetase activity | 7 | 0.0272 | 2 | 0.252 | 0.0179 |
| GO:15185: L-gamma-aminobutyric acid transporter activity | 7 | 0.0272 | 2 | 0.252 | 0.0179 |
| GO:15198: oligopeptide transporter activity | 7 | 0.0272 | 2 | 0.252 | 0.0179 |
| GO:19211: phosphatase activator activity | 7 | 0.0272 | 2 | 0.252 | 0.0179 |
| GO:1633: secretin-like receptor activity | 35 | 0.136 | 4 | 0.504 | 0.0219 |
| GO:15026: coreceptor activity | 20 | 0.0776 | 3 | 0.378 | 0.0224 |
| GO:4871: signal transducer activity | 4721 | 18.33 | 167 | 21.06 | 0.0258 |
| GO:43169: cation binding | 5691 | 22.09 | 198 | 24.97 | 0.0275 |
| GO:8227: amine receptor activity | 56 | 0.217 | 5 | 0.631 | 0.0287 |
| GO:5283: sodium:amino acid symporter activity | 9 | 0.0349 | 2 | 0.252 | 0.0295 |
| GO:5220: inositol 1,4,5-triphosphate-sensitive calcium-release channel activity | 9 | 0.0349 | 2 | 0.252 | 0.0295 |
| GO:51010: microtubule plus-end binding | 9 | 0.0349 | 2 | 0.252 | 0.0295 |
| GO:17112: Rab guanyl-nucleotide exchange factor activity | 9 | 0.0349 | 2 | 0.252 | 0.0295 |
| GO:5092: GDP-dissociation inhibitor activity | 9 | 0.0349 | 2 | 0.252 | 0.0295 |
| GO:45499: chemorepellant activity | 9 | 0.0349 | 2 | 0.252 | 0.0295 |
| GO:16835: carbon-oxygen lyase activity | 97 | 0.377 | 7 | 0.883 | 0.0302 |
| GO:47130: saccharopine dehydrogenase (NADP+, L-lysine-forming) activity | 1 | 0.00388 | 1 | 0.126 | 0.0308 |
| GO:10010: lysine-ketoglutarate reductase activity | 1 | 0.00388 | 1 | 0.126 | 0.0308 |
| GO:18583: biphenyl-2,3-diol 1,2-dioxygenase activity | 1 | 0.00388 | 1 | 0.126 | 0.0308 |
| GO:15067: amidinotransferase activity | 1 | 0.00388 | 1 | 0.126 | 0.0308 |
| GO:15068: glycine amidinotransferase activity | 1 | 0.00388 | 1 | 0.126 | 0.0308 |
| GO:8466: glycogenin glucosyltransferase activity | 1 | 0.00388 | 1 | 0.126 | 0.0308 |
| GO:3844: 1,4-alpha-glucan branching enzyme activity | 1 | 0.00388 | 1 | 0.126 | 0.0308 |
| GO:30572: phosphatidyltransferase activity | 1 | 0.00388 | 1 | 0.126 | 0.0308 |
| GO:8808: cardiolipin synthase activity | 1 | 0.00388 | 1 | 0.126 | 0.0308 |
| GO:45130: keratan sulfotransferase activity | 1 | 0.00388 | 1 | 0.126 | 0.0308 |
| GO:50659: N-acetylgalactosamine 4-sulfate 6-O-sulfotransferase activity | 1 | 0.00388 | 1 | 0.126 | 0.0308 |
| GO:4215: cathepsin H activity | 1 | 0.00388 | 1 | 0.126 | 0.0308 |
| GO:4228: gelatinase A activity | 1 | 0.00388 | 1 | 0.126 | 0.0308 |
| GO:16292: acyl-CoA thioesterase I activity | 1 | 0.00388 | 1 | 0.126 | 0.0308 |
| GO:4409: homoaconitate hydratase activity | 1 | 0.00388 | 1 | 0.126 | 0.0308 |
| GO:5061: aryl hydrocarbon receptor nuclear translocator activity | 1 | 0.00388 | 1 | 0.126 | 0.0308 |
| GO:30116: glial cell line-derived neurotrophic factor receptor binding | 1 | 0.00388 | 1 | 0.126 | 0.0308 |
| GO:17162: aryl hydrocarbon receptor binding | 1 | 0.00388 | 1 | 0.126 | 0.0308 |
| GO:15373: monovalent anion:sodium symporter activity | 1 | 0.00388 | 1 | 0.126 | 0.0308 |
| GO:8507: sodium:iodide symporter activity | 1 | 0.00388 | 1 | 0.126 | 0.0308 |
| GO:15111: iodide transporter activity | 1 | 0.00388 | 1 | 0.126 | 0.0308 |
| GO:30504: inorganic diphosphate transporter activity | 1 | 0.00388 | 1 | 0.126 | 0.0308 |
| GO:51076: Gram-positive bacterial binding | 1 | 0.00388 | 1 | 0.126 | 0.0308 |
| GO:8427: calcium-dependent protein kinase inhibitor activity | 1 | 0.00388 | 1 | 0.126 | 0.0308 |
| GO:31386: protein tag | 1 | 0.00388 | 1 | 0.126 | 0.0308 |
| GO:8373: sialyltransferase activity | 40 | 0.155 | 4 | 0.504 | 0.0339 |
| GO:8276: protein methyltransferase activity | 79 | 0.307 | 6 | 0.757 | 0.0349 |
| GO:5216: ion channel activity | 550 | 2.135 | 25 | 3.153 | 0.0351 |
| GO:4030: aldehyde dehydrogenase [NAD(P)+] activity | 10 | 0.0388 | 2 | 0.252 | 0.0362 |
| GO:4864: protein phosphatase inhibitor activity | 42 | 0.163 | 4 | 0.504 | 0.0396 |
| GO:4867: serine-type endopeptidase inhibitor activity | 149 | 0.578 | 9 | 1.135 | 0.0413 |
| GO:16165: lipoxygenase activity | 11 | 0.0427 | 2 | 0.252 | 0.0433 |
| GO:1515: opioid peptide activity | 11 | 0.0427 | 2 | 0.252 | 0.0433 |
| GO:5523: tropomyosin binding | 11 | 0.0427 | 2 | 0.252 | 0.0433 |
| GO:5543: phospholipid binding | 377 | 1.464 | 18 | 2.27 | 0.0452 |
| GO:4935: adrenoceptor activity | 27 | 0.105 | 3 | 0.378 | 0.0492 |
| GO:8194: UDP-glycosyltransferase activity | 154 | 0.598 | 9 | 1.135 | 0.0492 |
